# Dynamic Extreme Aneuploidy (DEA) in the vegetable pathogen *Phytophthora capsici* and the potential for rapid asexual evolution

**DOI:** 10.1371/journal.pone.0227250

**Published:** 2020-01-07

**Authors:** Jian Hu, Sandesh Shrestha, Yuxin Zhou, Joann Mudge, Xili Liu, Kurt Lamour

**Affiliations:** 1 College of Agro-grassland Science, Nanjing Agricultural University, Nanjing, China; 2 Department of Plant Pathology, Kansas State University, Manhattan, Kansas, United States of America; 3 College of Plant Protection, Nanjing Agricultural University, Nanjing, China; 4 National Center for Genome Resources, Santa Fe, New Mexico, United States of America; 5 College of Plant Pathology, China Agricultural University, Beijing, China; 6 Department of Entomology and Plant Pathology, The University of Tennessee, Knoxville, Tennessee, United States of America; National Cheng Kung University, TAIWAN

## Abstract

Oomycete plant pathogens are difficult to control and routine genetic research is challenging. A major problem is instability of isolates. Here we characterize >600 field and single zoospore isolates of *Phytophthora capsici* for inheritance of mating type, sensitivity to mefenoxam, chromosome copy number and heterozygous allele frequencies. The A2 mating type was highly unstable with 26% of 241 A2 isolates remaining A2. The A1 mating type was stable. Isolates intermediately resistant to mefenoxam produced fully resistant single-spore progeny. Sensitive isolates remained fully sensitive. Genome re-sequencing of single zoospore isolates revealed extreme aneuploidy; a phenomenon dubbed Dynamic Extreme Aneuploidy (DEA). DEA is characterized by the asexual inheritance of diverse intra-genomic combinations of chromosomal ploidy ranging from 2N to 3N and heterozygous allele frequencies that do not strictly correspond to ploidy. Isolates sectoring on agar media showed dramatically altered heterozygous allele frequencies. DEA can explain the rapid increase of advantageous alleles (e.g. drug resistance), mating type switches and copy neutral loss of heterozygosity (LOH). Although the mechanisms driving DEA are unknown, it can play an important role in adaptation and evolution and seriously hinders all aspects of *P*. *capsici* research.

## Introduction

The genus Phytophthora is home to many destructive and pervasive plant pathogens [1–5]. The 120+ species attack almost all dicot plants and threaten ecosystems and entire plant industries [1–5]. For more than 150 years, scientists have struggled to manage this unwieldy genus and make of it (or, at least one of its members) a model organism. Part of this quest has focused on the vegetable pathogen, *P*. *capsici–*a devastating pathogen of vegetables [2–11]. Ostensibly, an ideal research model, isolates can be immortalized under liquid nitrogen, grow rapidly on simple media, can be easy to make sexual crosses in the laboratory, are often highly pathogenic and make copious spores with little coaxing. The problem is sometimes an isolate will do all the above and sometimes it won’t. The intractability of Phytophthora (or success, depending on your point of view) lies in its plasticity [10, 12–15]. Phytophthora, when needed most (e.g. for a long-term laboratory, greenhouse or field experiments), is entirely unreliable.

The ploidy of oomycetes was debated actively for over 75 years and it wasn’t until Eva Sansome, in a 1961 Letter to Nature, provided convincing photomicrographic evidence of meiosis with *Pythium debaryanum* Hesse that the issue approached resolution [16]. She suggested *P*. *debaryanum* was not unique and subsequent work proved her correct [17]. Her findings, although compelling, were not immediately accepted as many researchers recorded aberrant sexual and asexual inheritance patterns [18].

In 2005, the NSF and USDA jointly funded development of a whole genome reference sequence and a single nucleotide polymorphism (SNP) resource for *P*. *capsici*. With a strong team of scientists, money and motivation; a quality genome, ready for publication, was expected in months. Parents and what appeared to be normal progeny had been produced previously and a dense genetic map (a crucial tool missing from the Phytophthora research inventory) was planned. Events did not unfold as expected and months turned into years. *Phytophthora capsici* carries a significant load of polymorphism in the form of heterozygous SNPs, often 1 every 100bp within a single genome, making assembly difficult [8]. Initially, the construction of a genetic map proved impossible as many loci had aberrant inheritance patterns (e.g. AA x Aa parental combinations produced many ‘aa’ progeny). Finally, it was discovered that short and long tracts (300bp to 1Mbp) of the parent and progenies genomes had spontaneously switched (mitotically) to one or the other parental haplotype–a phenomenon known as copy neutral Loss of Heterozygosity (LOH) [14, 19–21]. Removal of the loci residing in LOH tracts helped produce a detailed molecular map and the annotated genome was published in 2012 [10]. Population studies using SNP markers reveal LOH is common in *P*. *capsici* and other oomycetes at locations worldwide [2, 3, 7, 12, 13, 15, 22, 23].

Until recently, the 917 genetic scaffolds were not arranged into chromosomes (18 linkage groups). In 2017, the map-ordered scaffolds were used to visualize heterozygous allele frequencies for *P*. *capsici* recovered from pepper in Taiwan and isolates of the closely related pathogen of taro, *P*. *colocasiae*, from Hawaii, Vietnam, China and Nepal [22, 23]. Allele frequencies measured using whole genome and targeted-sequencing suggested ploidy varied by chromosome within individual isolates. Our goal was to characterize diversity during asexual reproduction and growth by examining asexually derived uni-nucleate zoospore progeny and isolates exhibiting altered growth rates (sectoring) on agar media. Variation was characterized by measuring the sequencing depth and the distribution of heterozygous SNP allele frequencies across chromosomes and by tracking the stability of mating type and variation in sensitivity to the phytophthora-toxic chemical mefenoxam.

## Materials and methods

### Isolates, zoospore progeny, mating type and mefenoxam sensitivity

Field isolates were recovered using standard techniques where a small section of infected plant tissue is plated onto agar media amended with antibiotics and antifungal compounds as previously described [8]. The recovery of single zoospore progeny is as previously described where agar plates are flooded, the plates briefly chilled and resulting swimming spores are induced to encyst and allowed to germinate on water agar prior to retrieval under a light microscope using a needle [8]. A subset of five isolates were recovered from either fast or slow growing sectors visible when growing a single-spore derived isolate on agar media (S1 Table, S1 Fig). Mating type was assessed as previously described using known A1 and A2 mating types paired with each isolate and incubated in the dark for approximately 1 week and the zone of hyphal interface assessed under a standard light microscope for typical amphigynous oospores. Mefenoxam sensitivity was measured as the percentage growth of an isolate on amended media (100ppm mefenoxam) compared to growth on control media, as previously described [8]. Release of swimming zoospores from a store-bought infected cucumber fruit was induced after incubation at ambient outdoor temperatures (August 3 to 7, 2017, Knoxville, Tennessee, US). The whole fruit was submerged in 500ml of sterile water and incubated for one hour at room temperature. The resulting swimming zoospores were induced to encyst by enclosing the liquid in a bottle and shaking vigorously. The number of swimming spores was estimated using a standard light microscope by averaging 40 hemocytometer counts of 10ul aliquots of encysted zoospores.

### DNA, sequencing, analysis and data availability

Isolates were grown in V8 juice broth and the resulting mycelium was freeze dried and powdered and genomic DNA extracted as described previously [24]. The genomic DNA was sheared using a sonication device and PCR-free libraries constructed as previously described and sequenced on an Illumina HiSeq device running a 2x150 paired-end configuration [23]. The raw data was processed using CLC Genomics Workbench version 9 (Qiagen, US) and trimmed for quality, mapped to the *P*. *capsici* ordered (or unordered) genome at default map settings and heterozygous allele frequencies assigned requiring either >20X coverage (single zoospore progeny) or >10X coverage (additional 33 isolates) and histograms of the major and minor allele frequencies constructed as previously described [24]. Heterozygotes were called between 10% and 90% allele frequency. Histograms were used to plot the allele frequencies for both the major and minor alleles for the non-ordered scaffolds and the linkage-group-ordered scaffolds. Ploidy (chromosomal copy number) was assessed with density plots of coverage per chromosome generated in R with ggplot2. The allele frequencies across a chromosome were plotted using Microsoft Excel. For the single-zoospore progeny, the total number of sequences was >50M for each isolate and the number of polymorphic SNP loci was 195,482.

## Results

### Isolates, sectoring and zoospore production from a naturally infected cucumber

**Fig 1:**
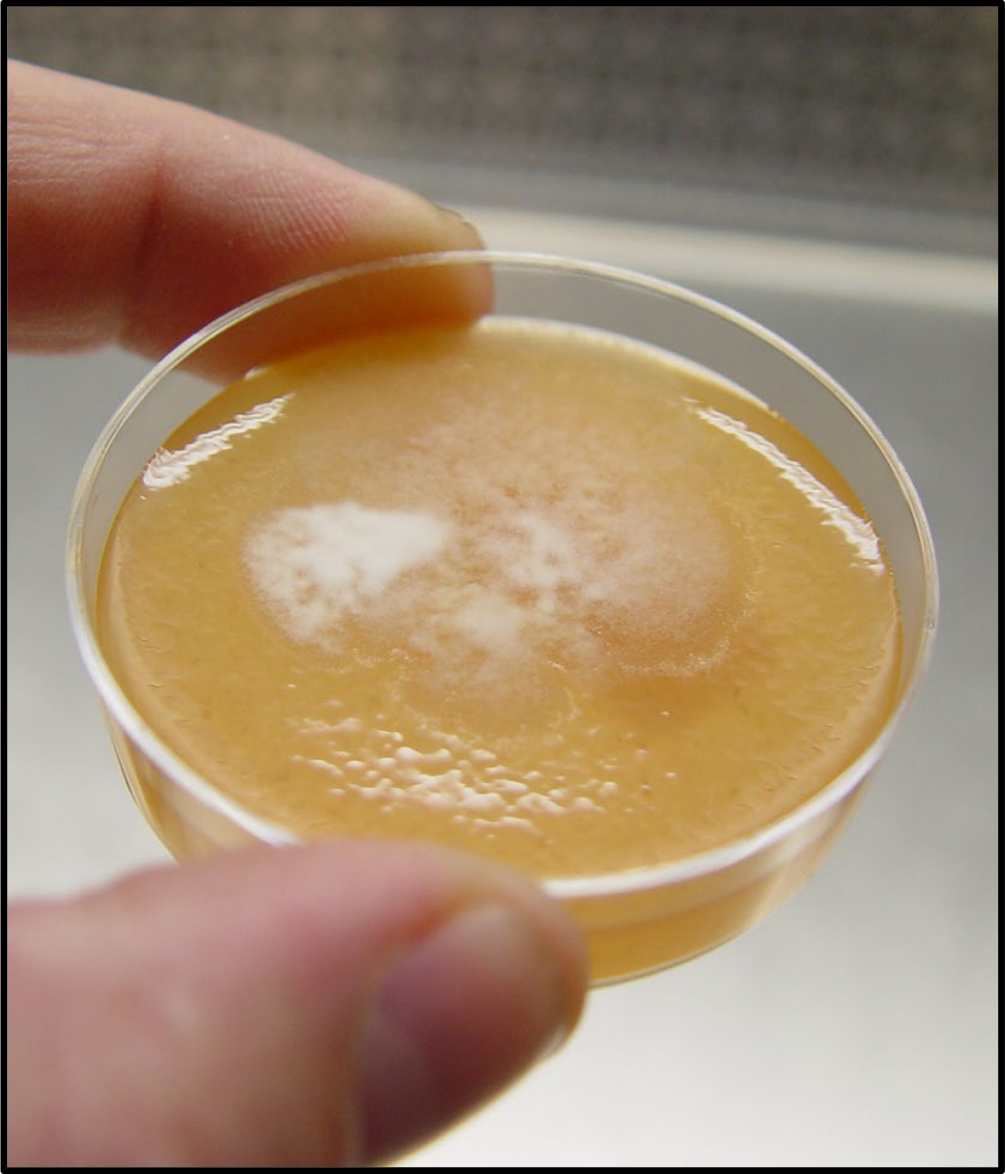
Example of sectoring in a single-zoospore derived isolate of *Phytophthora capsici* used in this study.

**Fig 2:**
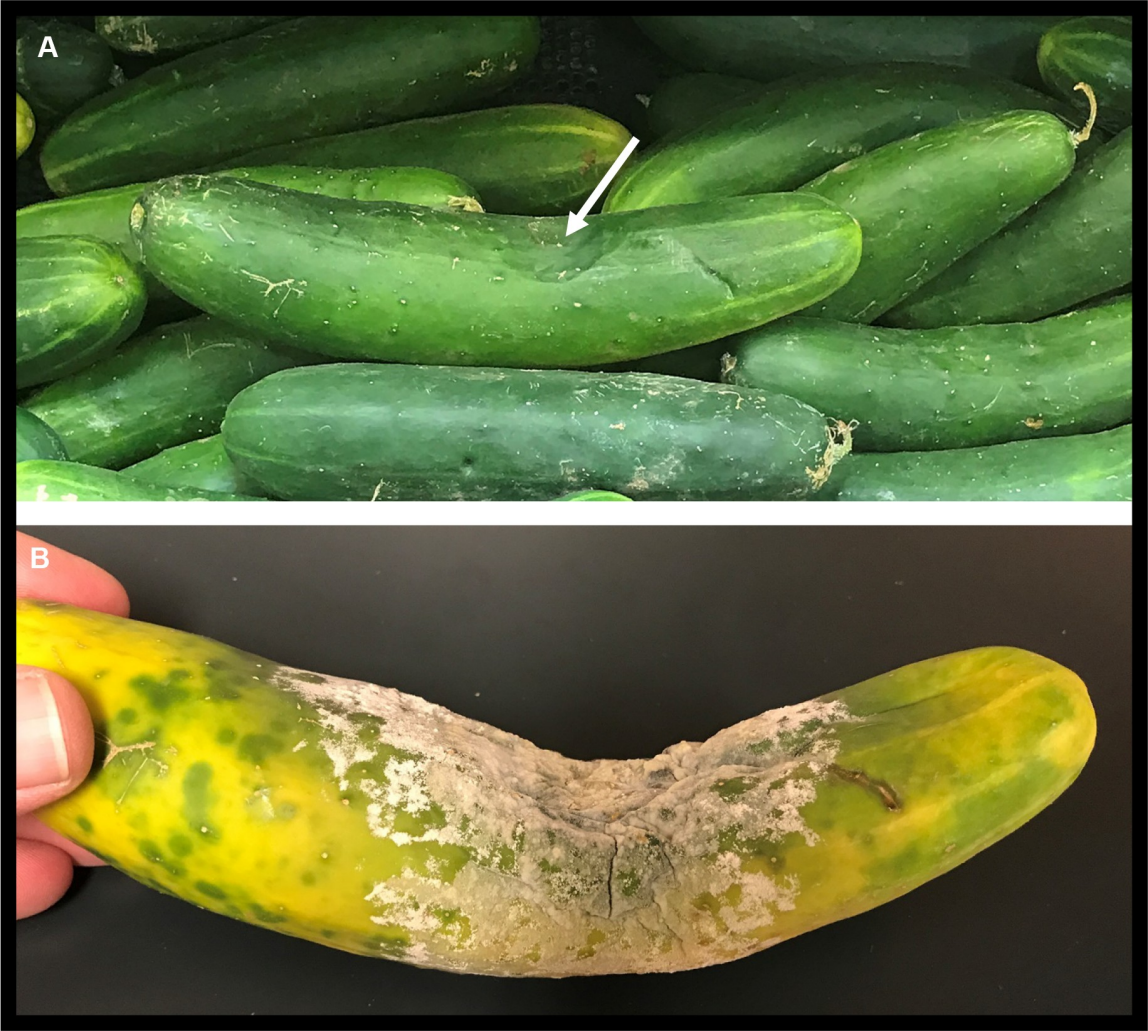
Cucumber fruit recovered August 3, 2017 from major supermarket chain in Knoxville, Tennessee (origin unknown). A, white arrow designates typical sunken, firm lesion produced by infection with *P*. *capsici*. B, the same fruit producing millions of asexual sporangia-spores after three days incubation under ambient (outdoor) conditions (K. Lamour, Knoxville, TN, Aug 3–6).

The isolates described here were collected, and experiments conducted, over the course of approximately 10 years and includes genome resequencing for 38 isolates of *P*. *capsici* or near-relatives (e.g. *P*. *tropicalis*) (S1 Table). Of these, 26 are isolates recovered from naturally infected plants at locations in the US, South America, Europe and China. Three (LT1021, LT1422 and LT1534) are oospore progeny produced via backcrossing to reduce heterozygosity for genome sequencing. One isolate (LT9378) is a sub-culture, stored independently for two years, of the isolate LT1534 which was used for the construction of the reference genome. Four isolates were sub-cultured from obvious sectoring (altered growth rate or pattern) that occurred while field isolate LT9107 was growing on agar media. And finally, four isolates are single-zoospore copies of field isolate LT9136, recovered from pepper in China in 2010. Sequences for all isolates described in this report are deposited in the NCBI under PRJNA386483. In addition to the re-sequenced isolates, 384 single zoospore isolates from three A1 and three A2 field isolates were tested for their inheritance of mating type and 254 single zoospore isolates (recovered from six field isolates) were tested for their inheritance of mefenoxam sensitivity (Tables 1 and 2). Fig 1 shows an example of a single-spore derived isolate of *P*. *capsici* growing on standard V8 agar with obvious sectoring emanating from the point of inoculation. Immersion of a freshly sporulating cucumber, obtained from a local market, produced >100 million bi-flagellate swimming zoospores (Fig 2).

**Table 1:** Summary of mating type switch in zoospore progenies of *P*. *capsici* from the USA and China.

Isolate	Origin	Mating type	No. of zoospore progenies	No. of zoospore progenies		
				A1	A2	A1/A2 ^a^
LT263	USA	A2	143	24	12	107
LT9104	China	A2	50	1	10	39
LT9136	China	A2	48	1	40	7
LT9194	China	A1	46	46	0	0
LT9397	China	A1	47	47	0	0
LT9400	China	A1	47	47	0	0

^a^ Isolates that produce oospores when crossed with A1 and A2 testers are self-fertile.

**Table 2:** Summary of *Phytophthora capsici* parental isolates used for monitoring mefenoxam sensitivity in zoospore progeny.

Isolate	Mating type	Year	Origin	Growth rate ^a^	Mefenoxam sensitivity ^b^	Note
LT9397	A1	2007	China	0.28	S	Field
LT9398	A1	2011	China	0.74	IR	LT9397 sectoring
LT9400	A1	2007	China	0.09	S	Field
LT9402	A1	2011	China	0.41	IR	LT9400 sectoring
LT10287	A1	2012	USA	0.64	IR	Field
LT10290	A2	2012	USA	0.84	IR	Field

^a^ Growth rate = average colony diameter on medium amended with 100 ppm mefenoxam / average colony diameter on mefenoxam non-amended medium.

^b^ Mefenoxam sensitivity was divided by the criteria as previously described (Kurt Lamour 2000). S = sensitive, IR = intermediate resistant.

### Asexual inheritance of heterozygous loci

**Fig 3:**
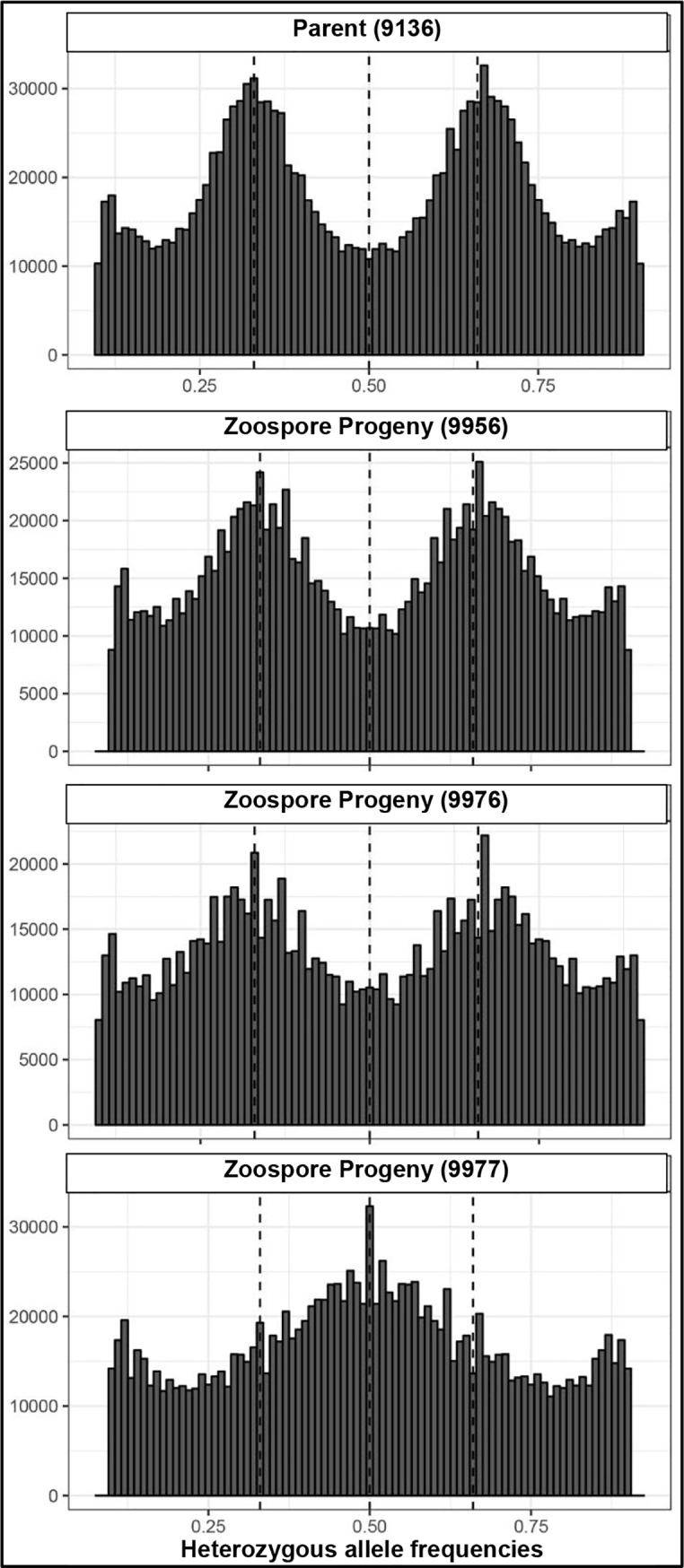
Histograms showing genome-wide heterozygous allele frequencies for parent and zoospore progeny of the vegetable pathogen, *P*. *capsici*, based on mapping to the unordered 917 scaffolds of the *P*. *capsici* reference genome. Dotted lines indicate 25, 50 and 75% allele frequencies. The dominant trends visible here do not accurately reflect individual chromosome copy number or provide an accurate summary of allele frequencies as they occur within the individual chromosomes.

**Fig 4:**
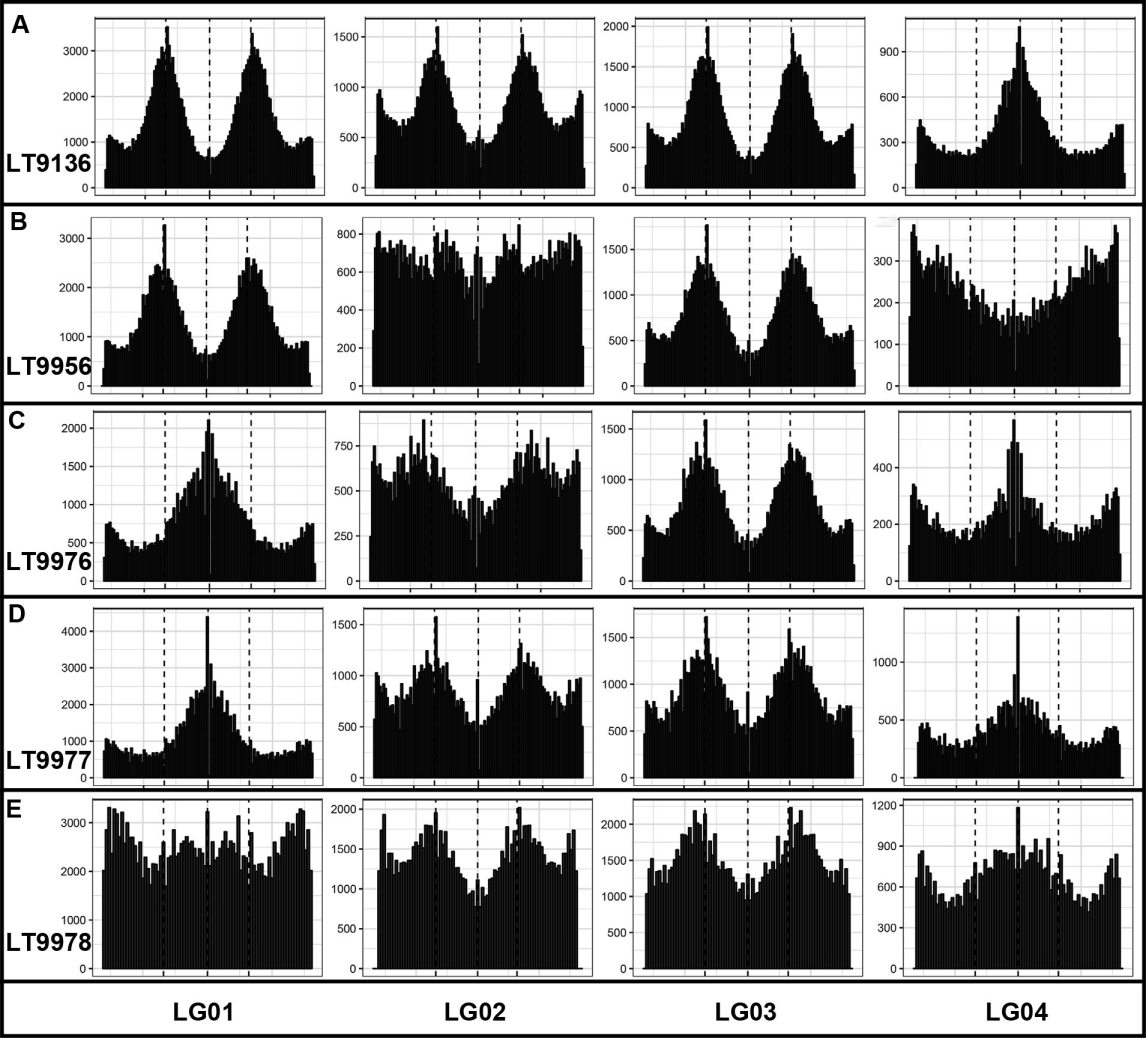
**Linkage Group (LG) allele frequency histograms for a field isolate of *Phytophthora capsici* (A) and four mitotic zoospore progenies (B to E) for LG’s 1 to 5.** The y-axis denotes the number of markers and the x-axis denotes heterozygous allele frequencies from 0 to 1 with dotted vertical lines at 33, 50 and 66%.

**Fig 5:**
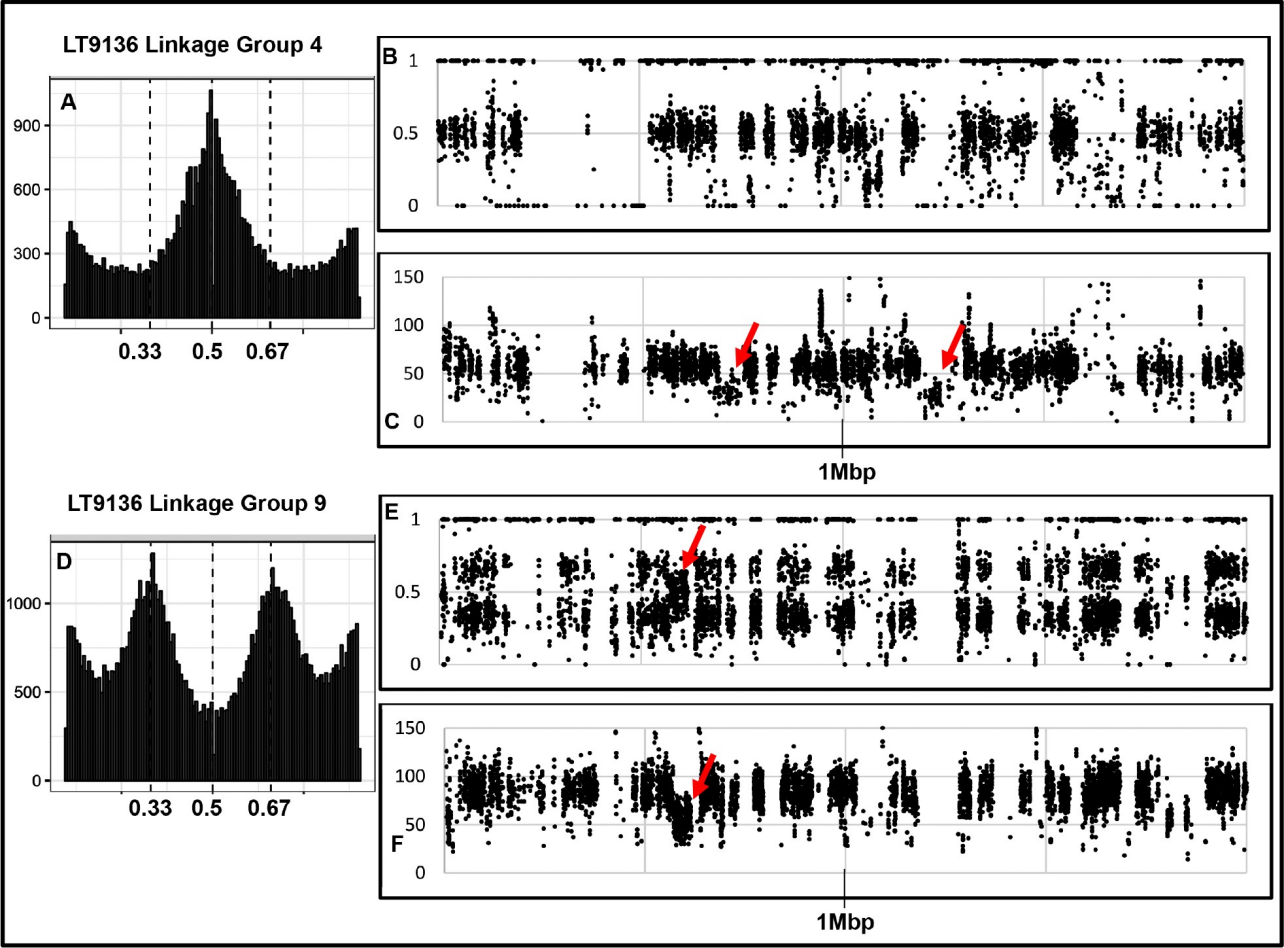
Heterozygous allele frequencies and sequence coverage for *Phytophthora capsici* isolate LT9136 within linkage groups 4 and 9. Histograms (A, D) plot the major and minor allele frequencies. B and E plot allele frequencies across 2Mbp for each linkage group and C and E plot the sequence depth across the same region. Notice most allele frequencies reflect copy number except the regions indicated by red arrows. In linkage group 4 (diploid), copy number at two sites appear haploid and the allele frequencies are fixed at 0 or 1. In linkage group 9 (triploid) the copy number and allele frequency appear diploid.

**Fig 6:**
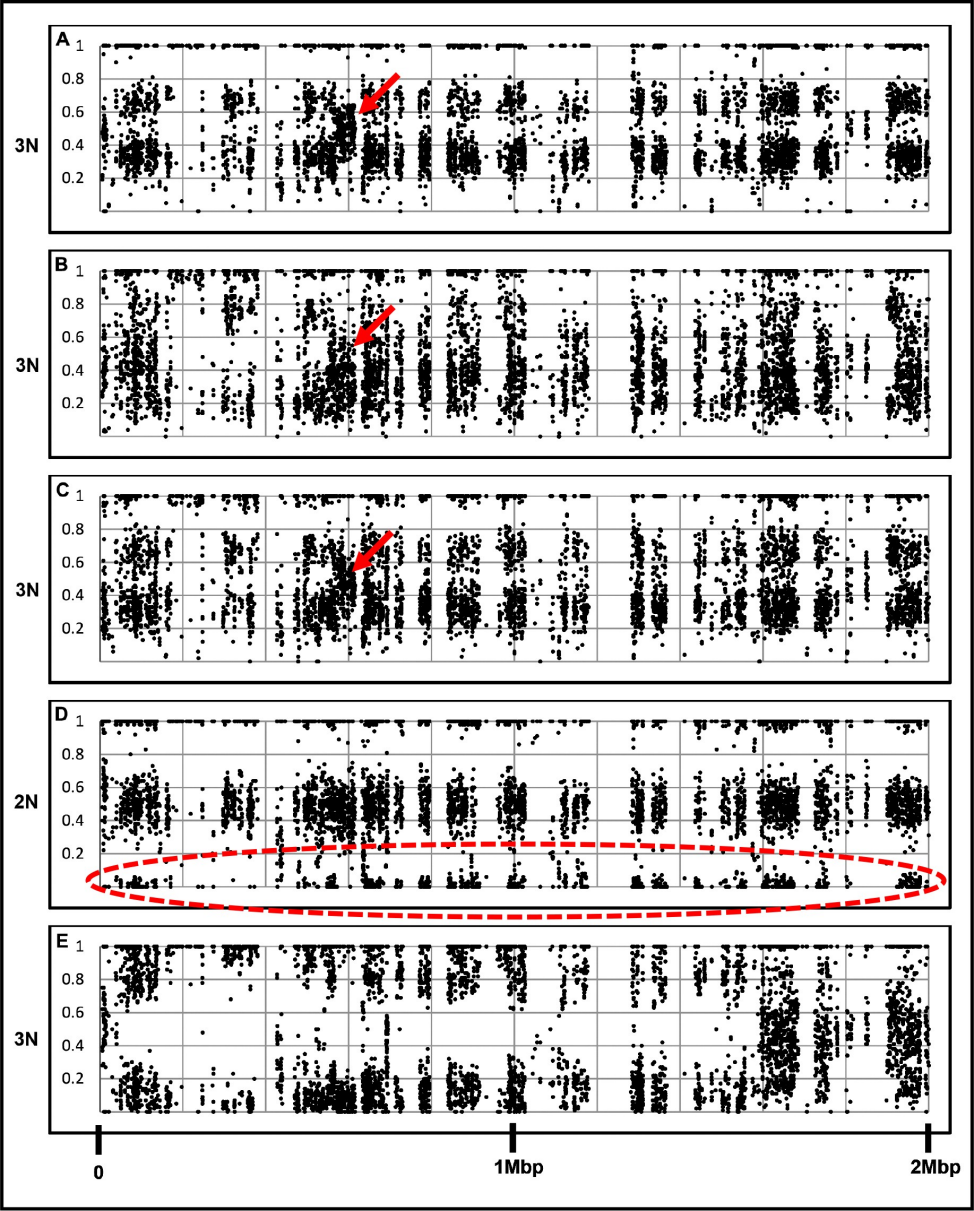
**Plots of heterozygous allele frequencies for a parent (A) and four single-zoospore progenies (B-E) across 2Mbp of linkage group 9.** Ploidy (based on sequencing depth) is denoted on the left as either 2 or 3N. For isolates A to D the allele frequencies mainly correspond with ploidy (note exceptions at red arrows). Isolate D has switched to the diploid state with fixation of many alleles (alleles fixed for absence circled in red). Isolate E exhibits allele frequencies that differ dramatically from the parental isolate and based on the expectations for ploidy of 3N.

**Fig 7:**
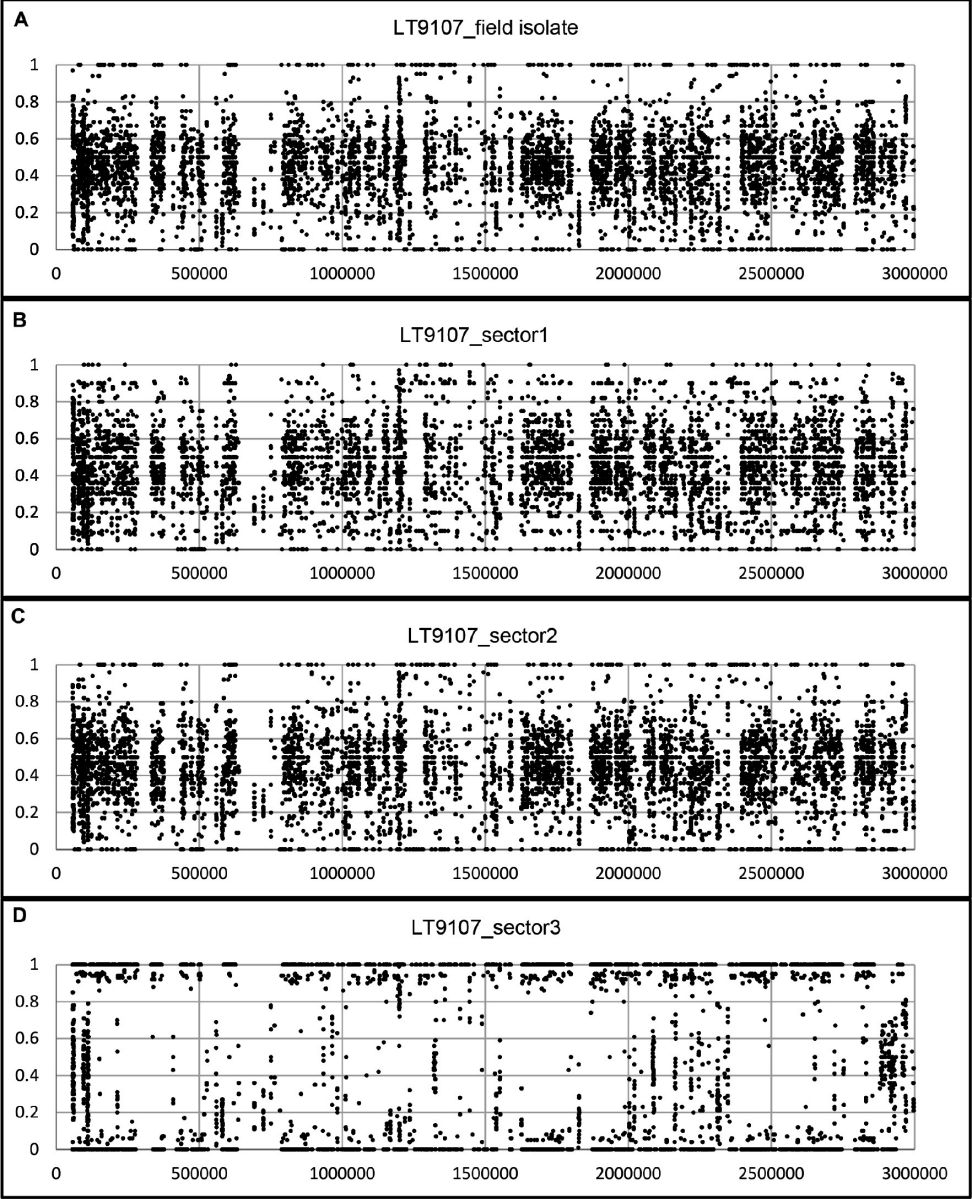
Heterozygous allele frequencies across 3Mbp of linkage group 8 for a single-zoospore derived isolate of *Phytophthora capsici* recovered from pepper in China and subcultures from mycelium sectoring on agar media. Sequencing depth indicates isolates are diploid. Note the wide distribution of allele frequencies for A-C and fixation of most of the alleles in D (copy neutral loss of heterozygosity).

One of our most perplexing findings, based on the genome re-sequencing, was that heterozygous allele frequencies did not correspond directly with chromosome copy number (ploidy). In addition, visualizing heterozygous allele frequencies, en masse, can lead to erroneous conclusions about a genome’s ploidy. For example, the composite allele frequencies, using the 917 unordered *P*. *capsici* scaffolds, suggest the genomes for the field-isolate, LT9136, and the four single-zoospore progenies are primarily diploid or triploid with alternate allele frequencies creating roughly modal distributions around 50% (apparently diploid) or 33 and 66% (apparently triploid) (Fig 3). The same data, visualized by chromosome (based on the genetic map-ordered scaffolds), reveals the allelic complement of the larger chromosomes can obscure the allele distributions for the smaller chromosomes (Fig 4, S4 Fig). Plots of the heterozygous allele frequencies and sequence depth for field isolate LT9136 and zoospore progeny, across individual chromosomes, reveal intra-genomic variation of chromosome copy number varies between 2N and 3N. Heterozygous allele frequencies can range anywhere from fixation (0 or 100%) to essentially any allele frequency in between—often in combinations entirely different from the parent (S1–S4 Figs). Plotting the sequence depth and the heterozygous allele frequencies across the chromosomes for single-zoospore isolates and for isolates recovered from sectoring on agar media indicates allele frequencies can be a poor measure of a chromosome’s copy number (Figs 5–7 and S1–S4 Figs). The chromosome-specific allele frequencies and coverage plots for 33 isolates of *P*. *capsici* (or near relatives such as *P*. *tropicalis* and the provisional species ‘P. subnubulis’) indicate at least 20% of these isolates carry heterogenous heterozygous allele frequencies and/or intra-genomic variation in chromosome copy number (S1 Table, S1 and S2 Figs). Interestingly, isolate LT1534, which was used to produce the *P*. *capsici* reference genome was sequenced on two different occasions (approximately 2 years apart and named LT9378 for resequencing) and in each case exhibits different patterns of intra-genomic allele frequency (S1 Table, S1 Fig).

In addition to our analysis of single-zoospore isolates and field isolates we also sequenced isolates recovered from fast and slow growing sectors of a single-zoospore derived field isolate from China (LT9107). Although the chromosomal copy numbers appear primarily diploid, the allele frequencies for about 3Mbp of linkage group 8 have primarily gone to fixation (copy neutral loss of heterozygosity) (Fig 7, S1 Table, S1 Fig).

### Stability of mating type and sensitivity to mefenoxam

Analysis of 384 single-zoospore isolates, derived from three A1 and three A2 mating type isolates, indicate mating type is highly unstable for A2 isolates. All 140 of the A1-derived isolates remained true to the A1 mating type. For the 241 A2-derived isolates, only 26% remained A2, 11% switched to A1 and 63% became self-fertile (Table 1).

**Fig 8:**
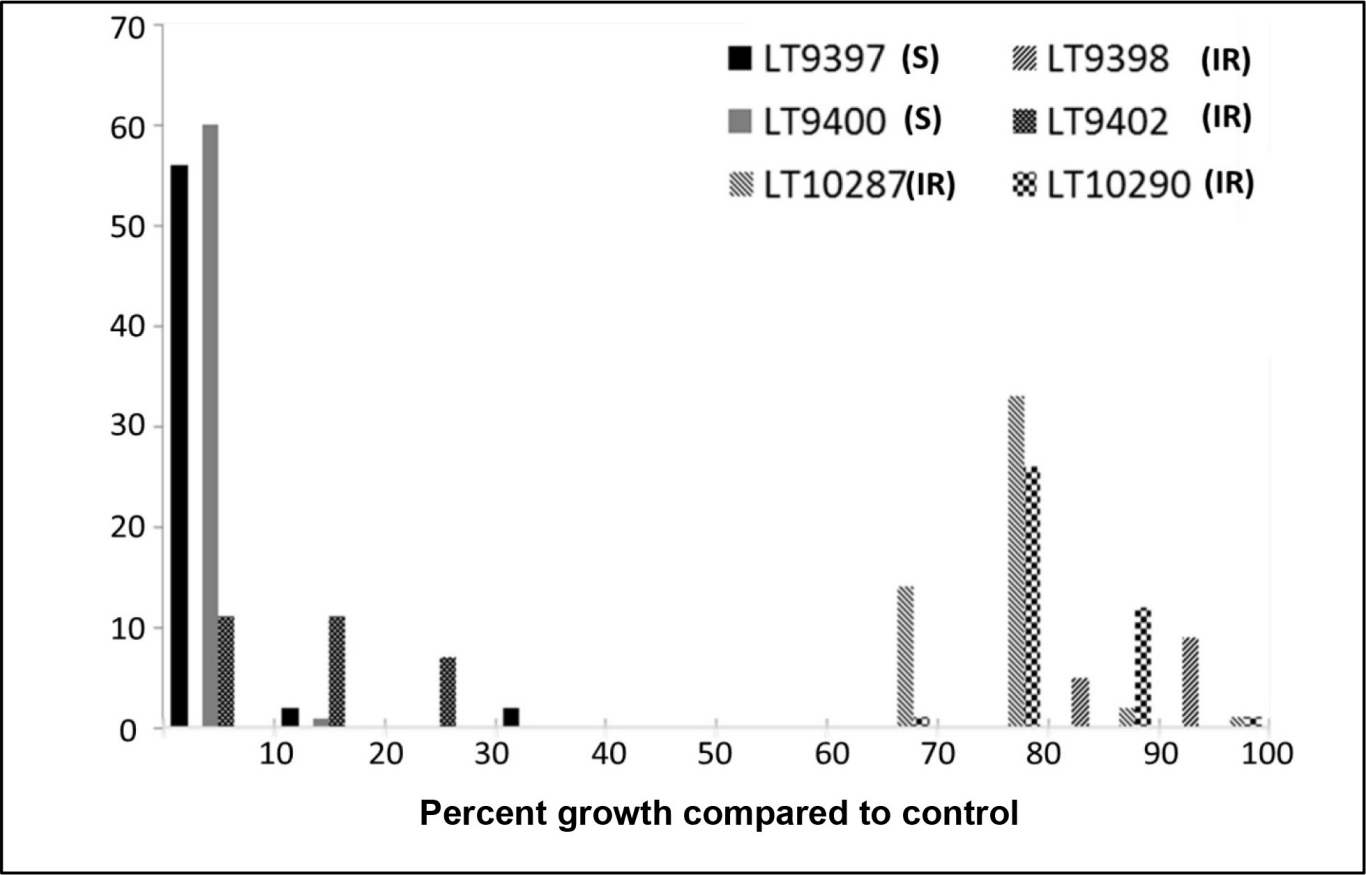
Response of 254 *Phytophthora capsici* single zoospore progeny from six field isolates to the oomycete-toxic chemical, mefenoxam. The six parental field isolates are listed in the upper right and denoted with various fill patterns. Parental isolates are either sensitive (S) to mefenoxam where their ability to grow on mefenoxam media is low compared to growth on un-amended control media or intermediately resistant (IR) where they can grow at roughly half their normal rate of growth on amended media. The progeny, charted as a histogram, ranges from fully sensitive to fully resistant.

Our tests of 254 single-zoospore isolates from sensitive and intermediately sensitive field isolates revealed that progeny from sensitive isolates almost always remained sensitive whereas progeny from intermediately sensitive isolates can range from intermediate to fully resistant– most likely driven by the increased copy number of the resistance allele that can occur via the process of DEA (Table 2, Fig 8).

## Discussion

For most eukaryotic organisms, the allele frequencies of unique (single copy) heterozygous SNP loci reflect ploidy. This is a crucial correlation as it allows the development of genetic linkage maps and genome wide association studies and the allele frequencies are key tools to track survival and spread in natural populations. The ability to make, store and manipulate clonal copies of individual isolates is important to all aspects of controlled (replicated) research. Here we present genome-wide SNP data describing an unprecedented level of asexual (mitotic) intra-genomic diversity for the broad host range vegetable pathogen *P*. *capsici* and dub this phenomenon Dynamic Extreme Aneuploidy (DEA). We also report rapid changes in mating type and drug resistance in single-spore isolates showing how DEA may drive important evolutionary events and hinder the progress of routine research. Recent investigations of field populations of *P*. *capsici* and the closely related *P*. *colocasiae* used genome-wide heterozygous allele frequencies to conclude individual isolates can carry a diverse complement of chromosomes varying between 2N to 6N (or more). Considering our current data, these conclusions are likely overestimates as we show that heterozygous allele frequencies are unlikely to accurately reflect chromosomal copy number.

Like many plant pathogens, *P*. *capsici* produces a massive number of spores to achieve evolutionary success. Fig 2 highlights the speed and magnitude of spore production for *P*. *capsici*. If this fruit were infected by one or a few isolates of *P*. *capsici*, we expect many copies of one or a few genomes to be released into the environment. Instead, our data indicate the number of isolates colonizing a plant may be limited but that dramatic changes can occur during sporulation and asexual mycelial growth to produce a diverse array of genomes bearing little fidelity to isolate(s) originally causing infection. Zoospores can swim for days, are negatively geotropic (swim up) and chemotactic (swim towards plant exudates) [1]. If *P*. *capsici* adhered strictly to the rules of mitosis, each zoospore should be an identical copy of the parent genome and contain the same dosage (ploidy) across all chromosomes and the allele frequencies at all heterozygous loci should reflect the overall chromosome copy number. Our sequence data on parental and zoospore-derived isolates shows that this is not always the case and the overall restructuring of the genome can be dramatic and, potentially, may include a huge variety of ploidy and allele frequency combinations. Population studies using single-zoospore, hyphal-tip and/or infected-plant derived genomic DNA support our *in vitro* observations [13, 23]. Field populations, especially during the explosive asexual phase of an epidemic, contain many individuals with slightly differing multi-locus SNP genotypes, consistent with the extreme asexual plasticity reported here, and inconsistent with the expectations for meiosis as the sole driver of genetic diversity [12, 13].

In North America and Mexico, *P*. *capsici* often undergoes meiosis through the interaction of an A1 and A2 mating type to produce thick-walled, long-lived sexual dormancy spores (oospores) [25–27]. Yet, newly introduced populations, even those emanating from a small focus of initial infection (e.g. a single infected plant with clonal isolates), often appear to accomplish sexual outcrossing within the first year of an epidemic. Our data indicates the A2 mating type is highly plastic and can readily switch to either the A1 mating type or produce sexual spores without interacting with any other isolate (homothallism, or self-fertilization), presumably by the interaction of some of the original A2 genotypes with those that switched to A1. Recently, genetic analysis of a bi-parental inbreeding field population of *P*. *capsici* revealed the A2 mating type requires elevated heterozygosity across a distinct mating-type region and DEA may play an important role in the development of sexually-active populations, even where the introductory event was limited to a single A2 mating type.

Another example of how plasticity of chromosome number can impact *P*. *capsici’s* fitness potential is in its ability to rapidly acquire drug (mefenoxam) resistance. Mefenoxam is a commonly used oomycete-toxic compound with a site-specific mode of action and resistance often occurs rapidly [8, 9, 11, 28]. In *P*. *capsici*, resistance can be mediated by co-dominant loci of major effect, although the exact mutations are unknown [8, 28]. In Michigan, close monitoring of commercial field populations exposed to mefenoxam revealed rapid acquisition of full resistance without a concomitant genetic bottleneck or the requisite dormancy periods to allow sexual outcrossing to produce progeny homozygous for the resistance allele(s) [8, 9, 28]. Fully resistant populations appeared to be as genetically diverse as genetically isolated sensitive populations suggesting the resistance allele somehow bypassed the two rounds of outcrossing (and mandatory dormancy) needed for meiosis to produce homozygous (fully resistant) individuals. Our data, overall, illustrate how a region of the *P*. *capsici* genome harboring a resistance allele (or any advantageous allele) can be increased in frequency (or fixed)–simply through the process of asexual sporulation and/or mitotic growth, coupled with chromosome copy number changes and loss of heterozygosity.

The extreme plasticity of Phytophthora is well-known and although our data does not elucidate the processes driving DEA; knowing it exists is the first step towards understanding this novel process. *Phytophthora capsici* produces millions of spores on infected plants and the opportunities for adaptive and nearly instantaneous evolution are impressive. The obverse of this decidedly positive situation for *P*. *capsici* (and likely other oomycetes) is the challenge imposed upon routine genetic analyses and the construction of stable community resources. Although this work primarily describes DEA as it manifests during zoosporogenesis, we’ve observed many instances where significant changes in pathogenesis and mating type occur spontaneously within individual cultures (without any obvious intervening zoospore formation) (Kurt Lamour, unpublished data). Clearly, more research is needed to better understand the processes driving the organization of the Phytophthora genome during asexual reproduction and to further explore the spectrum of evolutionary implications.

## Supporting information

S1 Fig Histograms showing allele frequencies across 18 linkage group for 33 isolates of *Phytophthora capsici* or near relatives. All isolates were sequenced using PCR-free library preparations following random disruption using a sonication device on an Illumina HiSeq device running 100bp single end or 2x150 paired-end sequencing and genotypes called for sites with >15X coverage.
(PDF)

S2 Fig Sequence coverage density plots for 33 isolates of *Phytophthora capsici* or near relatives. (PDF)

S3 Fig Sequence coverage of a parent and four single zoospore progenies for the 10 largest linkage groups of *Phytophthora capsici*. (PDF)

S4 Fig Allele frequency histograms for parent and 4 zoospore progeny of *Phytophthora capsici* for linkage groups 1 to 10. (PDF)

S1 Table Summary data for additional sequenced Phytophthora capsici or close relatives. (XLSX)
