# Supplementary material for: Dynamic Extreme Aneuploidy (DEA) in the vegetable pathogen Phytophthora capsici and the potential for rapid asexual evolution
Source: PLoS One. 2020 Jan 7;15(1):e0227250. doi: 10.1371/journal.pone.0227250 (PMC6946123; doi:10.1371/journal.pone.0227250)
Supplement: S1 Fig — All isolates were sequenced using PCR-free library preparations following random disruption using a sonication device on an Illumina HiSeq device running 100bp single end or 2x150 paired-end sequencing and genotypes called for sites with >15X coverage. (PDF) [file pone.0227250.s001.pdf]

Phytophthora capsici Linkage group 01 allele frequencies

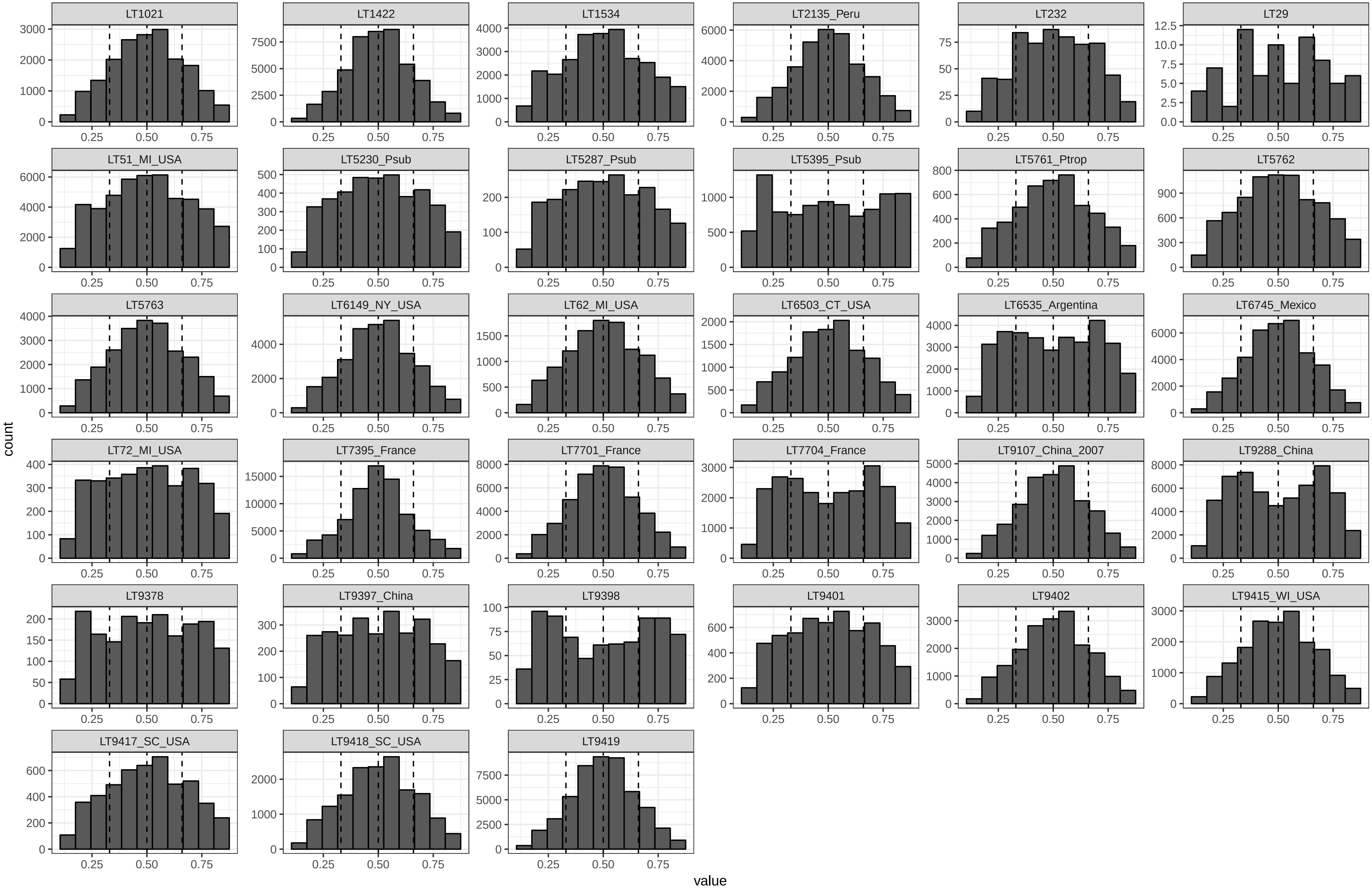

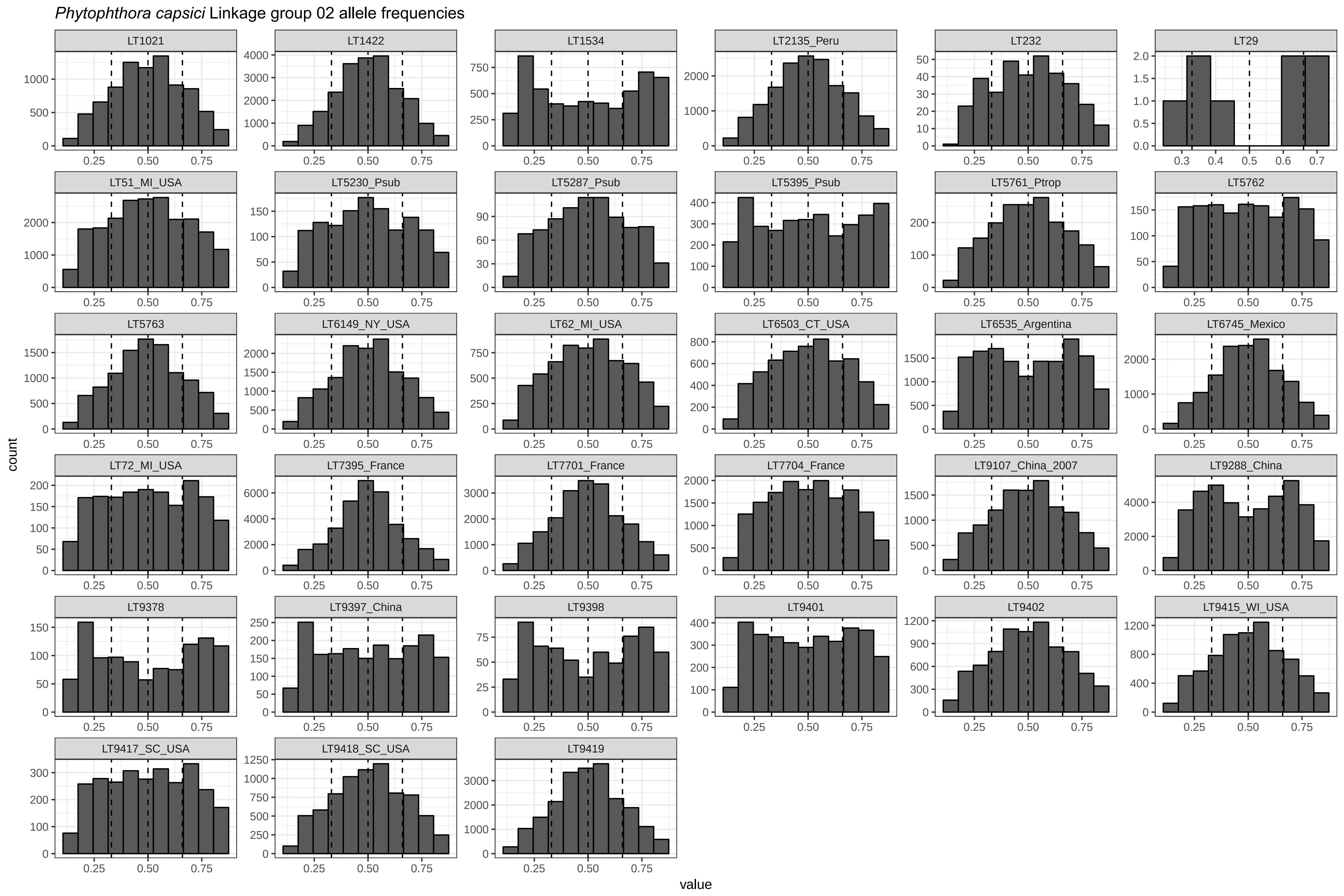

Phytophthora capsici Linkage group 02 allele frequencies
LT1021
LT1422
LT1534
LT2135_Peru
LT232
LT29
LT51_MI_USA
LT5230_Psub
LT5287_Psub
LT5395_Psub
LT5761_Ptrop
LT5762
LT5763
LT6149_NY_USA
LT62_MI_USA
LT6503_CT_USA
LT6535_Argentina
LT6745_Mexico
LT72_MI_USA
LT7395_France
LT7701_France
LT7704_France
LT9107_China_2007
LT9288_China
LT9378
LT9397_China
LT9398
LT9401
LT9402
LT9415_WI_USA
LT9417_SC_USA
LT9418_SC_USA
LT9419
count
value

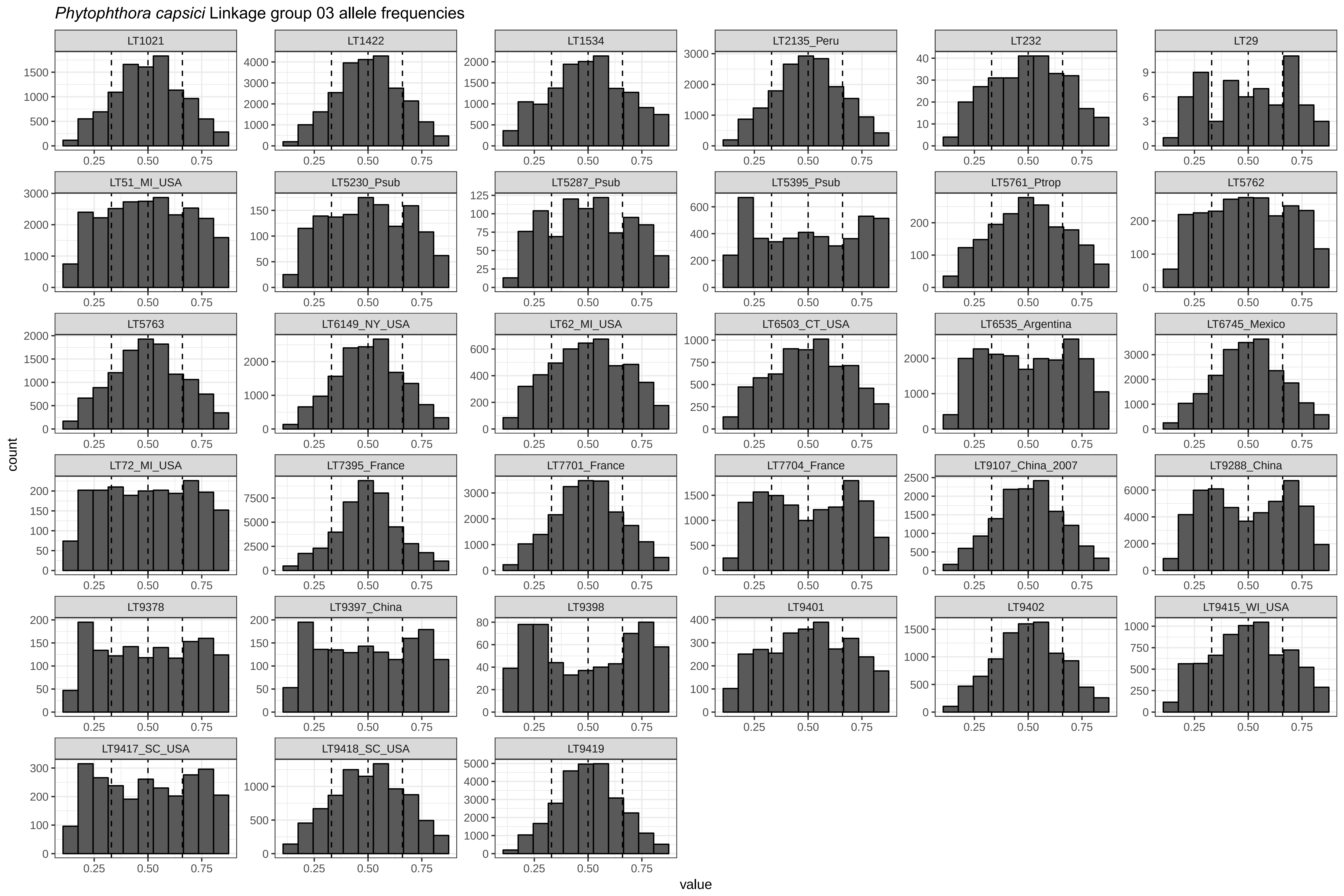
Phytophthora capsici Linkage group 03 allele frequencies
LT1021
LT1422
LT1534
LT2135_Peru
LT232
LT29
LT51_MI_USA
LT5230_Psub
LT5287_Psub
LT5395_Psub
LT5761_Ptrop
LT5762
LT5763
LT6149_NY_USA
LT62_MI_USA
LT6503_CT_USA
LT6535_Argentina
LT6745_Mexico
LT72_MI_USA
LT7395_France
LT7701_France
LT7704_France
LT9107_China_2007
LT9288_China
LT9378
LT9397_China
LT9398
LT9401
LT9402
LT9415_WI_USA
LT9417_SC_USA
LT9418_SC_USA
LT9419
count
value

Phytophthora capsici Linkage group 04 allele frequencies

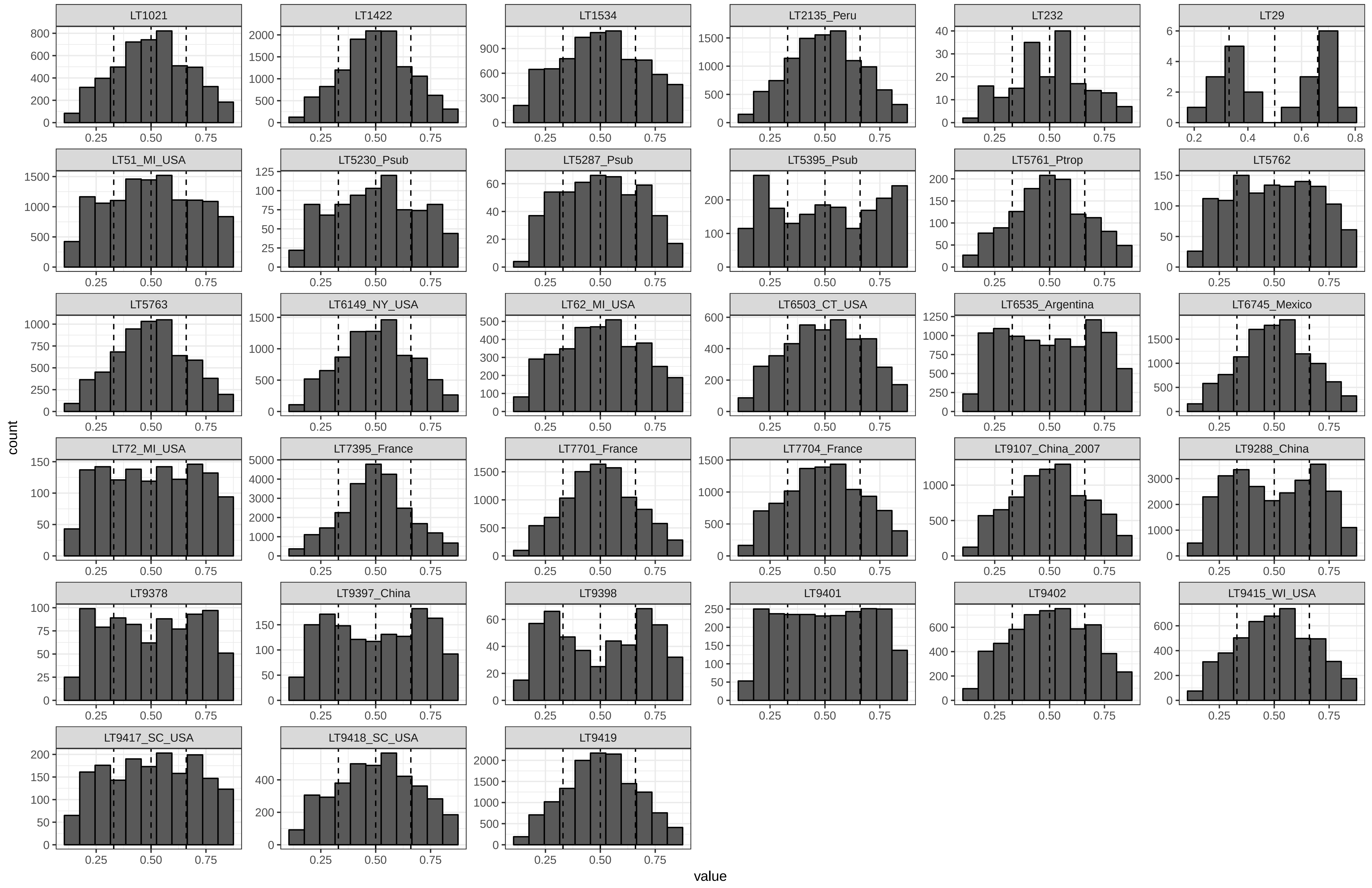

Phytophthora capsici Linkage group 05 allele frequencies

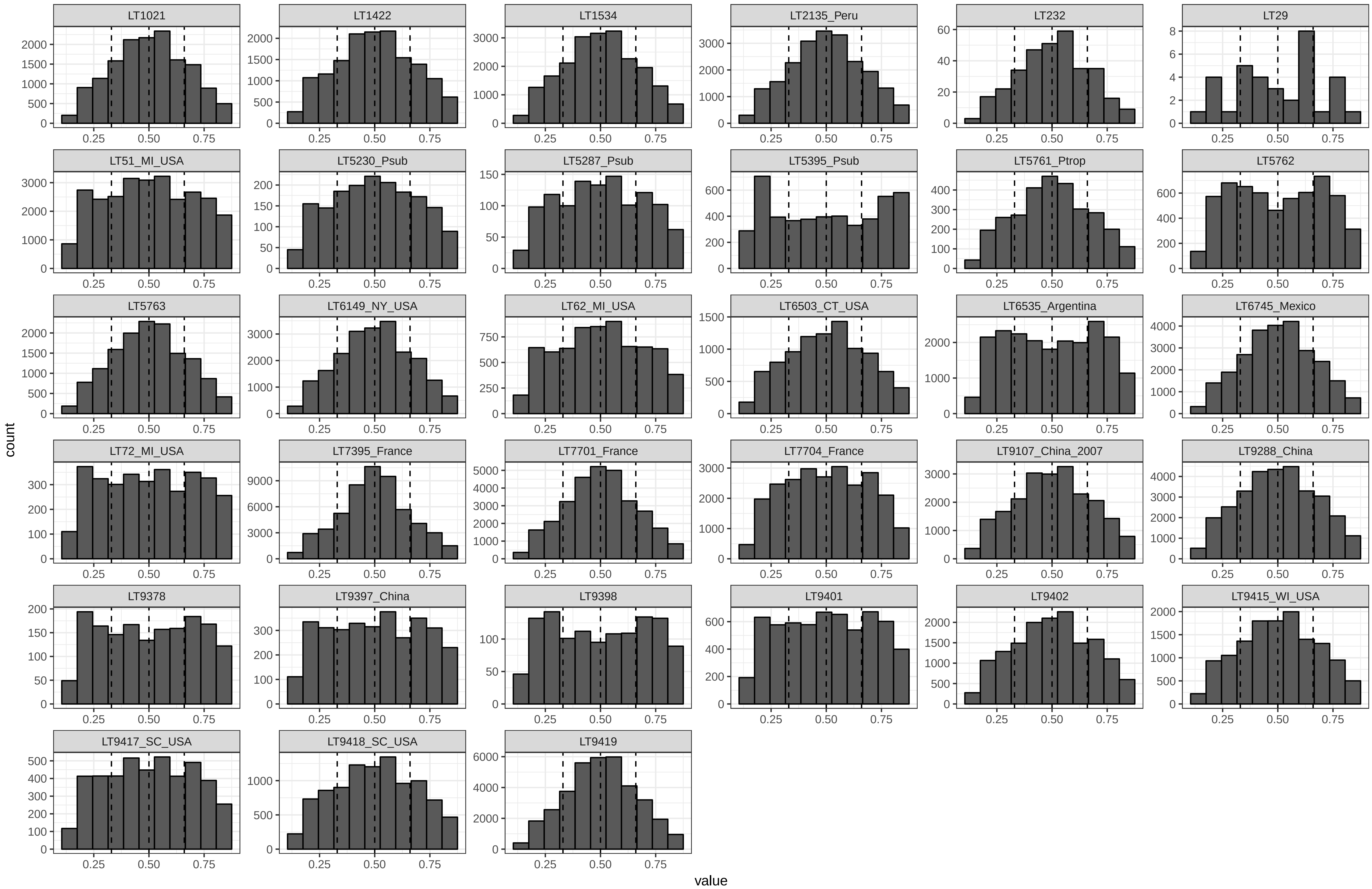

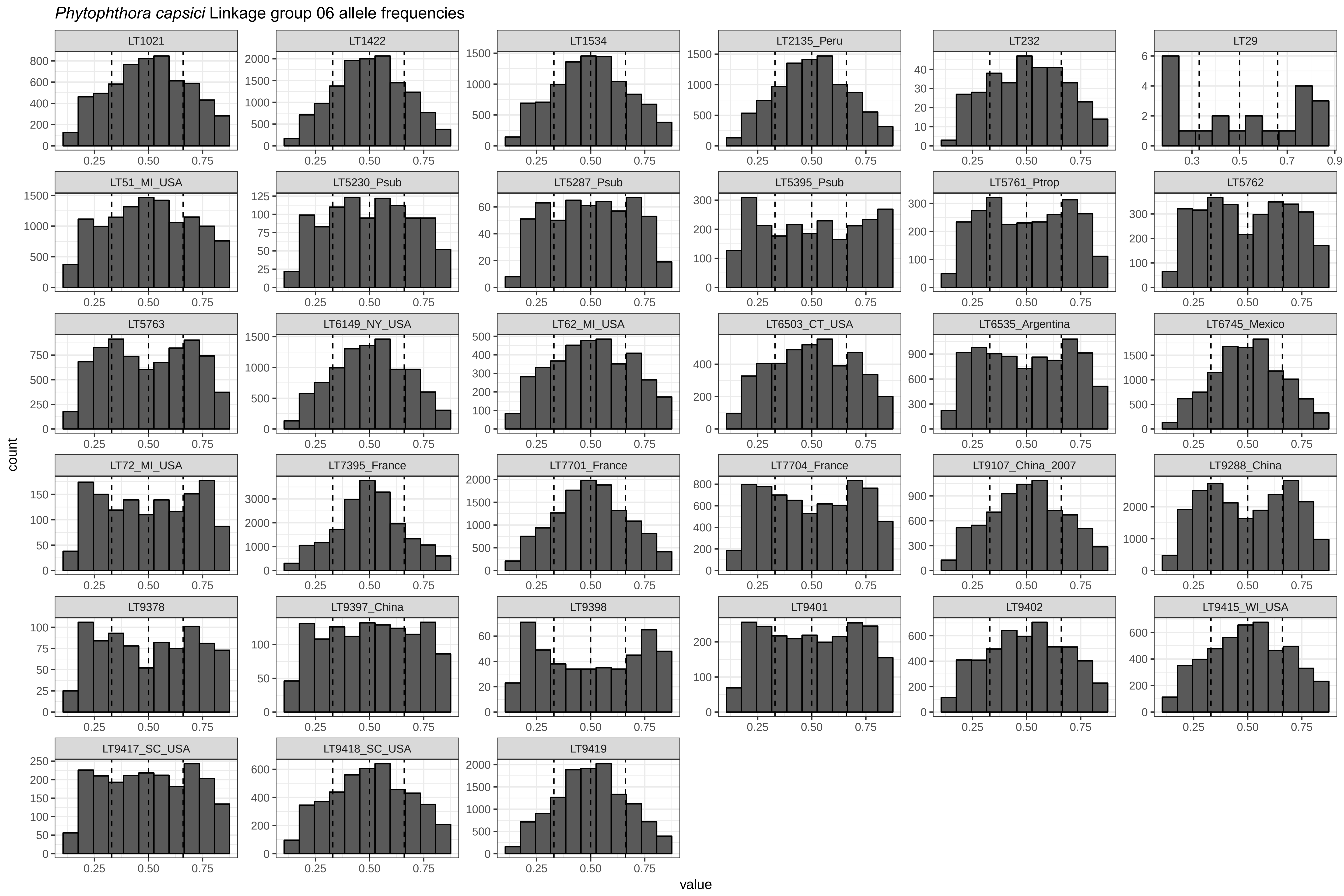

Phytophthora capsici Linkage group 06 allele frequencies
LT1021
LT1422
LT1534
LT2135_Peru
LT232
LT29
LT51_MI_USA
LT5230_Psub
LT5287_Psub
LT5395_Psub
LT5761_Ptrop
LT5762
LT5763
LT6149_NY_USA
LT62_MI_USA
LT6503_CT_USA
LT6535_Argentina
LT6745_Mexico
LT72_MI_USA
LT7395_France
LT7701_France
LT7704_France
LT9107_China_2007
LT9288_China
LT9378
LT9397_China
LT9398
LT9401
LT9402
LT9415_WI_USA
LT9417_SC_USA
LT9418_SC_USA
LT9419
count
value

Phytophthora capsici Linkage group 07 allele frequencies

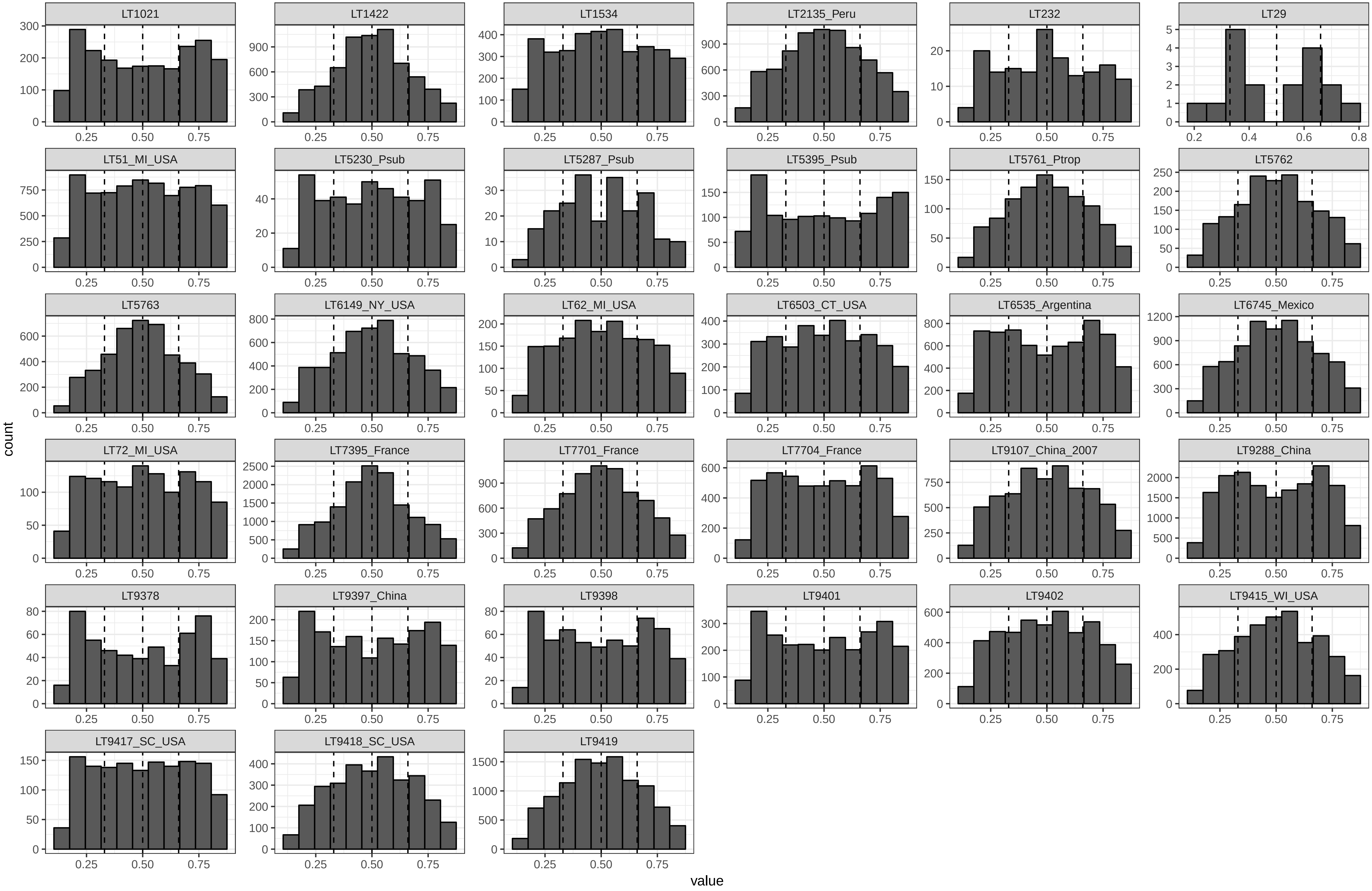

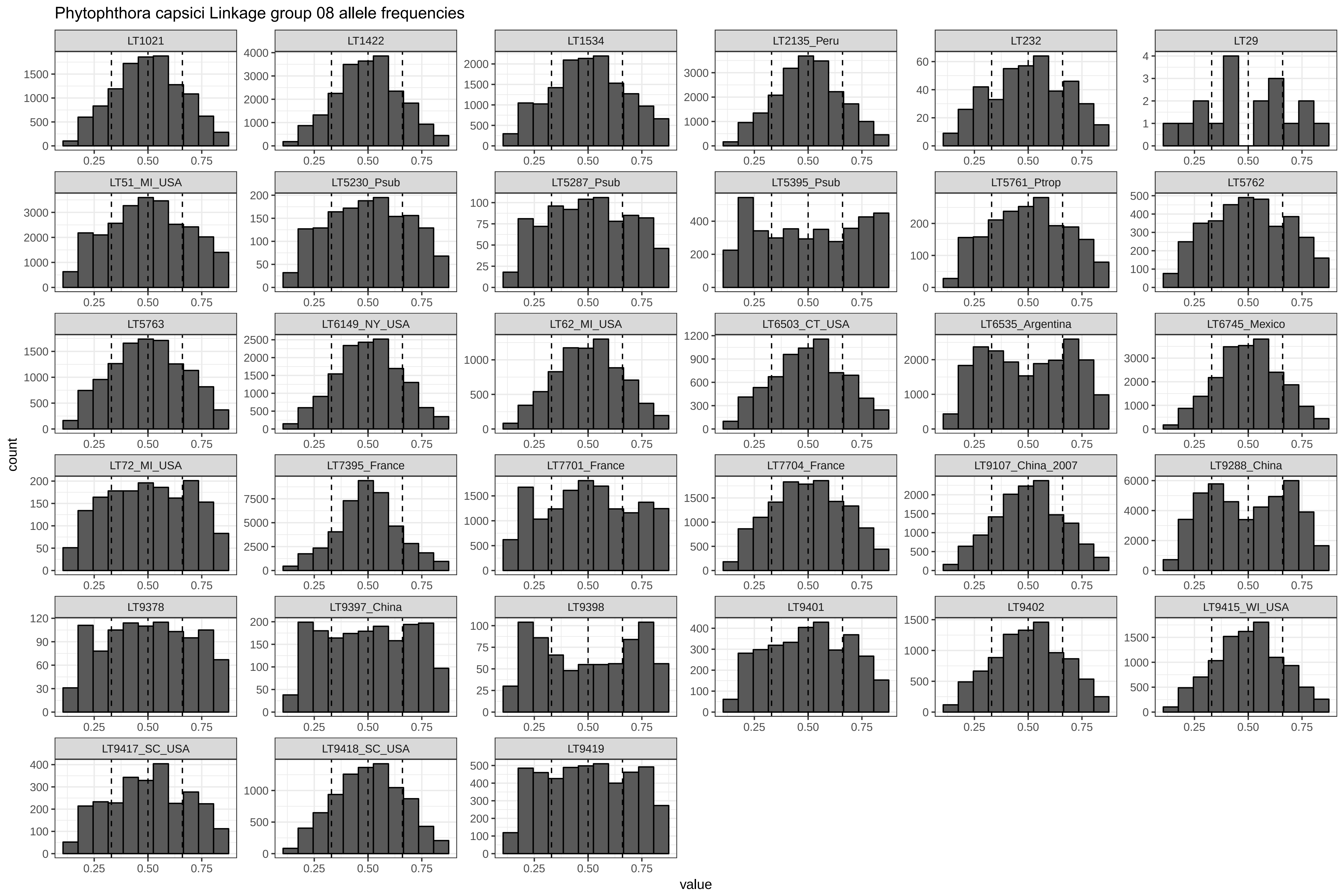

Phytophthora capsici Linkage group 08 allele frequencies
LT1021
LT1422
LT1534
LT2135_Peru
LT232
LT29
LT51_MI_USA
LT5230_Psub
LT5287_Psub
LT5395_Psub
LT5761_Ptrop
LT5762
LT5763
LT6149_NY_USA
LT62_MI_USA
LT6503_CT_USA
LT6535_Argentina
LT6745_Mexico
LT72_MI_USA
LT7395_France
LT7701_France
LT7704_France
LT9107_China_2007
LT9288_China
LT9378
LT9397_China
LT9398
LT9401
LT9402
LT9415_WI_USA
LT9417_SC_USA
LT9418_SC_USA
LT9419
count
value

Phytophthora capsici Linkage group 09 allele frequencies

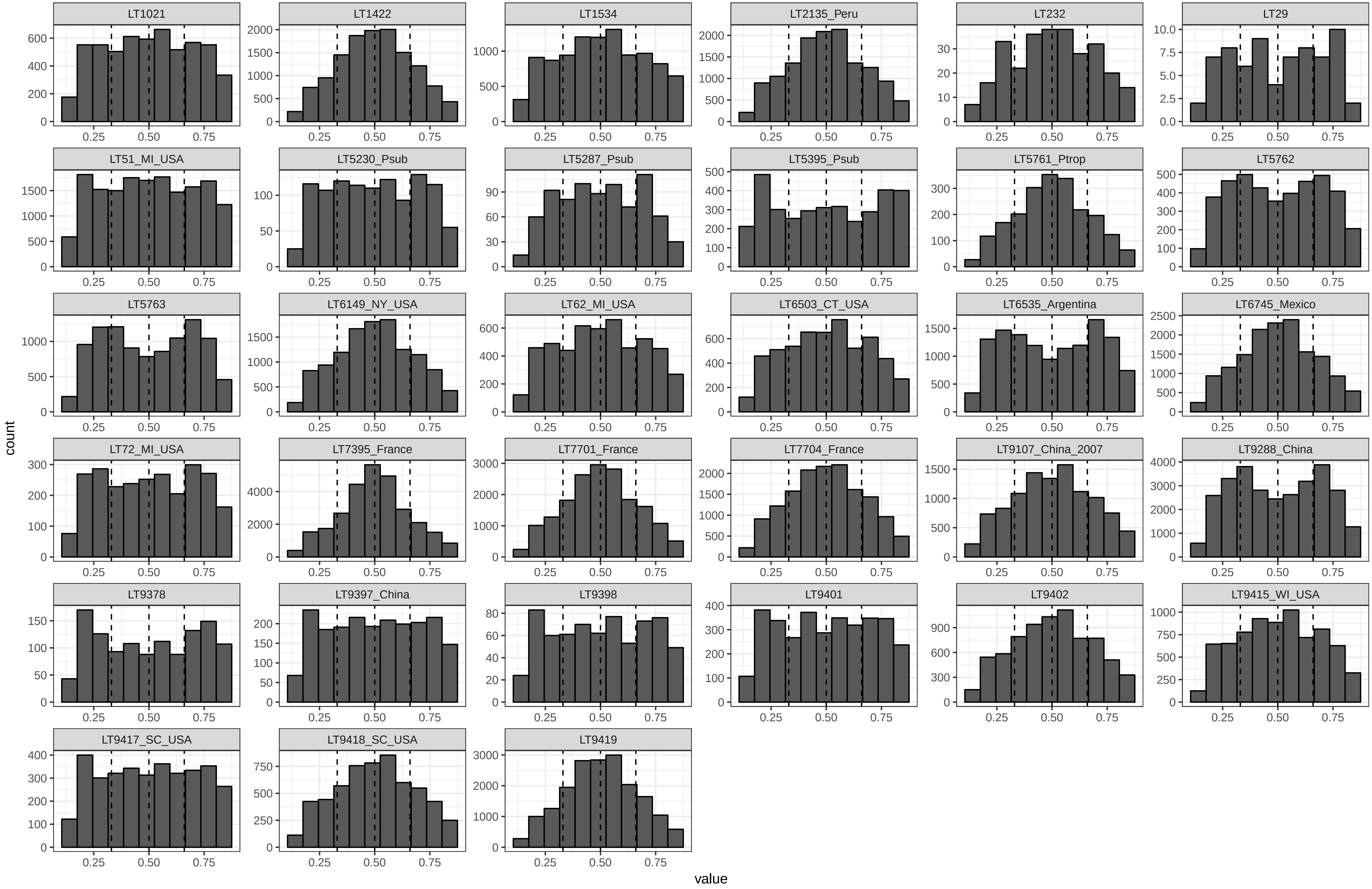

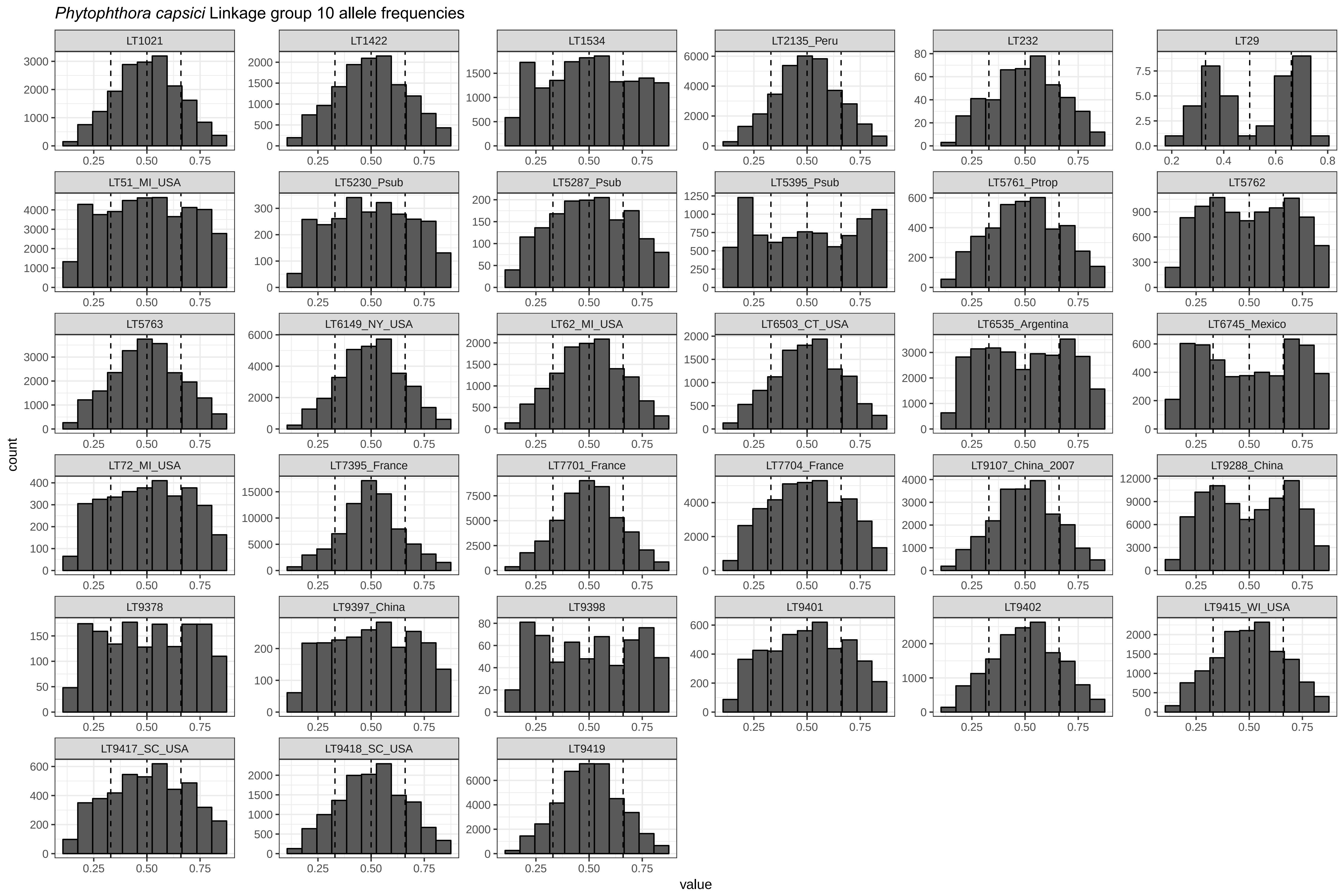
Phytophthora capsici Linkage group 10 allele frequencies
LT1021
LT1422
LT1534
LT2135_Peru
LT232
LT29
LT51_MI_USA
LT5230_Psub
LT5287_Psub
LT5395_Psub
LT5761_Ptrop
LT5762
LT5763
LT6149_NY_USA
LT62_MI_USA
LT6503_CT_USA
LT6535_Argentina
LT6745_Mexico
LT72_MI_USA
LT7395_France
LT7701_France
LT7704_France
LT9107_China_2007
LT9288_China
LT9378
LT9397_China
LT9398
LT9401
LT9402
LT9415_WI_USA
LT9417_SC_USA
LT9418_SC_USA
LT9419
count
value

Phytophthora capsici Linkage group 11 allele frequencies

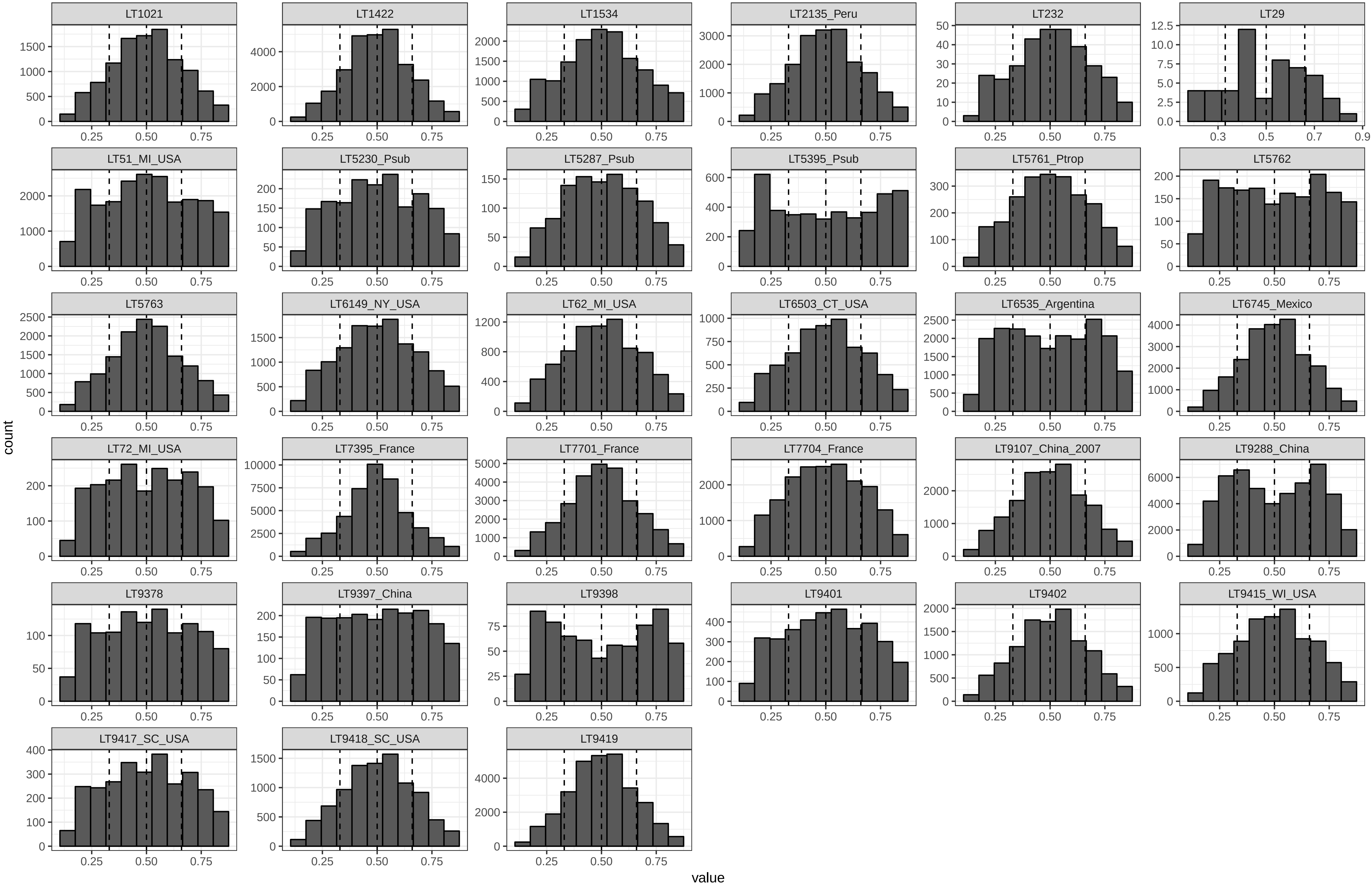

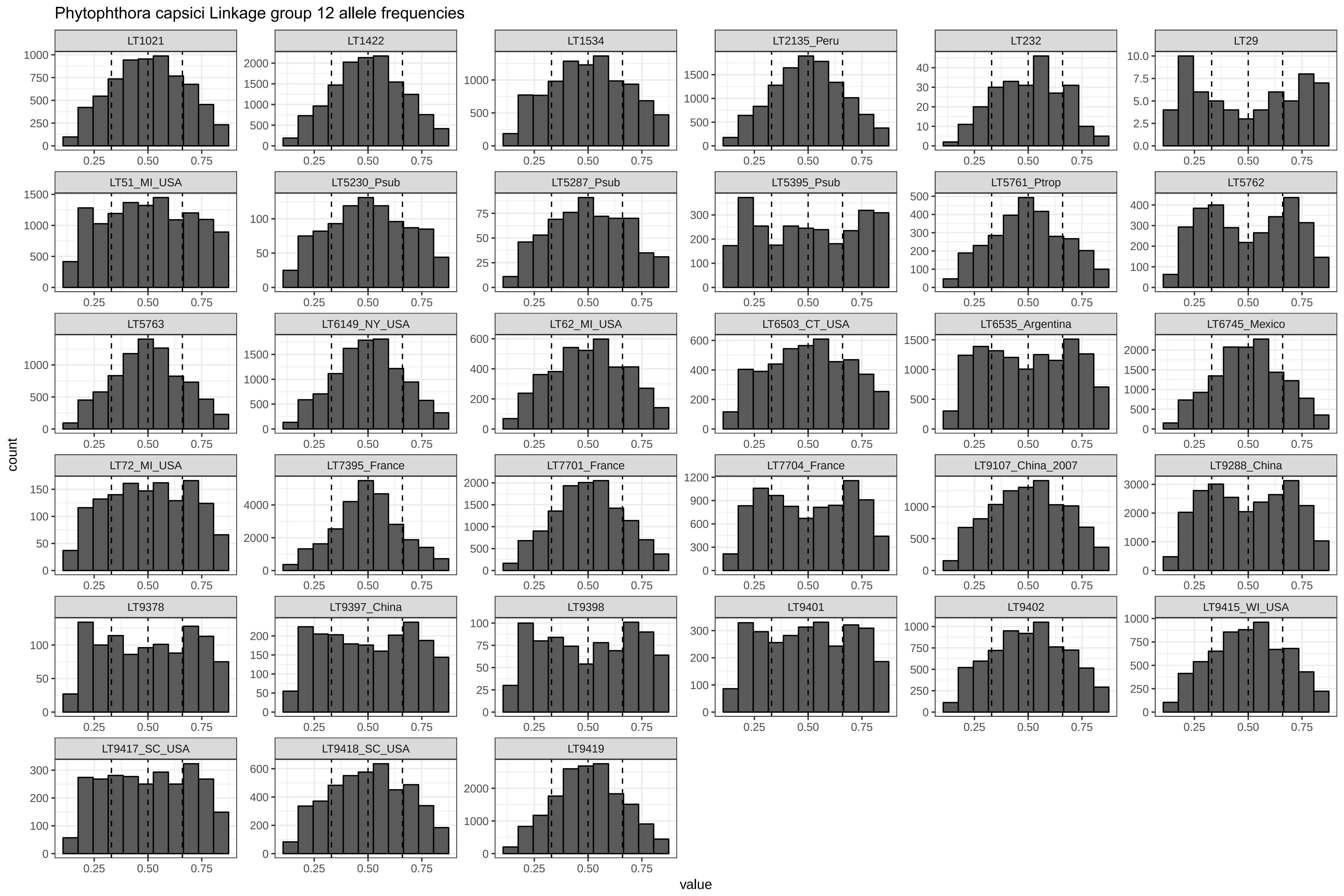

Phytophthora capsici Linkage group 12 allele frequencies
LT1021
LT1422
LT1534
LT2135_Peru
LT232
LT29
LT51_MI_USA
LT5230_Psub
LT5287_Psub
LT5395_Psub
LT5761_Ptrop
LT5762
LT5763
LT6149_NY_USA
LT62_MI_USA
LT6503_CT_USA
LT6535_Argentina
LT6745_Mexico
LT72_MI_USA
LT7395_France
LT7701_France
LT7704_France
LT9107_China_2007
LT9288_China
LT9378
LT9397_China
LT9398
LT9401
LT9402
LT9415_WI_USA
LT9417_SC_USA
LT9418_SC_USA
LT9419
count
value

Phytophthora capsici Linkage group 13 allele frequencies

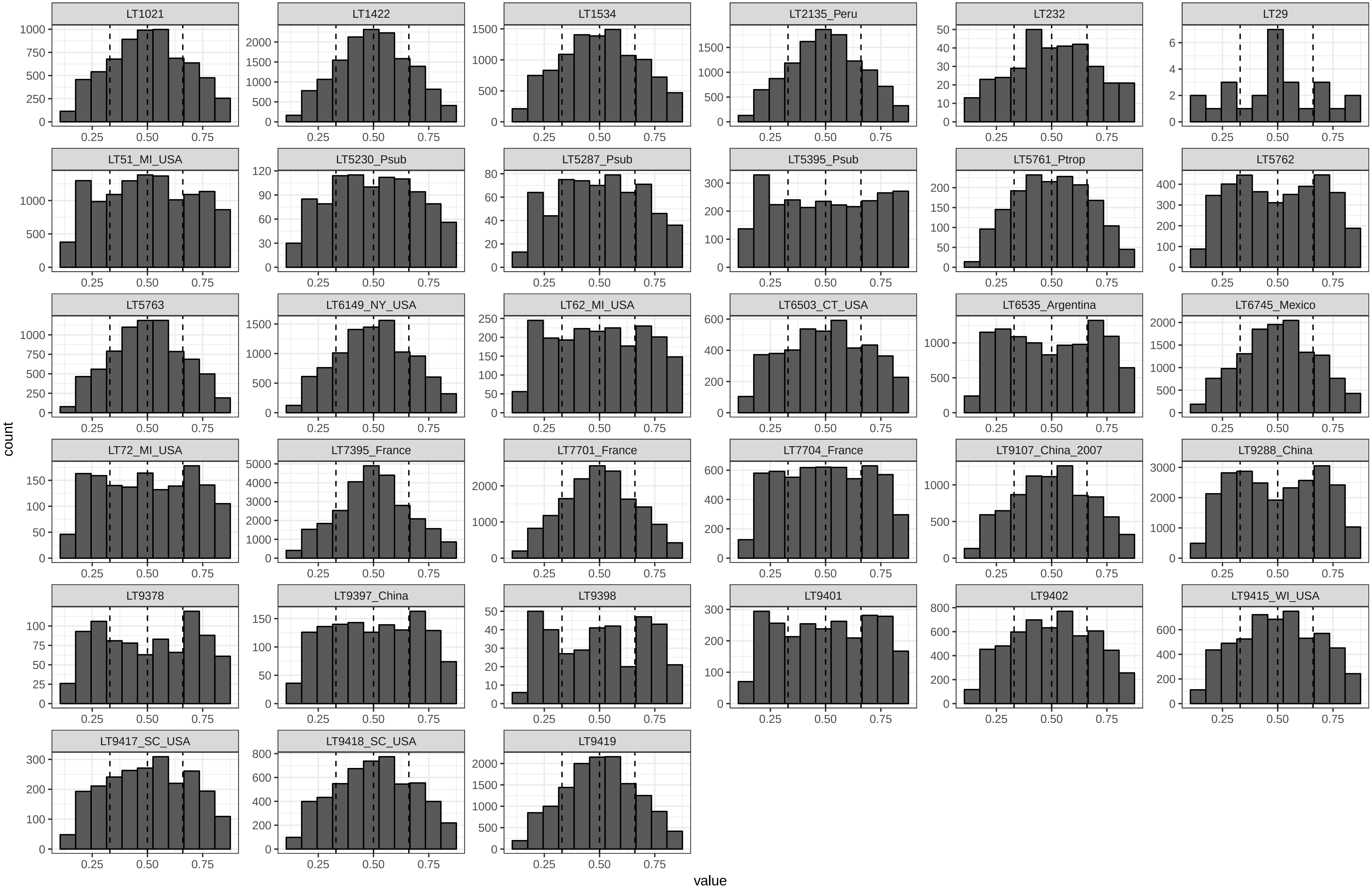

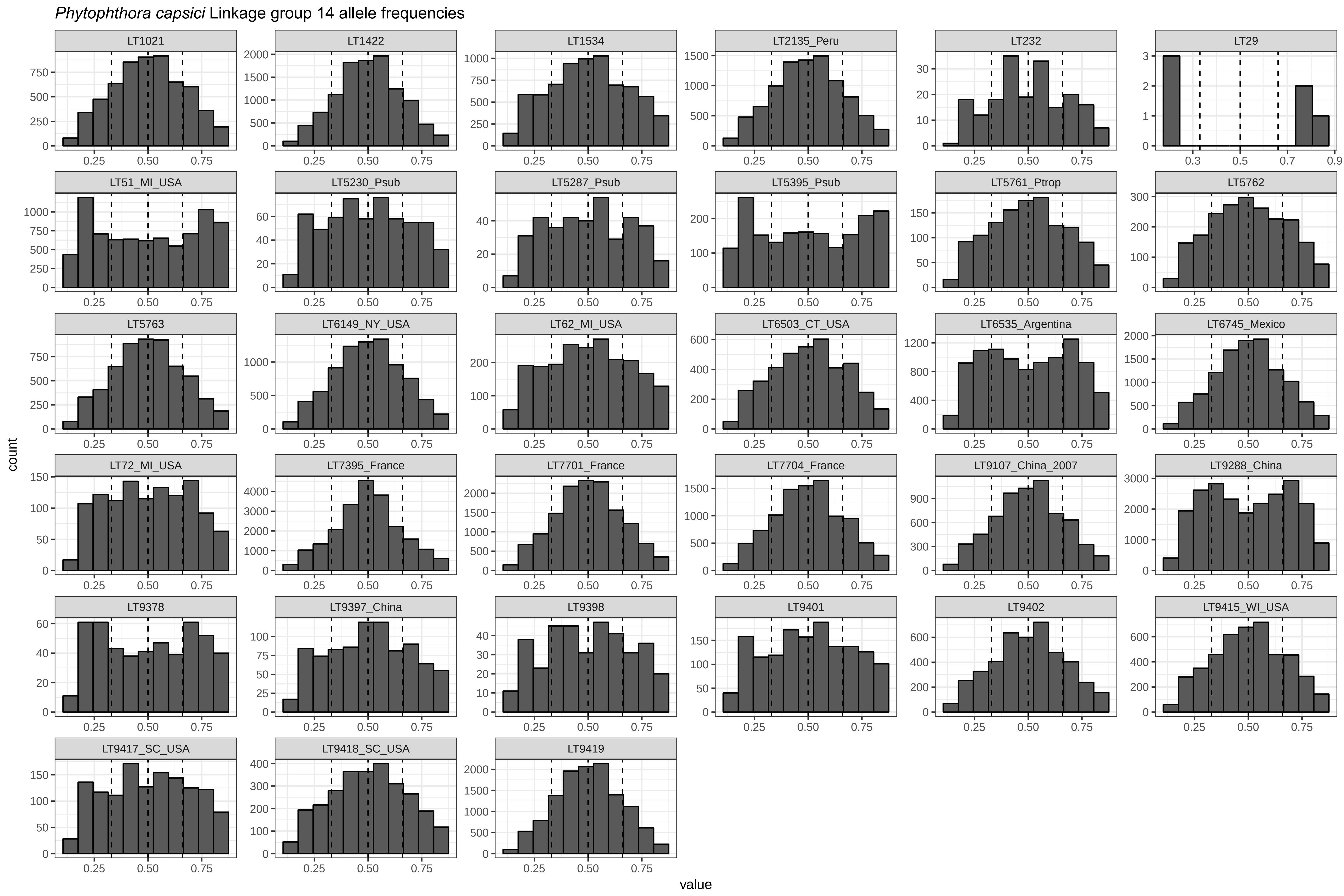
Phytophthora capsici Linkage group 14 allele frequencies
LT1021
LT1422
LT1534
LT2135_Peru
LT232
LT29
LT51_MI_USA
LT5230_Psub
LT5287_Psub
LT5395_Psub
LT5761_Ptrop
LT5762
LT5763
LT6149_NY_USA
LT62_MI_USA
LT6503_CT_USA
LT6535_Argentina
LT6745_Mexico
LT72_MI_USA
LT7395_France
LT7701_France
LT7704_France
LT9107_China_2007
LT9288_China
LT9378
LT9397_China
LT9398
LT9401
LT9402
LT9415_WI_USA
LT9417_SC_USA
LT9418_SC_USA
LT9419
count
value

Phytophthora capsici Linkage group 15 allele frequencies

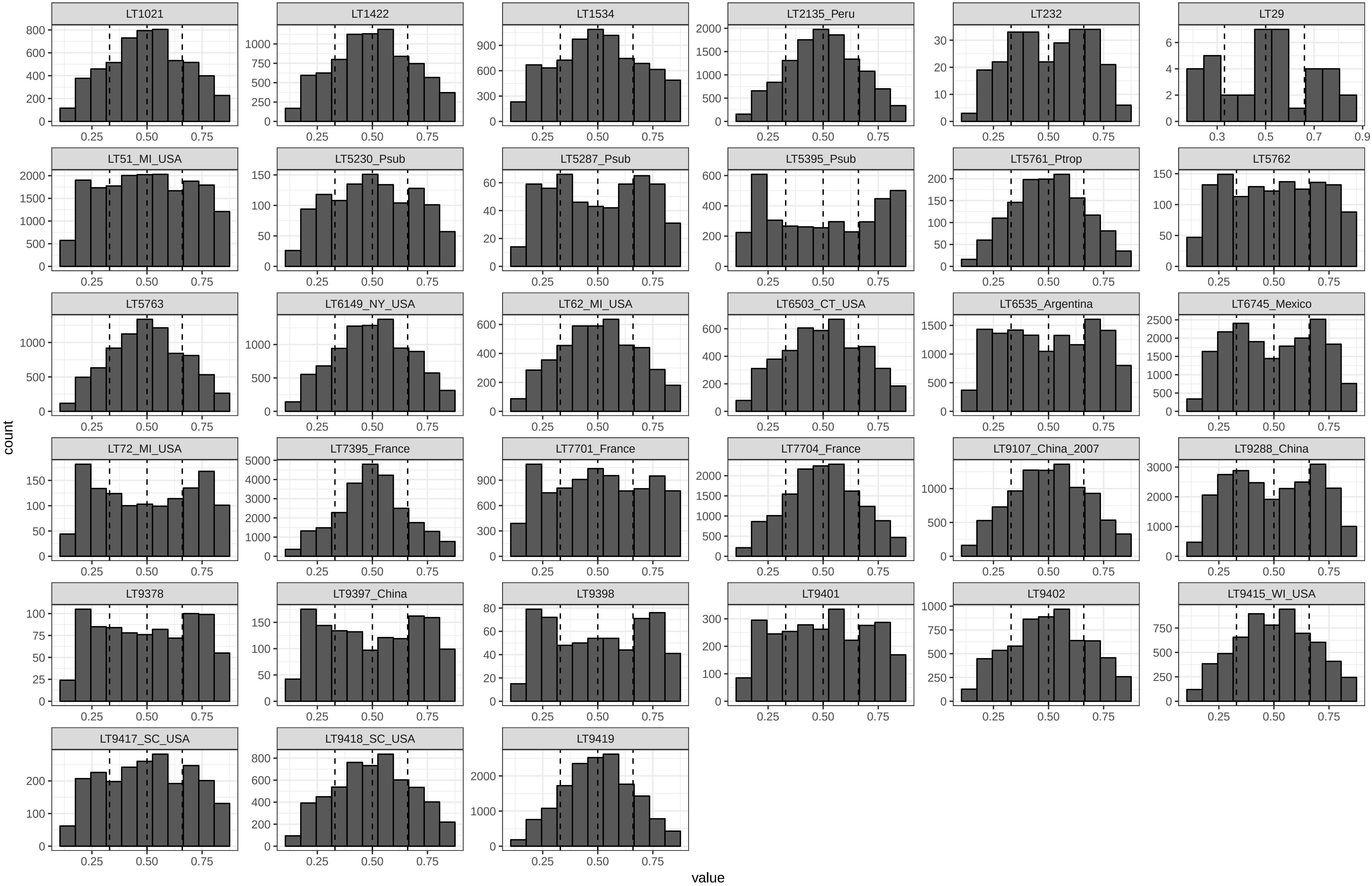

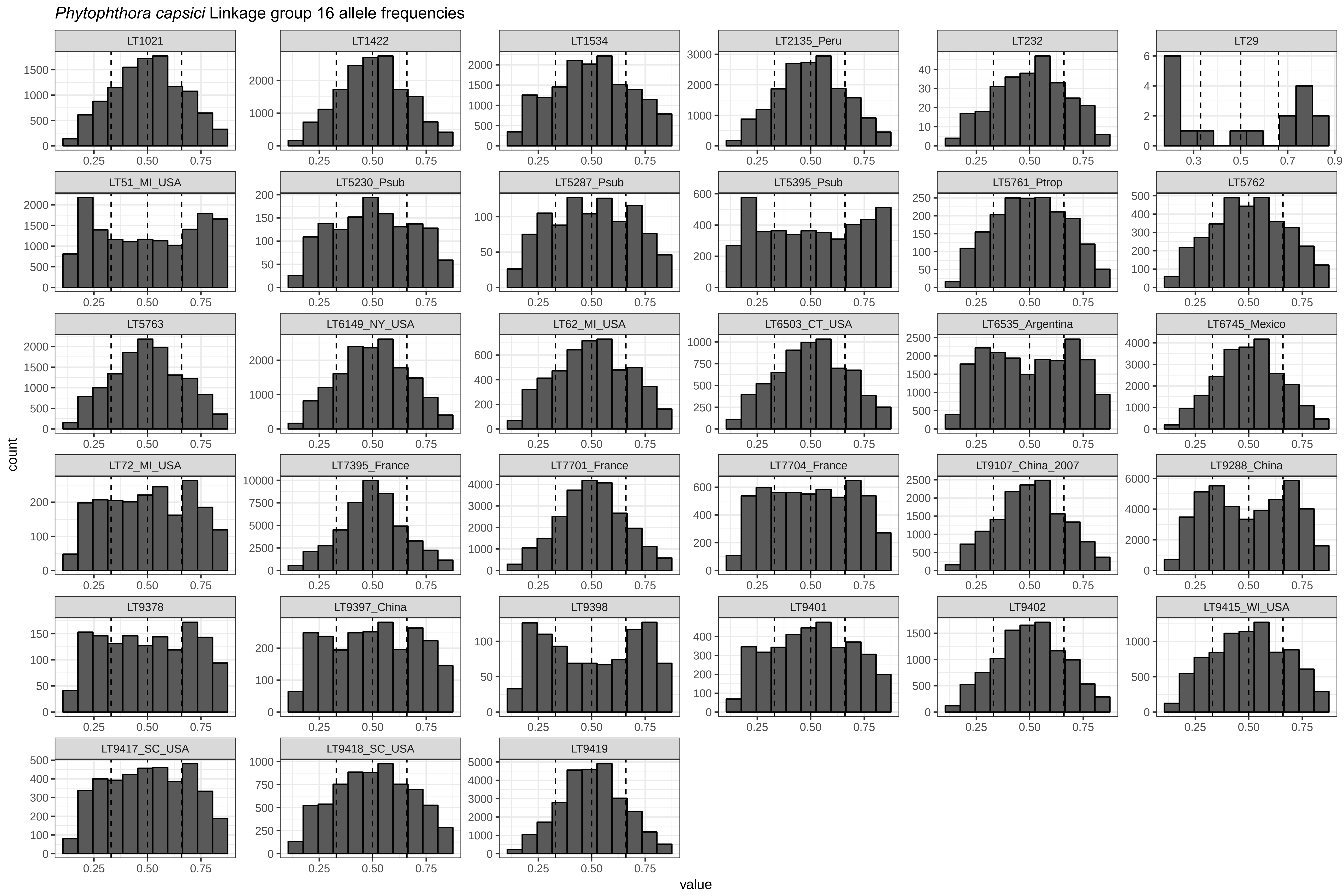

Phytophthora capsici Linkage group 16 allele frequencies
LT1021
LT1422
LT1534
LT2135_Peru
LT232
LT29
LT51_MI_USA
LT5230_Psub
LT5287_Psub
LT5395_Psub
LT5761_Ptrop
LT5762
LT5763
LT6149_NY_USA
LT62_MI_USA
LT6503_CT_USA
LT6535_Argentina
LT6745_Mexico
LT72_MI_USA
LT7395_France
LT7701_France
LT7704_France
LT9107_China_2007
LT9288_China
LT9378
LT9397_China
LT9398
LT9401
LT9402
LT9415_WI_USA
LT9417_SC_USA
LT9418_SC_USA
LT9419
count
value

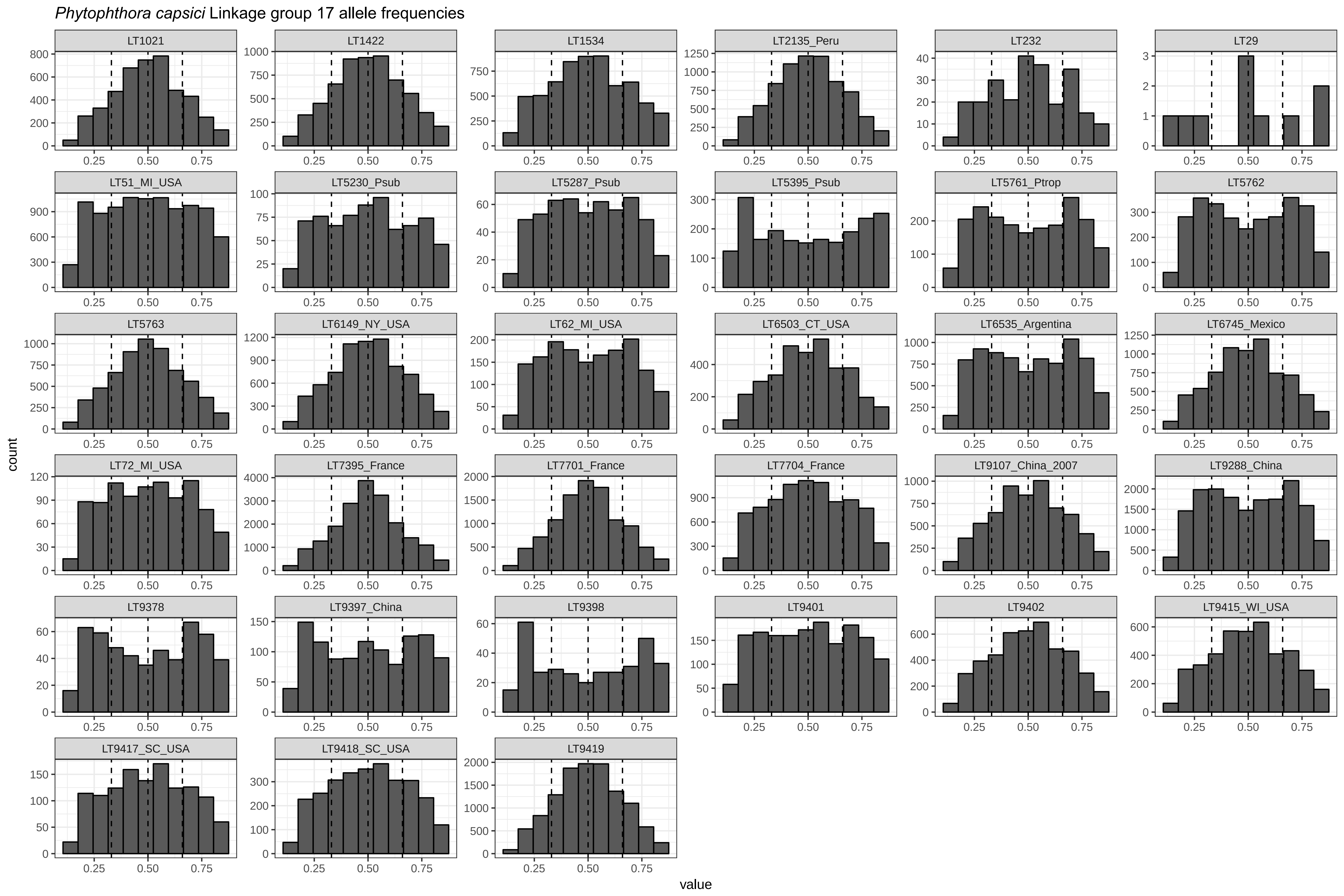

Phytophthora capsici Linkage group 17 allele frequencies
LT1021
LT1422
LT1534
LT2135_Peru
LT232
LT29
LT51_MI_USA
LT5230_Psub
LT5287_Psub
LT5395_Psub
LT5761_Ptrop
LT5762
LT5763
LT6149_NY_USA
LT62_MI_USA
LT6503_CT_USA
LT6535_Argentina
LT6745_Mexico
LT72_MI_USA
LT7395_France
LT7701_France
LT7704_France
LT9107_China_2007
LT9288_China
LT9378
LT9397_China
LT9398
LT9401
LT9402
LT9415_WI_USA
LT9417_SC_USA
LT9418_SC_USA
LT9419
count
value

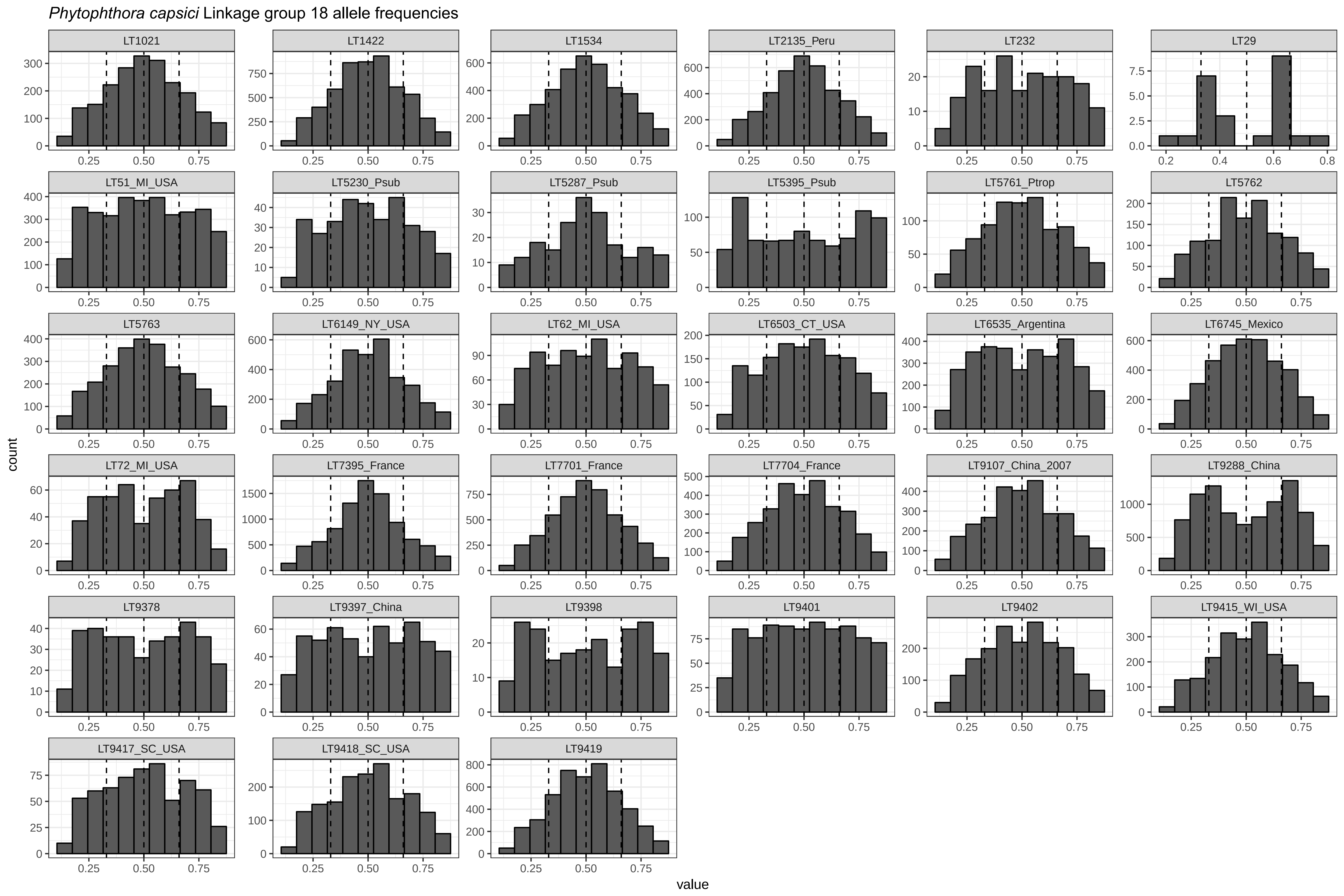
Phytophthora capsici Linkage group 18 allele frequencies
LT1021
LT1422
LT1534
LT2135_Peru
LT232
LT29
LT51_MI_USA
LT5230_Psub
LT5287_Psub
LT5395_Psub
LT5761_Ptrop
LT5762
LT5763
LT6149_NY_USA
LT62_MI_USA
LT6503_CT_USA
LT6535_Argentina
LT6745_Mexico
LT72_MI_USA
LT7395_France
LT7701_France
LT7704_France
LT9107_China_2007
LT9288_China
LT9378
LT9397_China
LT9398
LT9401
LT9402
LT9415_WI_USA
LT9417_SC_USA
LT9418_SC_USA
LT9419
count
value
