# Supplementary material for: Dynamic Extreme Aneuploidy (DEA) in the vegetable pathogen Phytophthora capsici and the potential for rapid asexual evolution
Source: PLoS One. 2020 Jan 7;15(1):e0227250. doi: 10.1371/journal.pone.0227250 (PMC6946123; doi:10.1371/journal.pone.0227250)
Supplement: S2 Fig — (PDF) [file pone.0227250.s002.pdf]

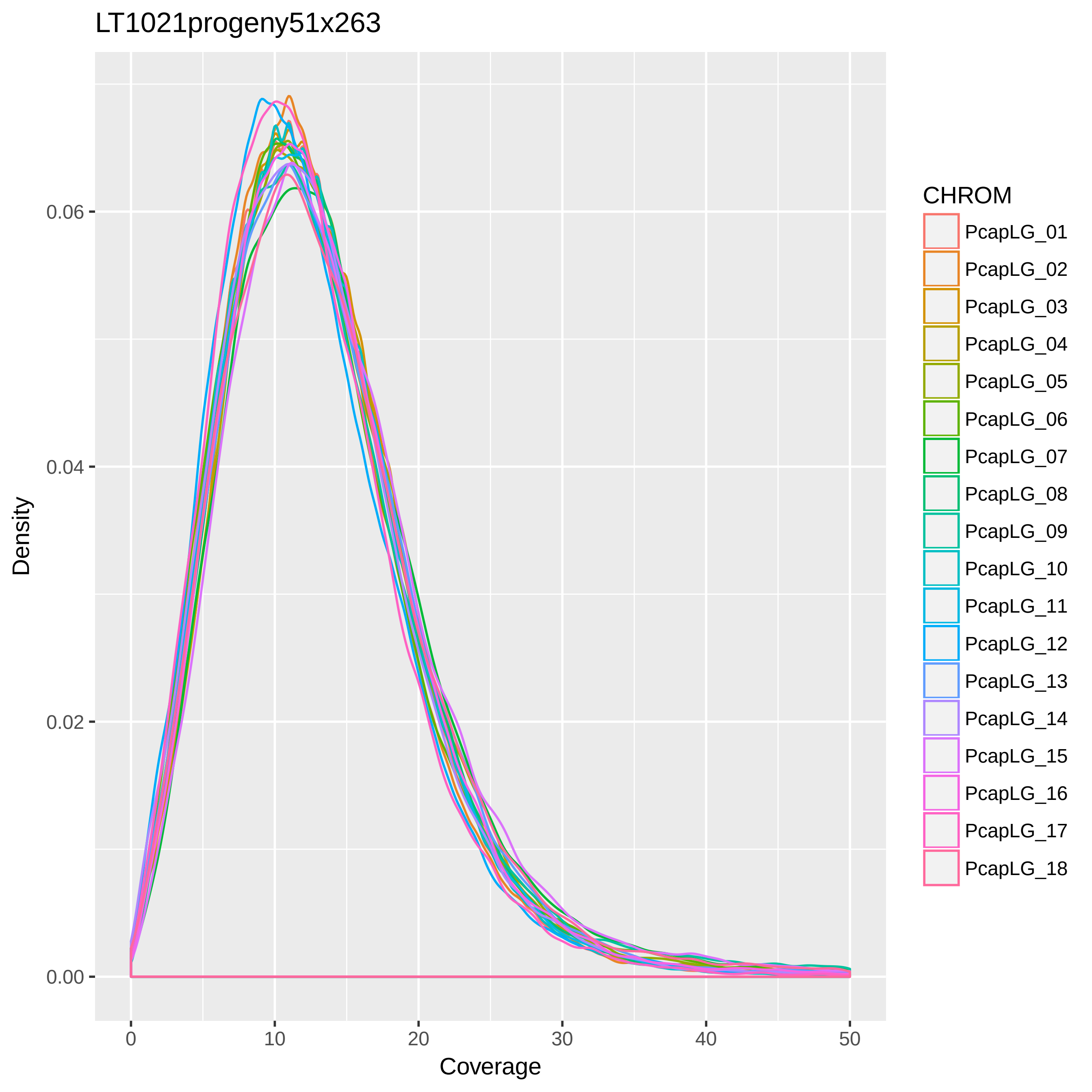
LT1021progeny51x263
Density
0.00
0.02
0.04
0.06
0
10
20
30
40
50
Coverage
CHROM
PcapLG_01
PcapLG_02
PcapLG_03
PcapLG_04
PcapLG_05
PcapLG_06
PcapLG_07
PcapLG_08
PcapLG_09
PcapLG_10
PcapLG_11
PcapLG_12
PcapLG_13
PcapLG_14
PcapLG_15
PcapLG_16
PcapLG_17
PcapLG_18

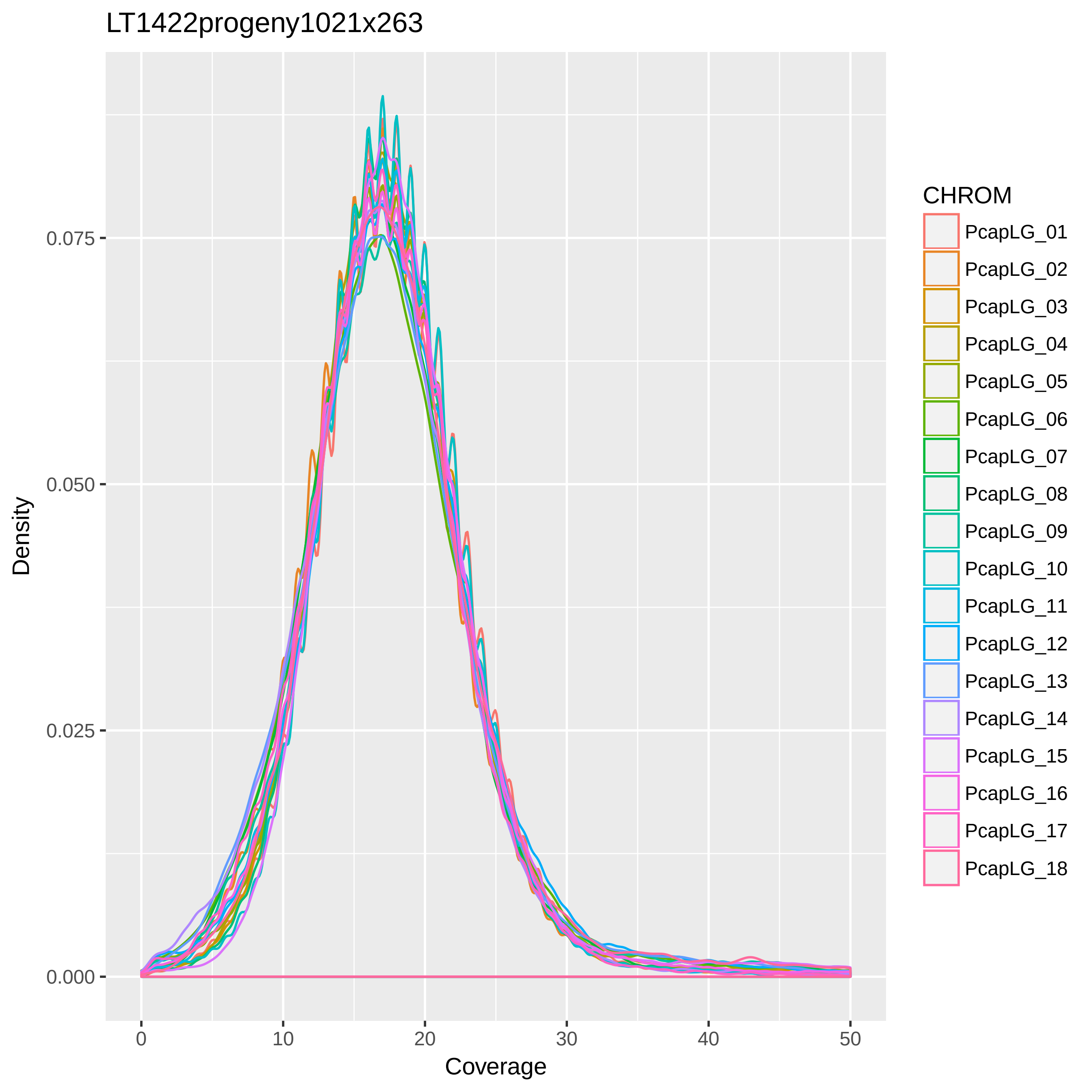

LT1422progeny1021x263
Density
Coverage
0.000
0.025
0.050
0.075
0
10
20
30
40
50
CHROM
PcapLG_01
PcapLG_02
PcapLG_03
PcapLG_04
PcapLG_05
PcapLG_06
PcapLG_07
PcapLG_08
PcapLG_09
PcapLG_10
PcapLG_11
PcapLG_12
PcapLG_13
PcapLG_14
PcapLG_15
PcapLG_16
PcapLG_17
PcapLG_18

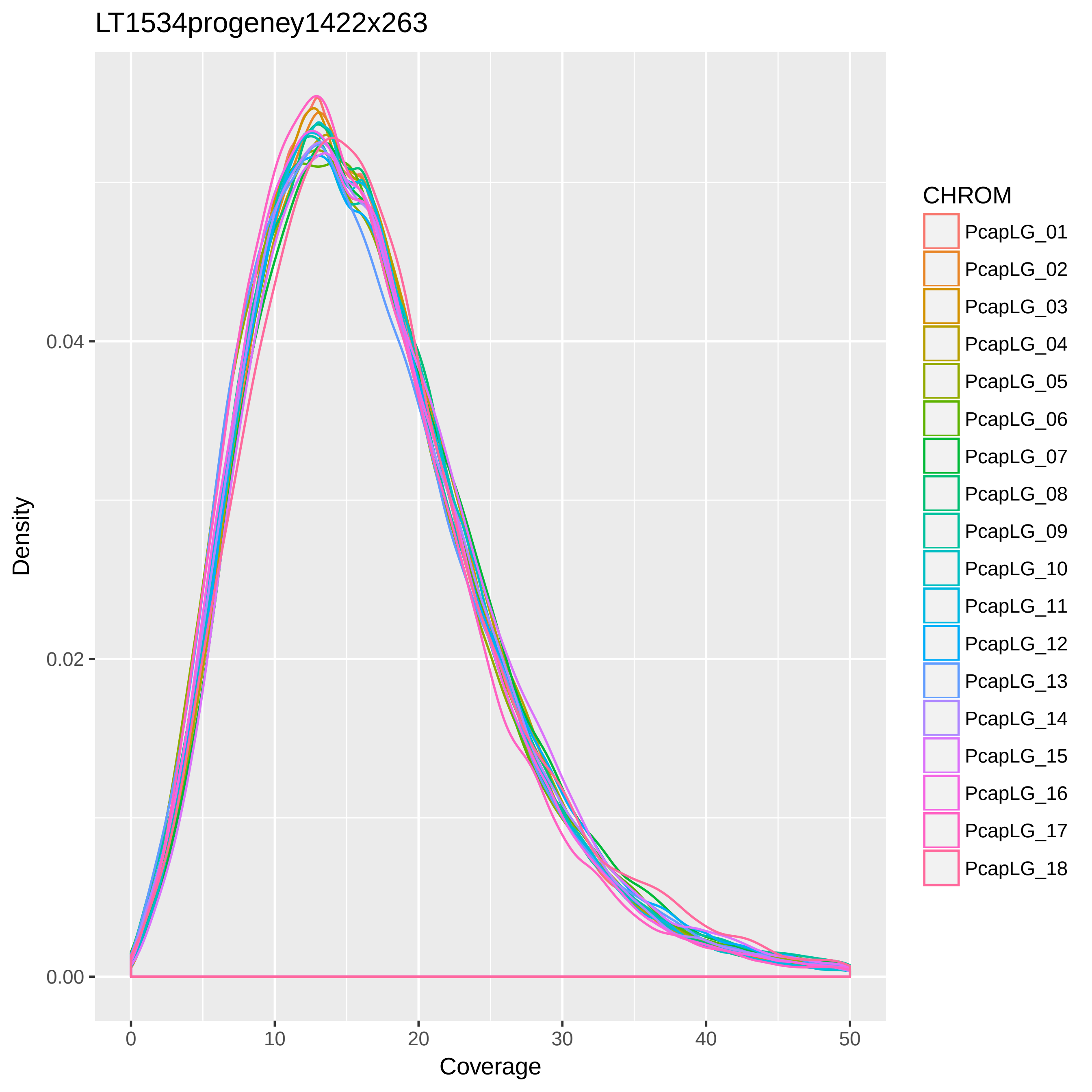
LT1534progeney1422x263
Density
0.04
0.02
0.00
0
10
20
30
40
50
Coverage
CHROM
PcapLG_01
PcapLG_02
PcapLG_03
PcapLG_04
PcapLG_05
PcapLG_06
PcapLG_07
PcapLG_08
PcapLG_09
PcapLG_10
PcapLG_11
PcapLG_12
PcapLG_13
PcapLG_14
PcapLG_15
PcapLG_16
PcapLG_17
PcapLG_18

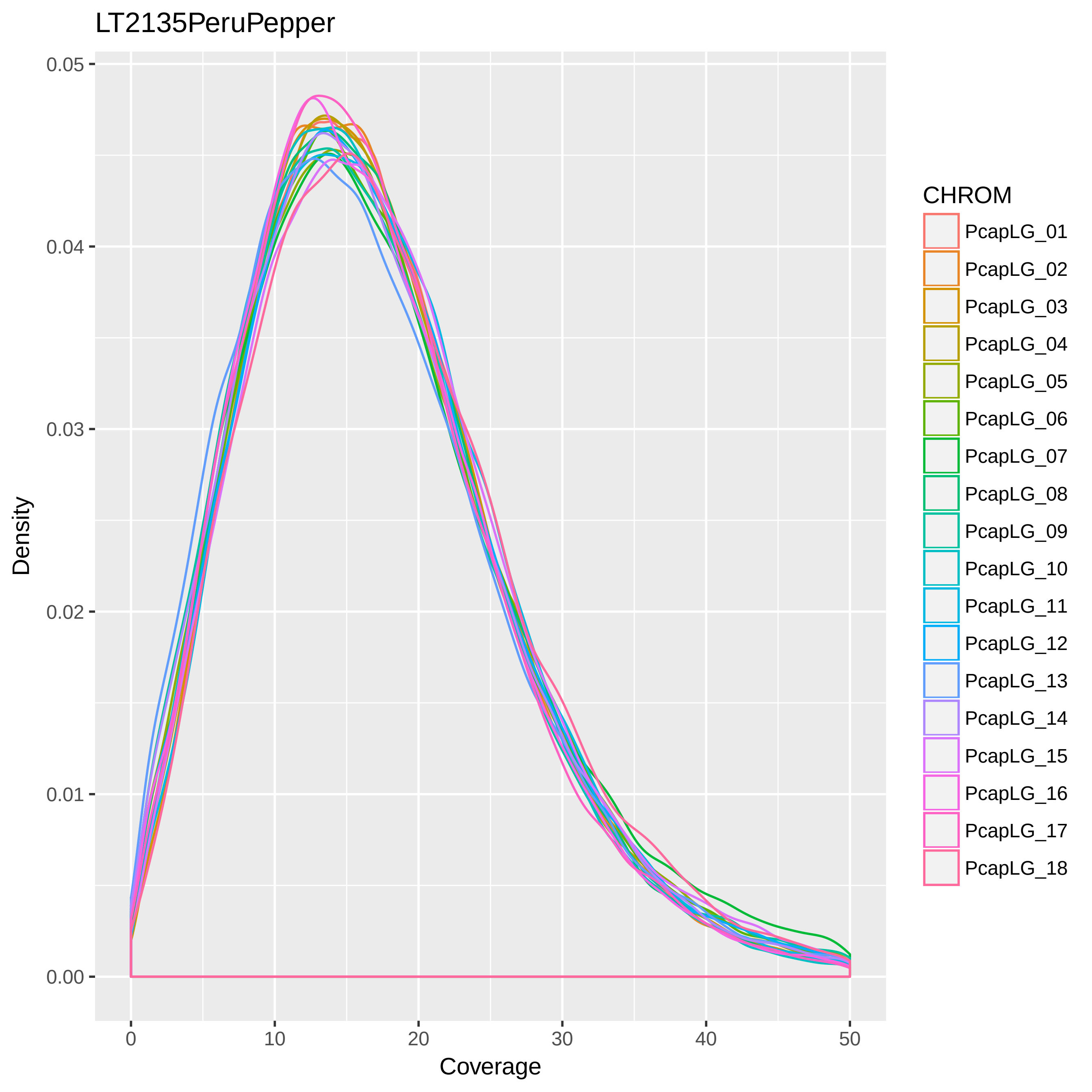
LT2135PeruPepper
Density
Coverage
0.05
0.04
0.03
0.02
0.01
0.00
0
10
20
30
40
50
CHROM
PcapLG_01
PcapLG_02
PcapLG_03
PcapLG_04
PcapLG_05
PcapLG_06
PcapLG_07
PcapLG_08
PcapLG_09
PcapLG_10
PcapLG_11
PcapLG_12
PcapLG_13
PcapLG_14
PcapLG_15
PcapLG_16
PcapLG_17
PcapLG_18

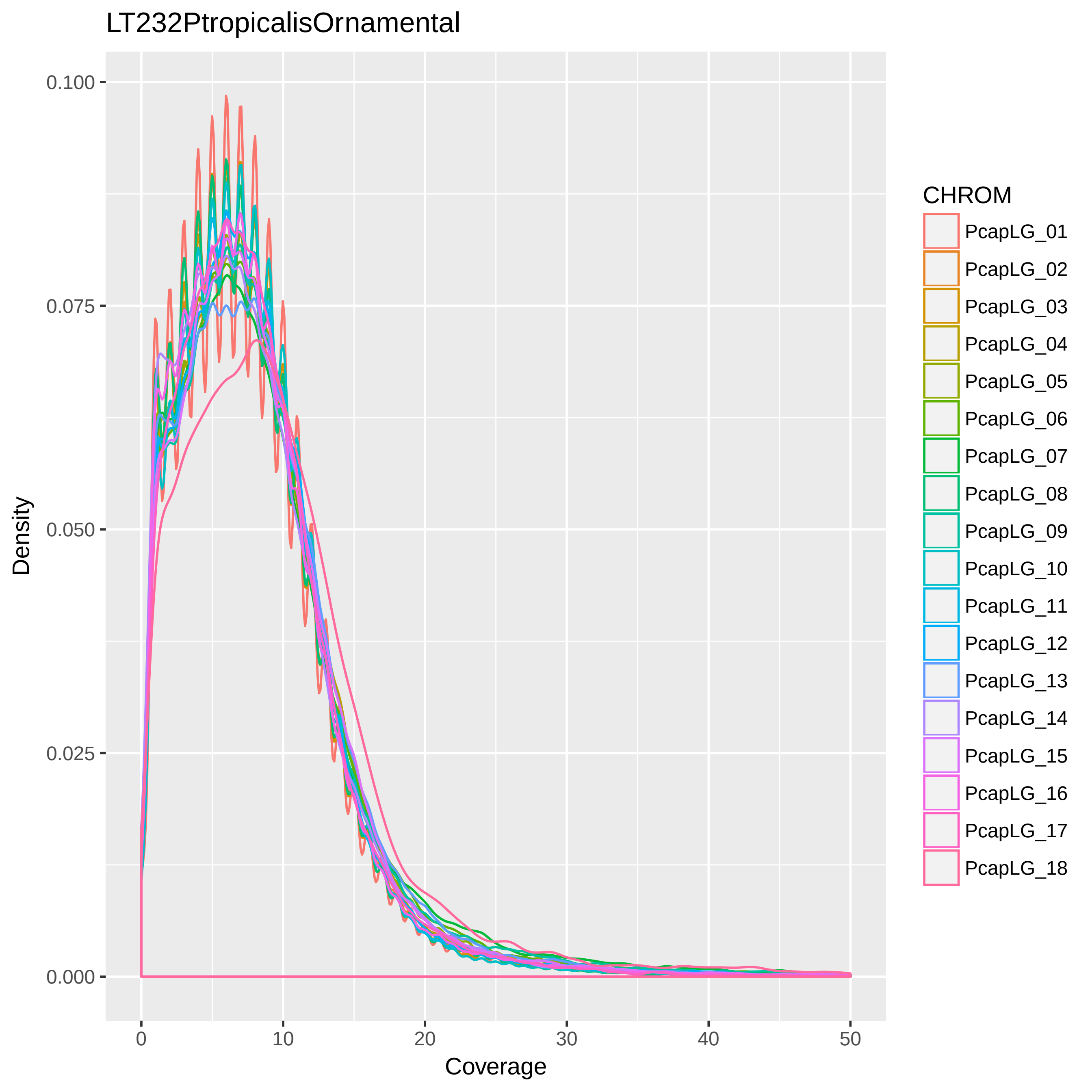

LT232PtropicalisOrnamental
0.100
0.075
0.050
0.025
0.000
Density
0
10
20
30
40
50
Coverage
CHROM
PcapLG_01
PcapLG_02
PcapLG_03
PcapLG_04
PcapLG_05
PcapLG_06
PcapLG_07
PcapLG_08
PcapLG_09
PcapLG_10
PcapLG_11
PcapLG_12
PcapLG_13
PcapLG_14
PcapLG_15
PcapLG_16
PcapLG_17
PcapLG_18

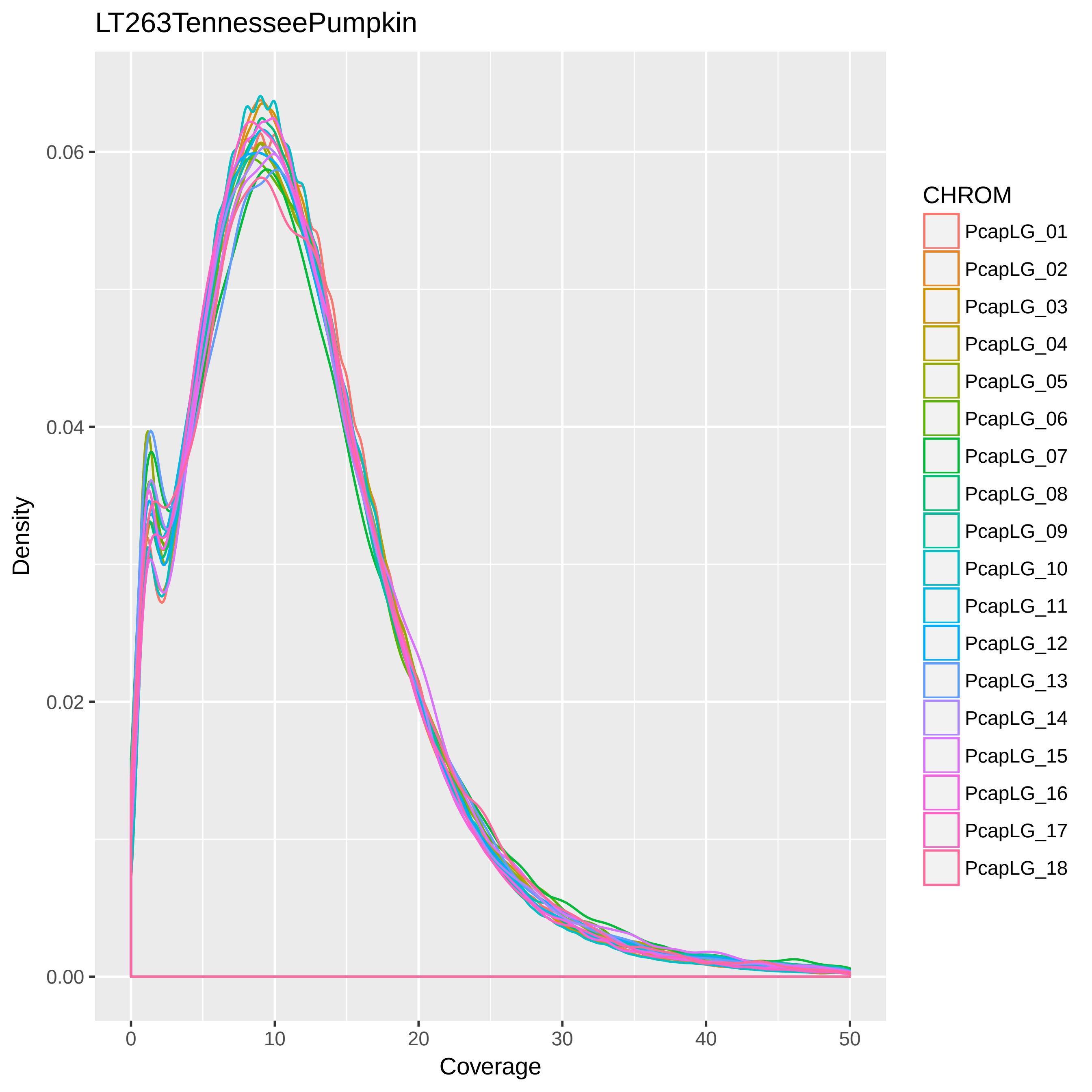
LT263TennesseePumpkin
Density
0.00
0.02
0.04
0.06
Coverage
0
10
20
30
40
50
CHROM
PcapLG_01
PcapLG_02
PcapLG_03
PcapLG_04
PcapLG_05
PcapLG_06
PcapLG_07
PcapLG_08
PcapLG_09
PcapLG_10
PcapLG_11
PcapLG_12
PcapLG_13
PcapLG_14
PcapLG_15
PcapLG_16
PcapLG_17
PcapLG_18

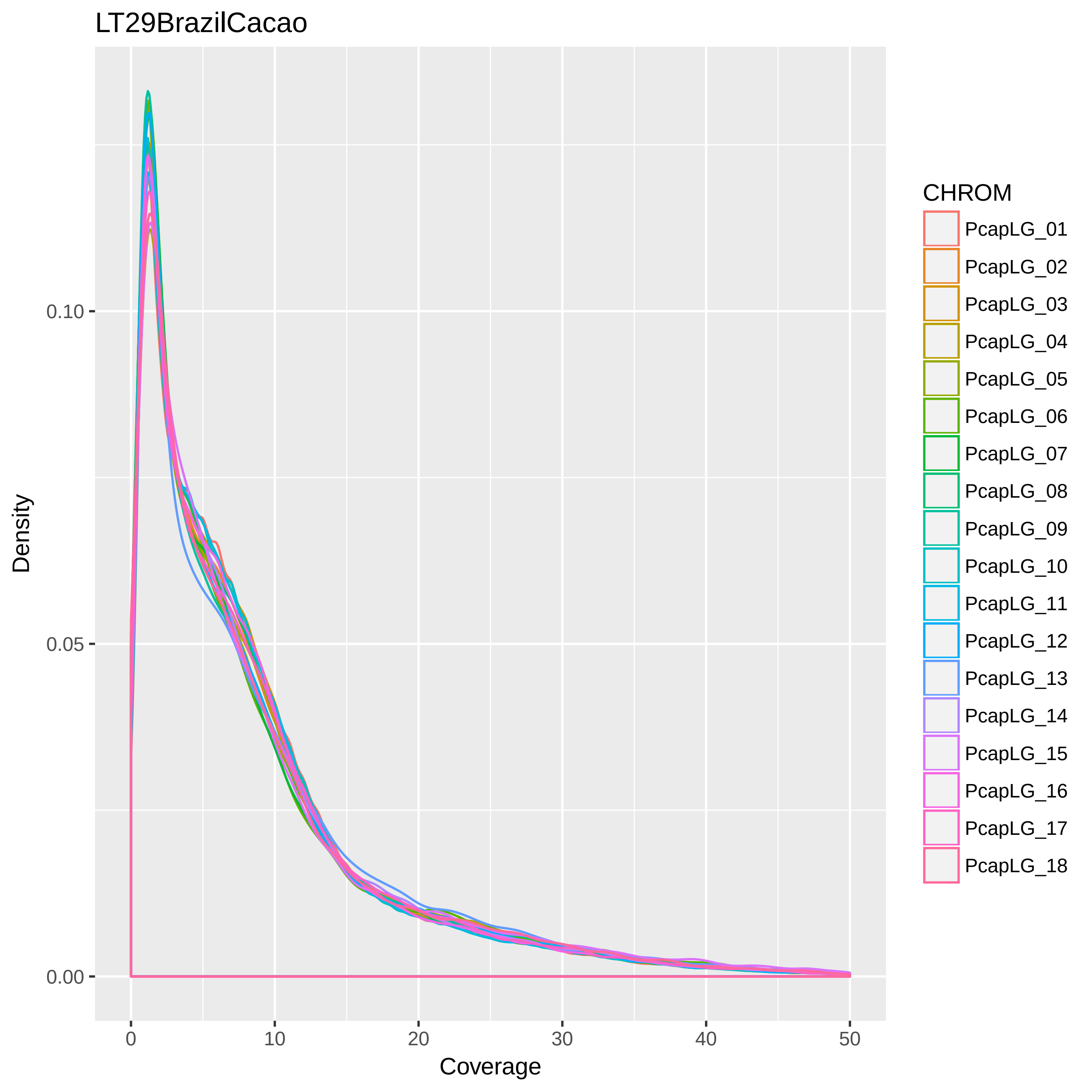
LT29BrazilCacao
Density
0.10
0.05
0.00
0
10
20
30
40
50
Coverage
CHROM
PcapLG_01
PcapLG_02
PcapLG_03
PcapLG_04
PcapLG_05
PcapLG_06
PcapLG_07
PcapLG_08
PcapLG_09
PcapLG_10
PcapLG_11
PcapLG_12
PcapLG_13
PcapLG_14
PcapLG_15
PcapLG_16
PcapLG_17
PcapLG_18

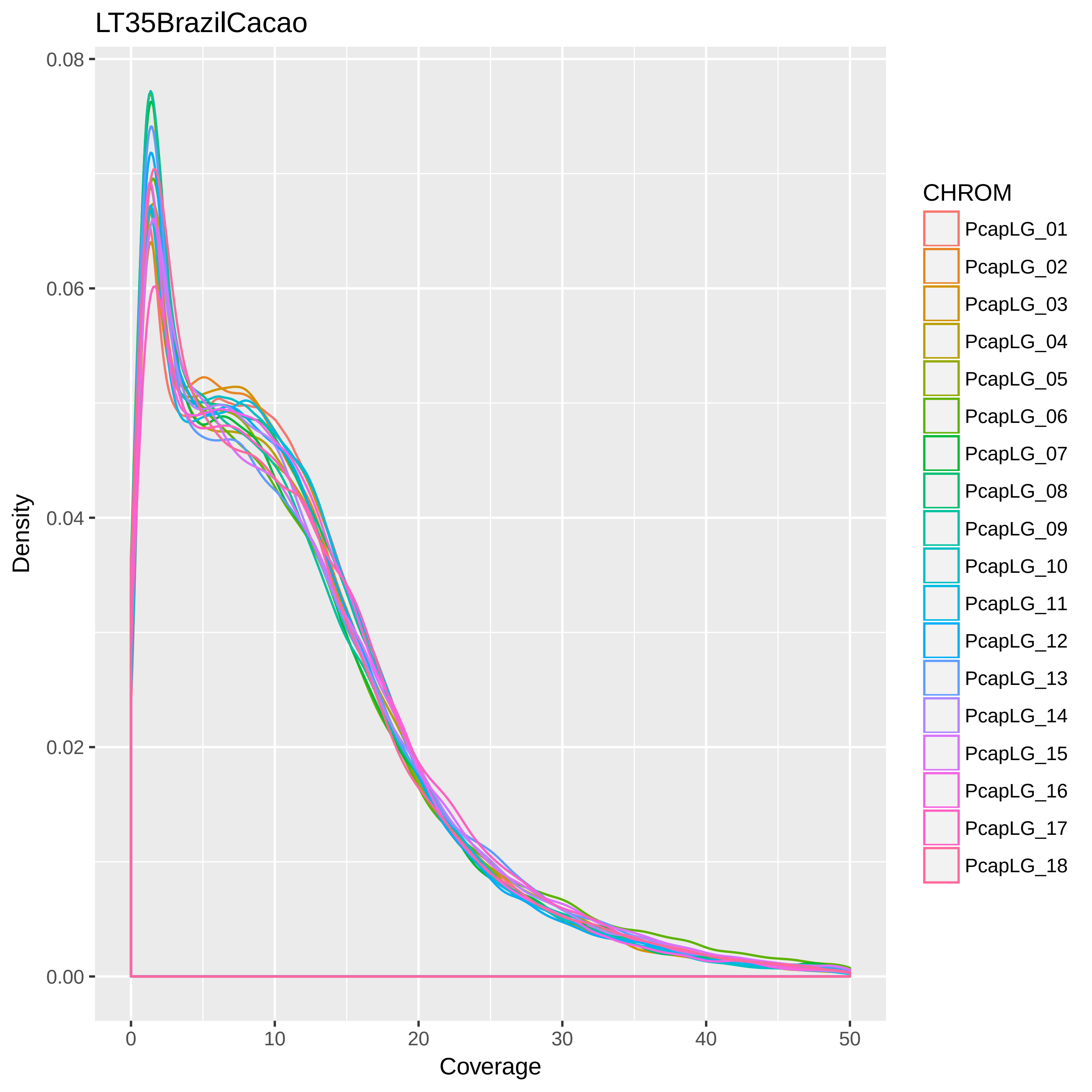
LT35BrazilCacao
0.08
0.06
0.04
0.02
0.00
Density
0
10
20
30
40
50
Coverage
CHROM
PcapLG_01
PcapLG_02
PcapLG_03
PcapLG_04
PcapLG_05
PcapLG_06
PcapLG_07
PcapLG_08
PcapLG_09
PcapLG_10
PcapLG_11
PcapLG_12
PcapLG_13
PcapLG_14
PcapLG_15
PcapLG_16
PcapLG_17
PcapLG_18

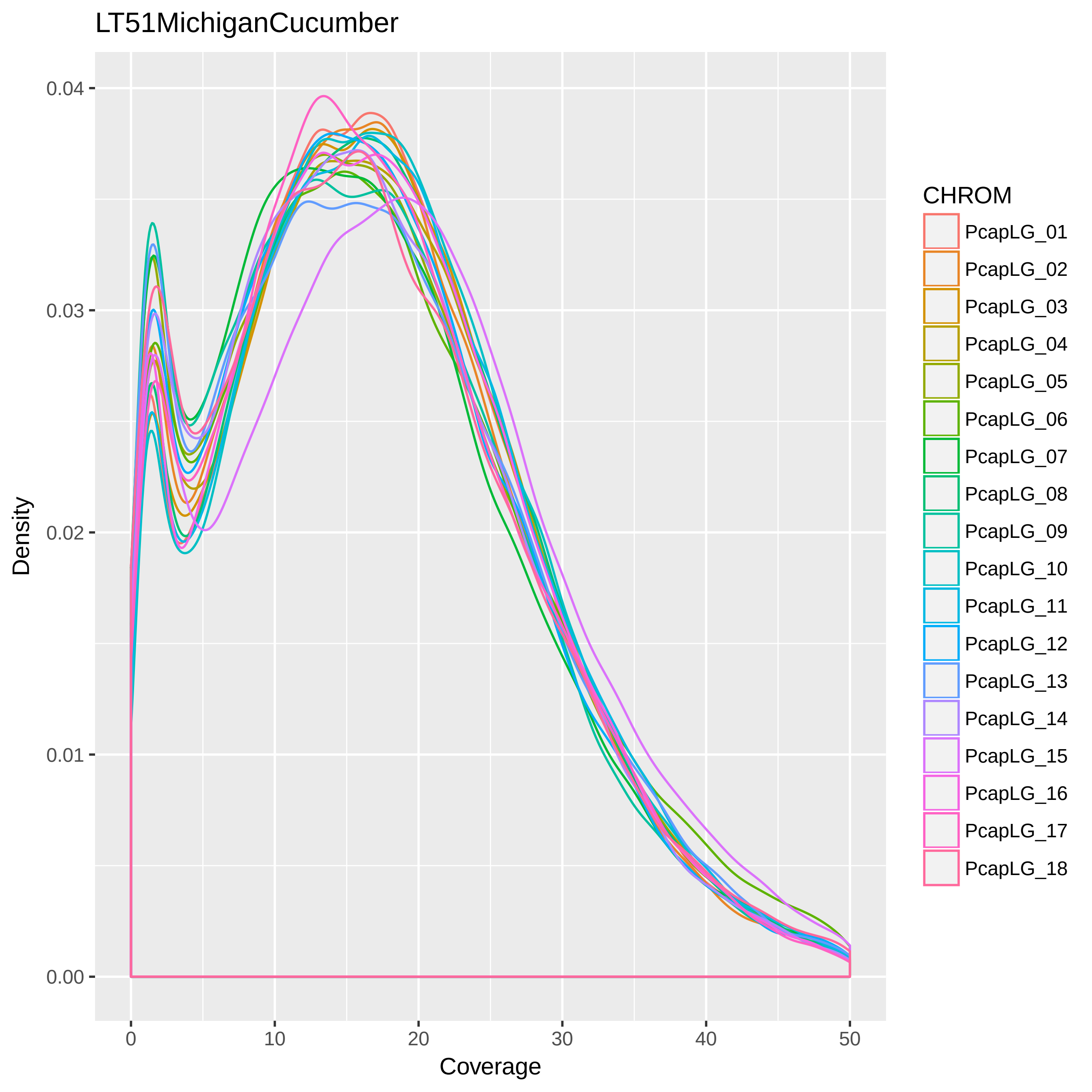

LT51MichiganCucumber
0.04
0.03
0.02
0.01
0.00
Density
0
10
20
30
40
50
Coverage
CHROM
PcapLG_01
PcapLG_02
PcapLG_03
PcapLG_04
PcapLG_05
PcapLG_06
PcapLG_07
PcapLG_08
PcapLG_09
PcapLG_10
PcapLG_11
PcapLG_12
PcapLG_13
PcapLG_14
PcapLG_15
PcapLG_16
PcapLG_17
PcapLG_18

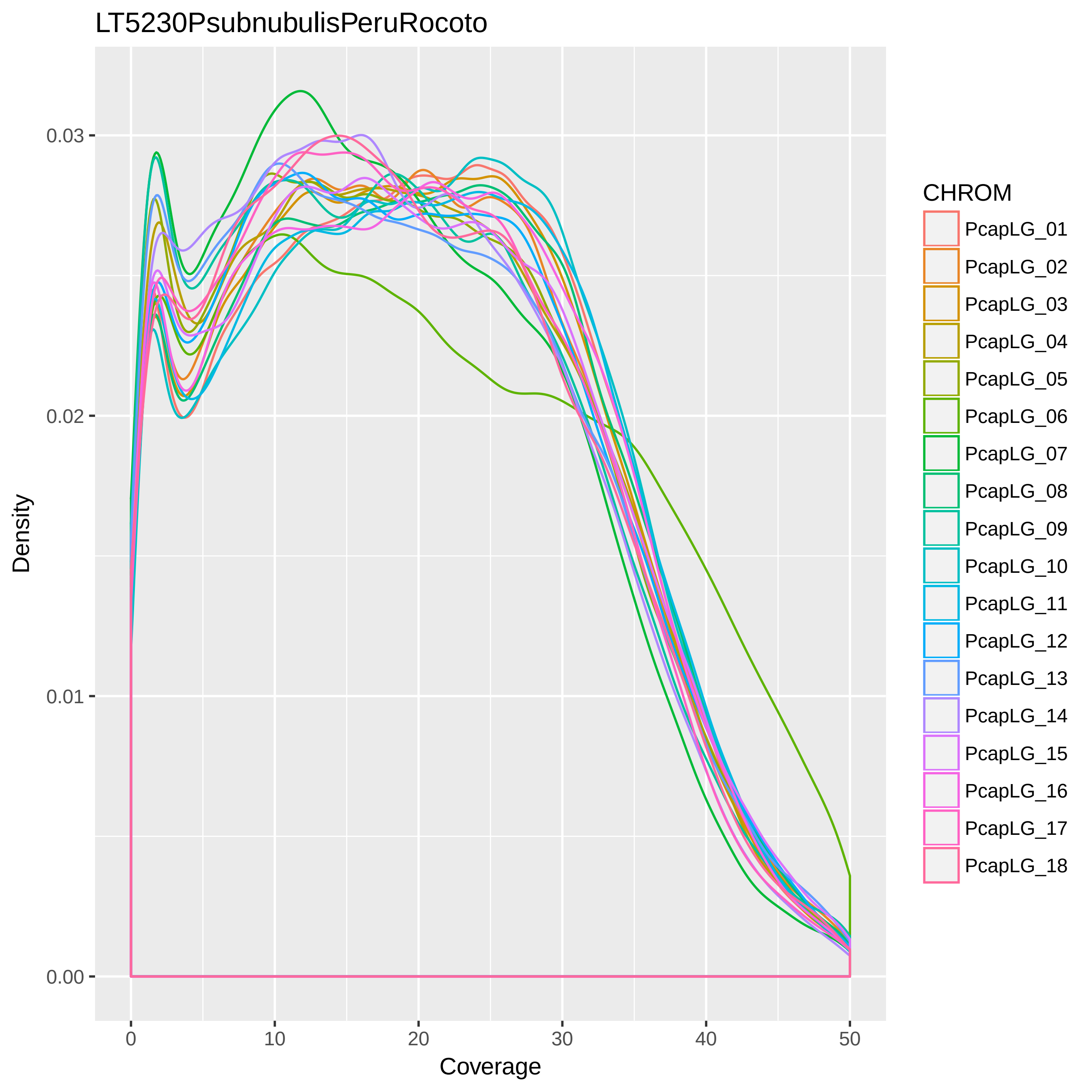

LT5230PsubnubulisPeruRocoto
Density
Coverage
0.00
0.01
0.02
0.03
0
10
20
30
40
50
CHROM
PcapLG_01
PcapLG_02
PcapLG_03
PcapLG_04
PcapLG_05
PcapLG_06
PcapLG_07
PcapLG_08
PcapLG_09
PcapLG_10
PcapLG_11
PcapLG_12
PcapLG_13
PcapLG_14
PcapLG_15
PcapLG_16
PcapLG_17
PcapLG_18

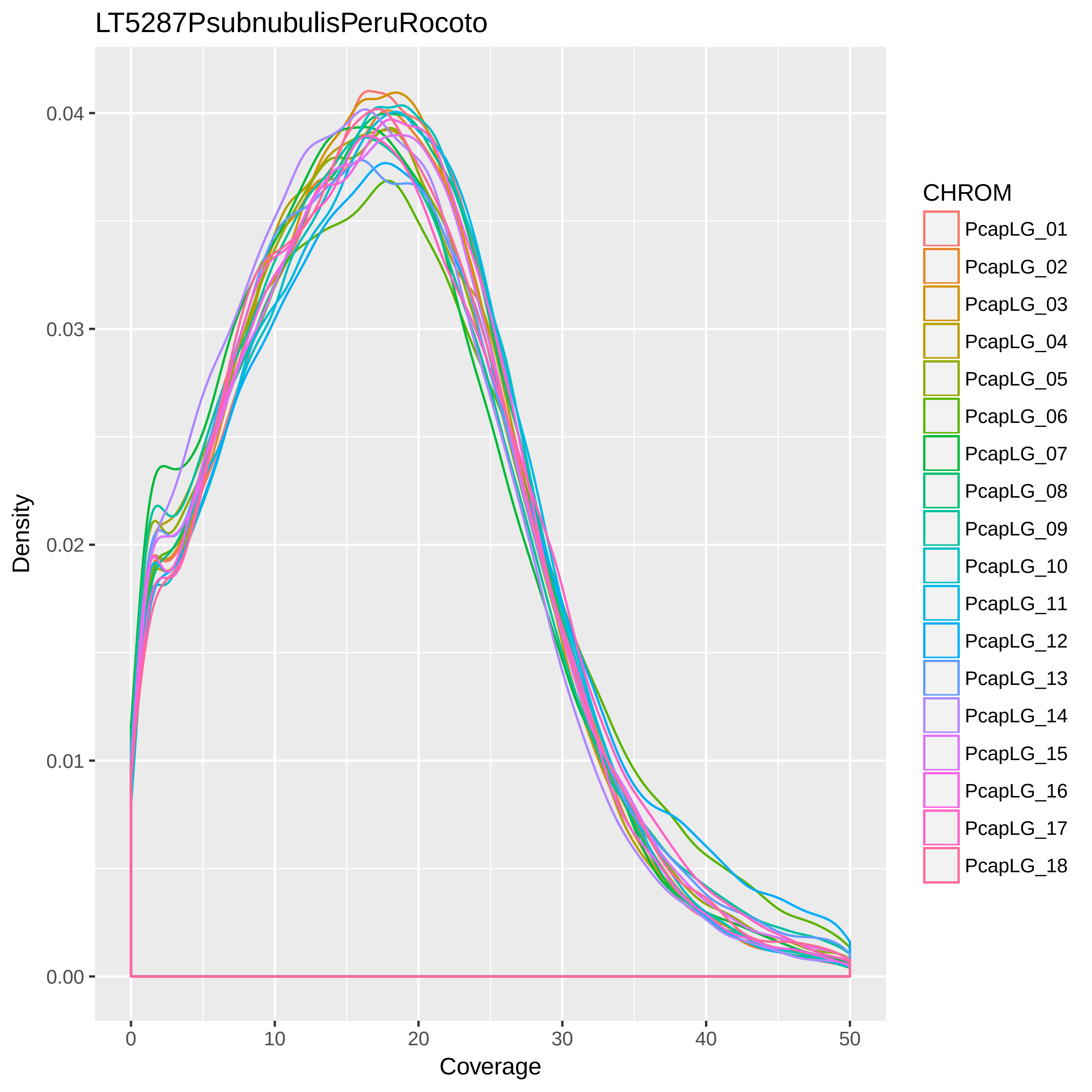

LT5287PsubnubulisPeruRocoto
Density
Coverage
0.00
0.01
0.02
0.03
0.04
0
10
20
30
40
50
CHROM
PcapLG_01
PcapLG_02
PcapLG_03
PcapLG_04
PcapLG_05
PcapLG_06
PcapLG_07
PcapLG_08
PcapLG_09
PcapLG_10
PcapLG_11
PcapLG_12
PcapLG_13
PcapLG_14
PcapLG_15
PcapLG_16
PcapLG_17
PcapLG_18

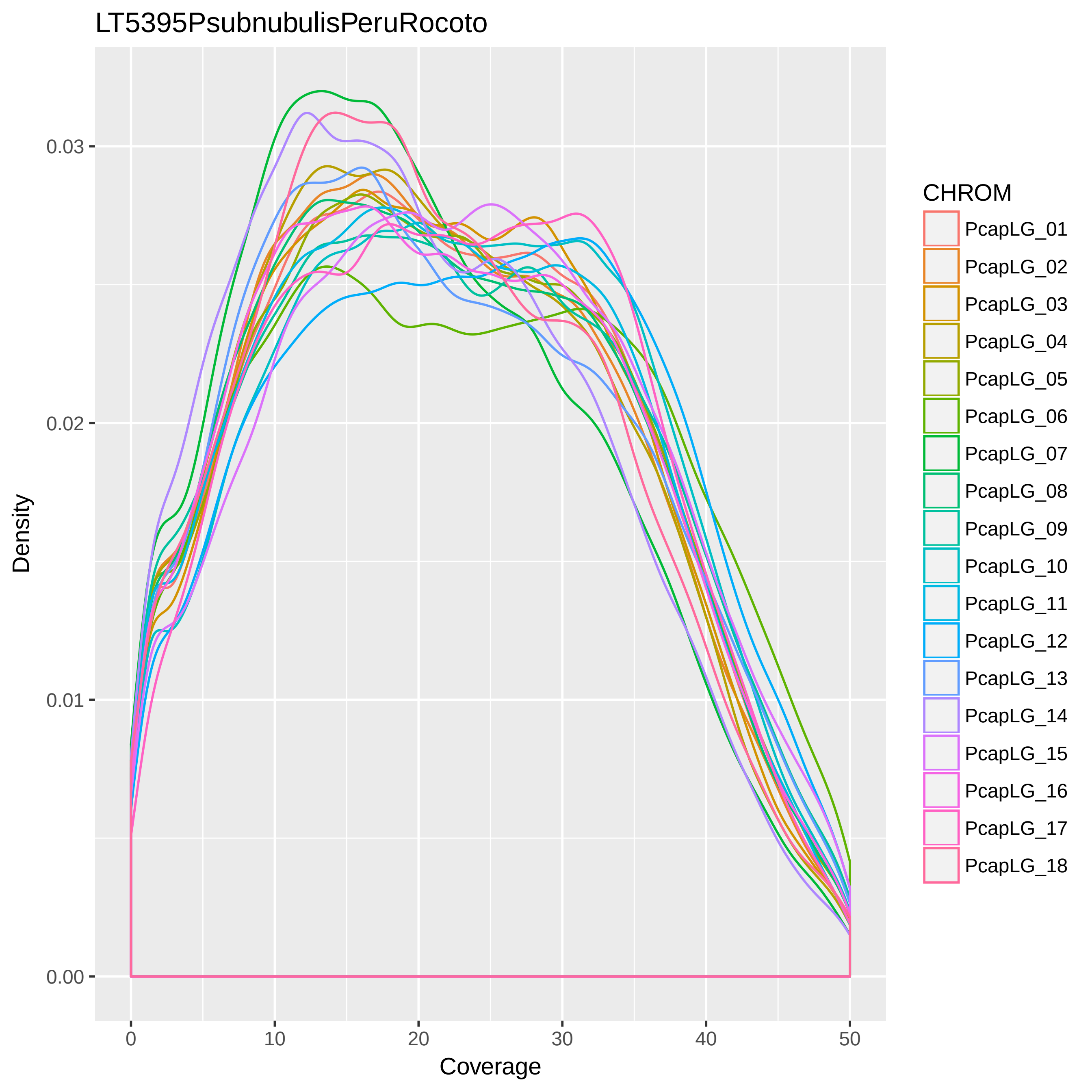
LT5395PsubnubulisPeruRocoto
Density
Coverage
0.00
0.01
0.02
0.03
0
10
20
30
40
50
CHROM
PcapLG_01
PcapLG_02
PcapLG_03
PcapLG_04
PcapLG_05
PcapLG_06
PcapLG_07
PcapLG_08
PcapLG_09
PcapLG_10
PcapLG_11
PcapLG_12
PcapLG_13
PcapLG_14
PcapLG_15
PcapLG_16
PcapLG_17
PcapLG_18

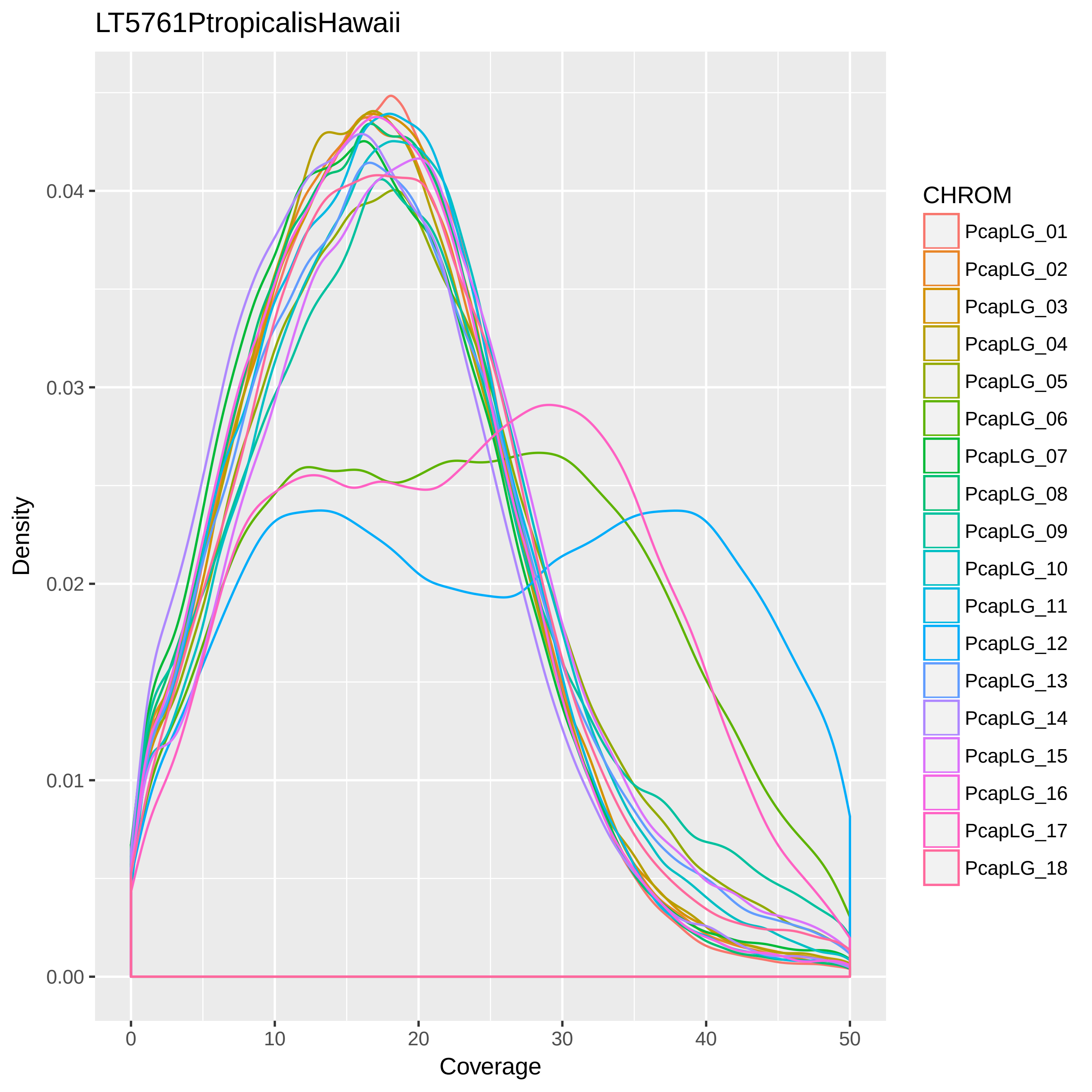
LT5761PtropicalisHawaii
Density
Coverage
0.00
0.01
0.02
0.03
0.04
0
10
20
30
40
50
CHROM
PcapLG_01
PcapLG_02
PcapLG_03
PcapLG_04
PcapLG_05
PcapLG_06
PcapLG_07
PcapLG_08
PcapLG_09
PcapLG_10
PcapLG_11
PcapLG_12
PcapLG_13
PcapLG_14
PcapLG_15
PcapLG_16
PcapLG_17
PcapLG_18

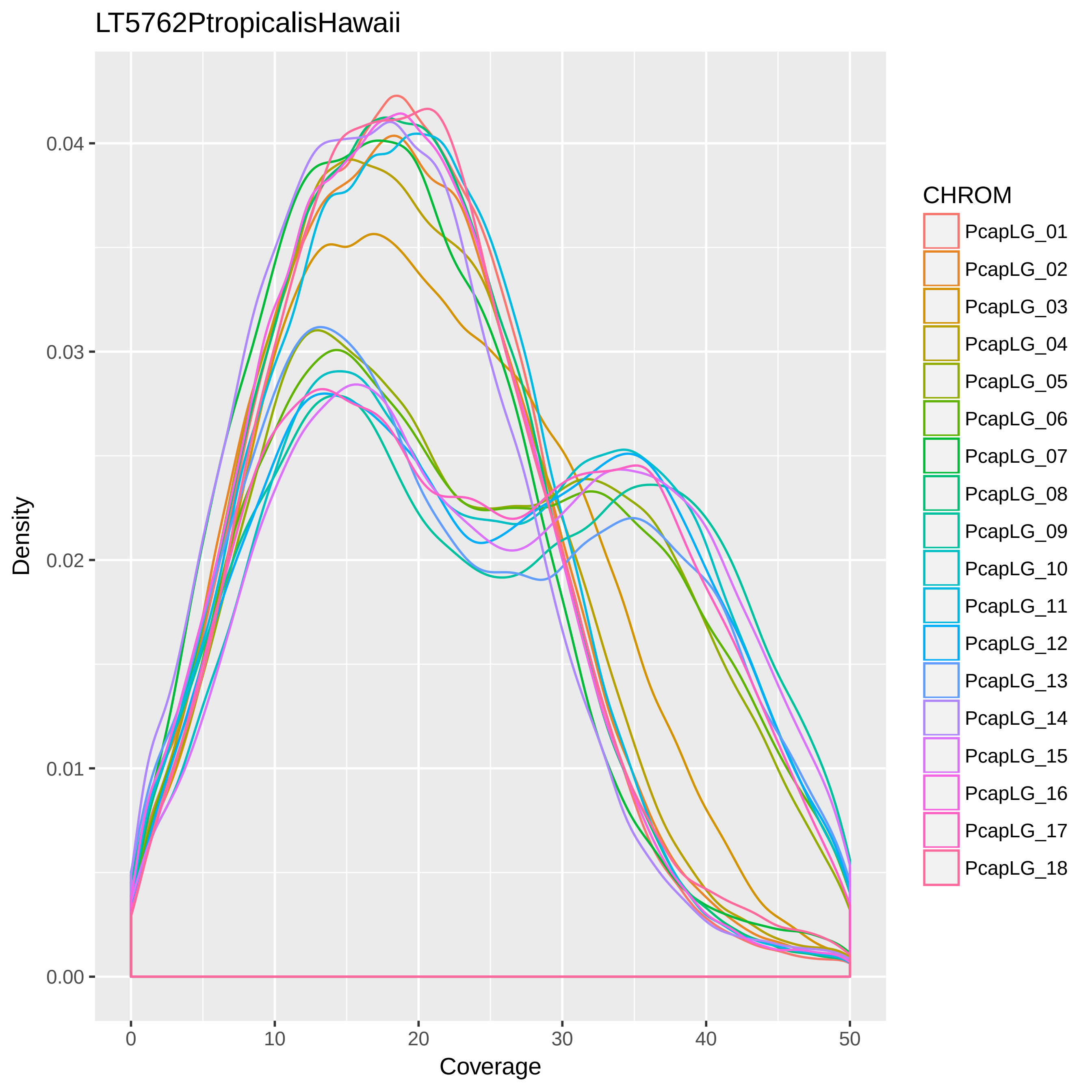
LT5762PtropicalisHawaii
Density
0.00
0.01
0.02
0.03
0.04
0
10
20
30
40
50
Coverage
CHROM
PcapLG_01
PcapLG_02
PcapLG_03
PcapLG_04
PcapLG_05
PcapLG_06
PcapLG_07
PcapLG_08
PcapLG_09
PcapLG_10
PcapLG_11
PcapLG_12
PcapLG_13
PcapLG_14
PcapLG_15
PcapLG_16
PcapLG_17
PcapLG_18

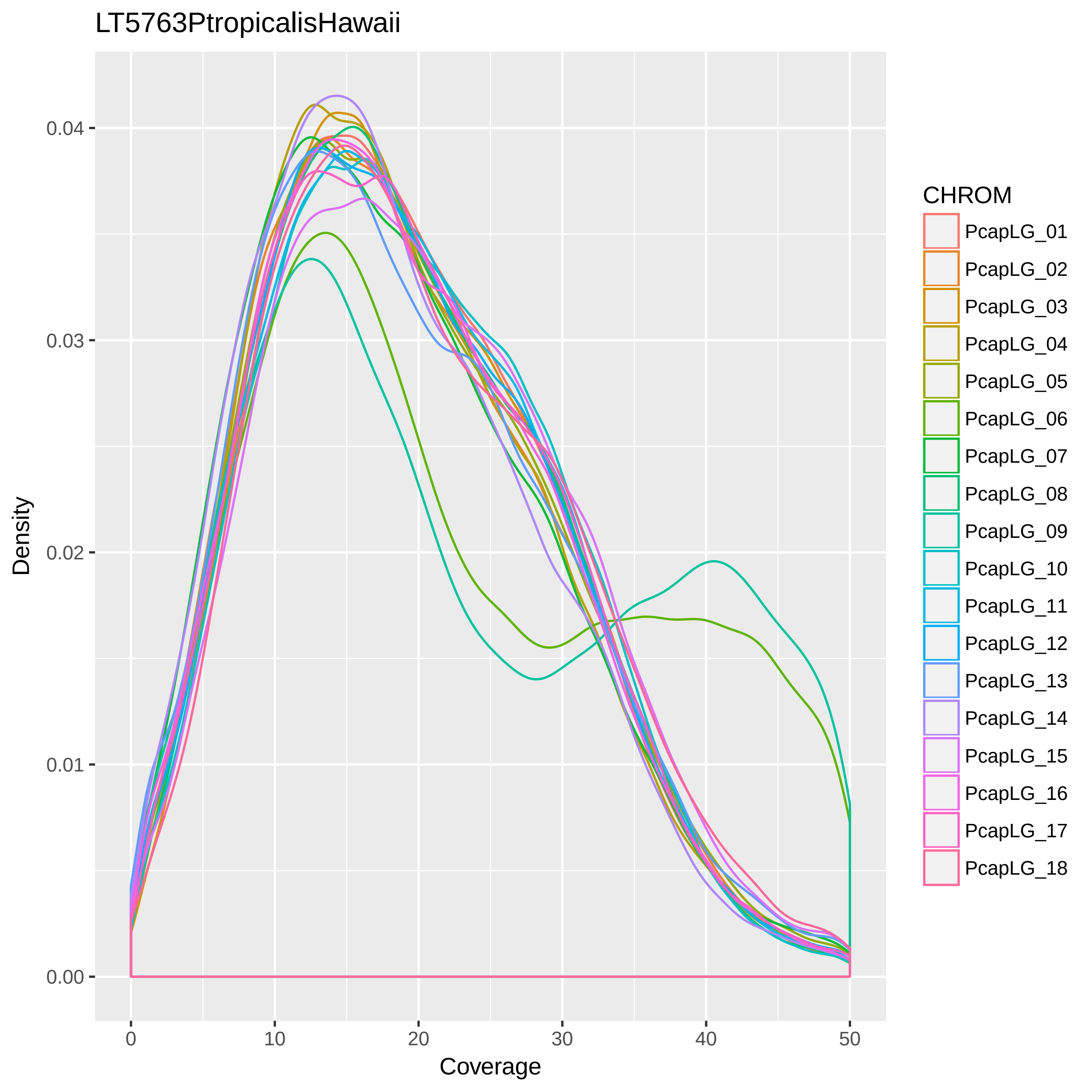

LT5763PtropicalisHawaii
Density
0.00
0.01
0.02
0.03
0.04
0
10
20
30
40
50
Coverage
CHROM
PcapLG_01
PcapLG_02
PcapLG_03
PcapLG_04
PcapLG_05
PcapLG_06
PcapLG_07
PcapLG_08
PcapLG_09
PcapLG_10
PcapLG_11
PcapLG_12
PcapLG_13
PcapLG_14
PcapLG_15
PcapLG_16
PcapLG_17
PcapLG_18

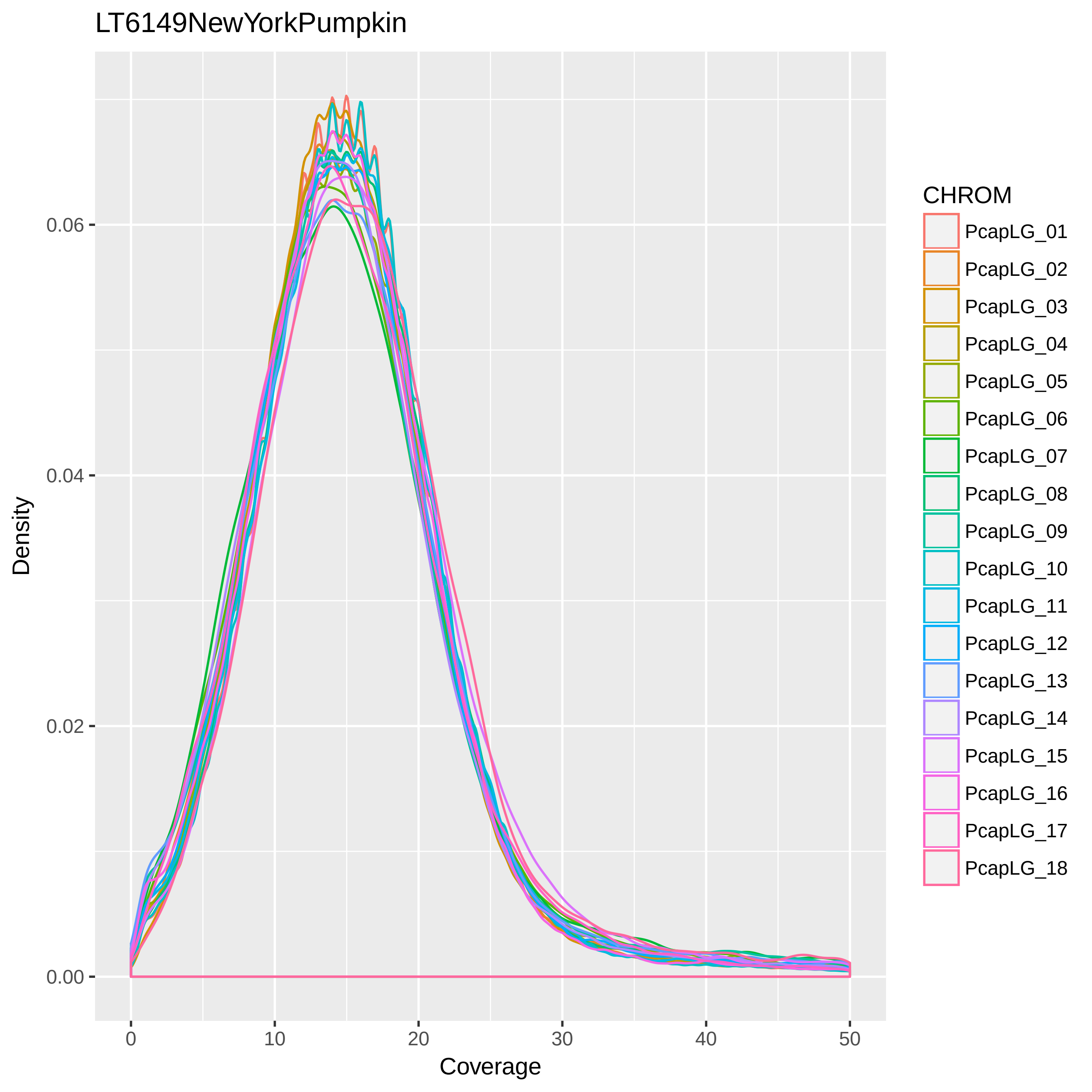
LT6149NewYorkPumpkin
Density
Coverage
0.00
0.02
0.04
0.06
0
10
20
30
40
50
CHROM
PcapLG_01
PcapLG_02
PcapLG_03
PcapLG_04
PcapLG_05
PcapLG_06
PcapLG_07
PcapLG_08
PcapLG_09
PcapLG_10
PcapLG_11
PcapLG_12
PcapLG_13
PcapLG_14
PcapLG_15
PcapLG_16
PcapLG_17
PcapLG_18

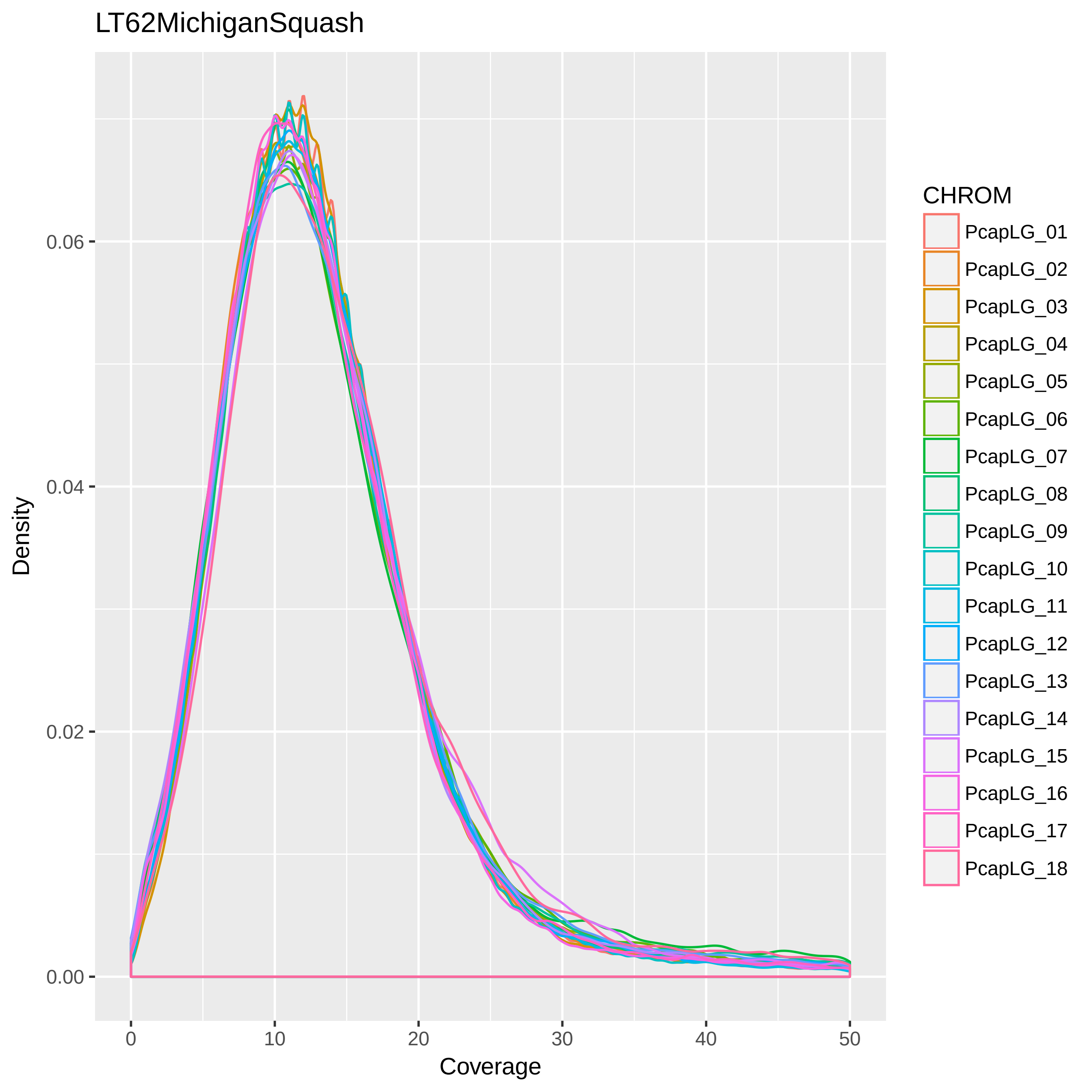

LT62MichiganSquash
Density
Coverage
0.00
0.02
0.04
0.06
0
10
20
30
40
50
CHROM
PcapLG_01
PcapLG_02
PcapLG_03
PcapLG_04
PcapLG_05
PcapLG_06
PcapLG_07
PcapLG_08
PcapLG_09
PcapLG_10
PcapLG_11
PcapLG_12
PcapLG_13
PcapLG_14
PcapLG_15
PcapLG_16
PcapLG_17
PcapLG_18

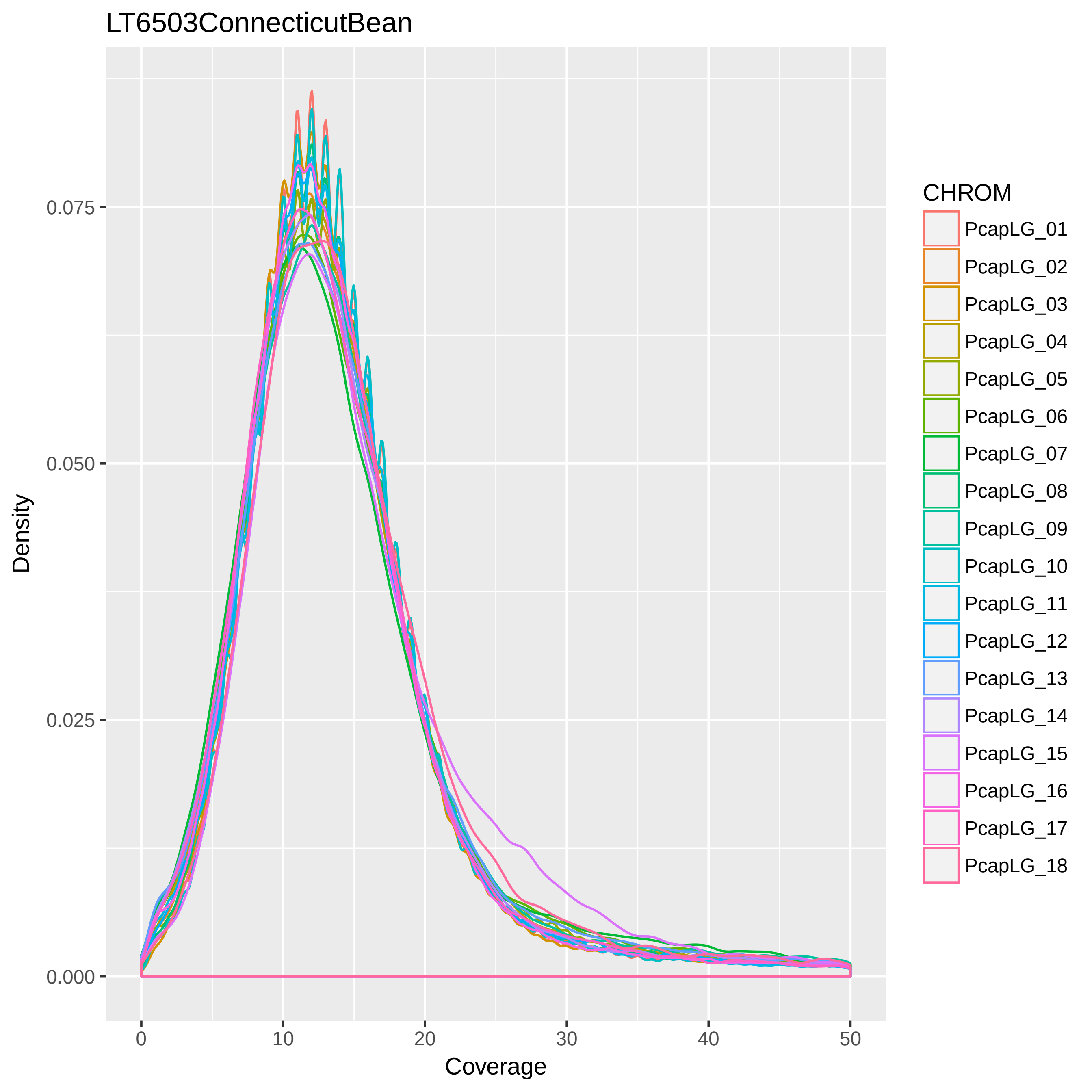

LT6503ConnecticutBean
Density
0.000
0.025
0.050
0.075
0
10
20
30
40
50
Coverage
CHROM
PcapLG_01
PcapLG_02
PcapLG_03
PcapLG_04
PcapLG_05
PcapLG_06
PcapLG_07
PcapLG_08
PcapLG_09
PcapLG_10
PcapLG_11
PcapLG_12
PcapLG_13
PcapLG_14
PcapLG_15
PcapLG_16
PcapLG_17
PcapLG_18

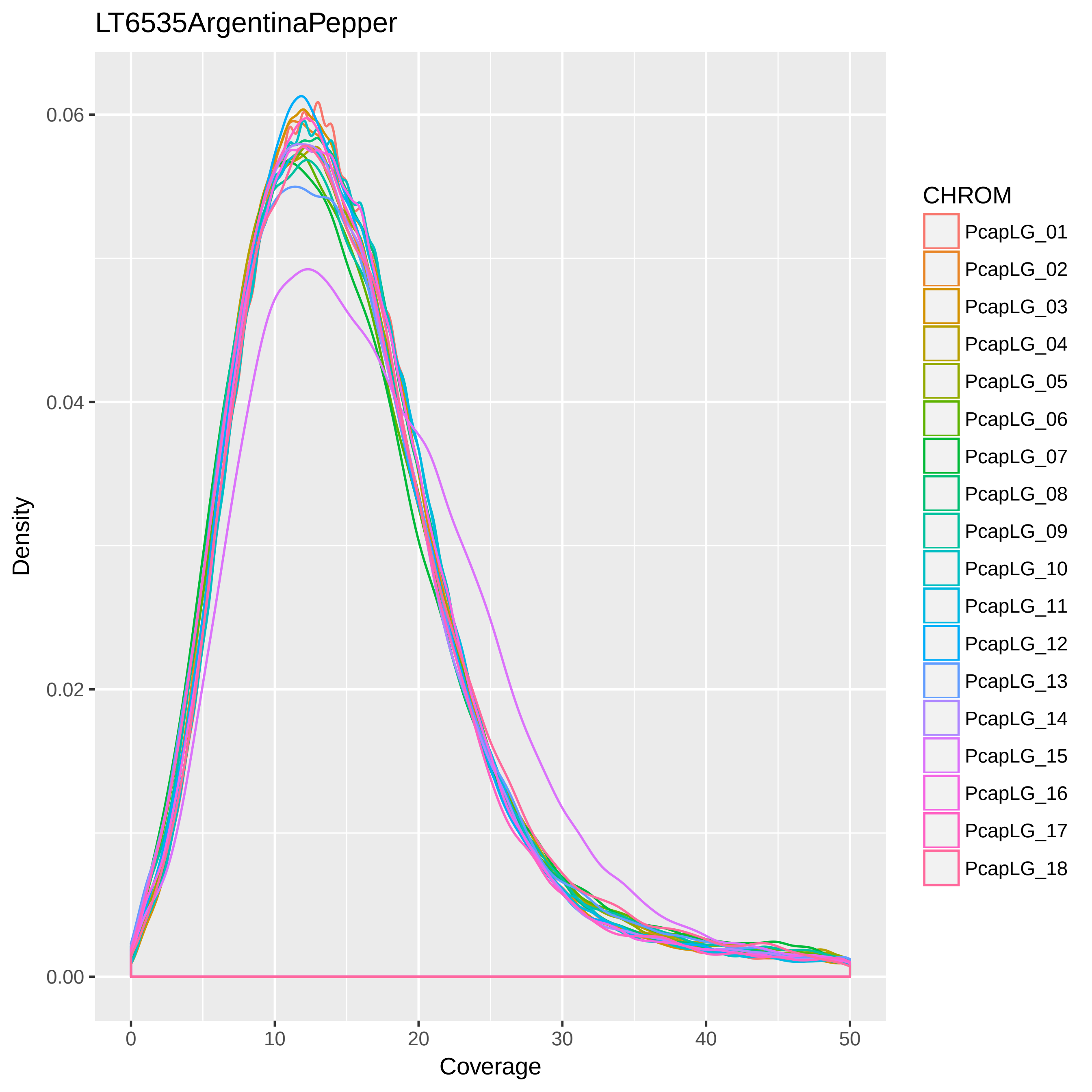

LT6535ArgentinaPepper
Density
Coverage
0.00
0.02
0.04
0.06
0
10
20
30
40
50
CHROM
PcapLG_01
PcapLG_02
PcapLG_03
PcapLG_04
PcapLG_05
PcapLG_06
PcapLG_07
PcapLG_08
PcapLG_09
PcapLG_10
PcapLG_11
PcapLG_12
PcapLG_13
PcapLG_14
PcapLG_15
PcapLG_16
PcapLG_17
PcapLG_18

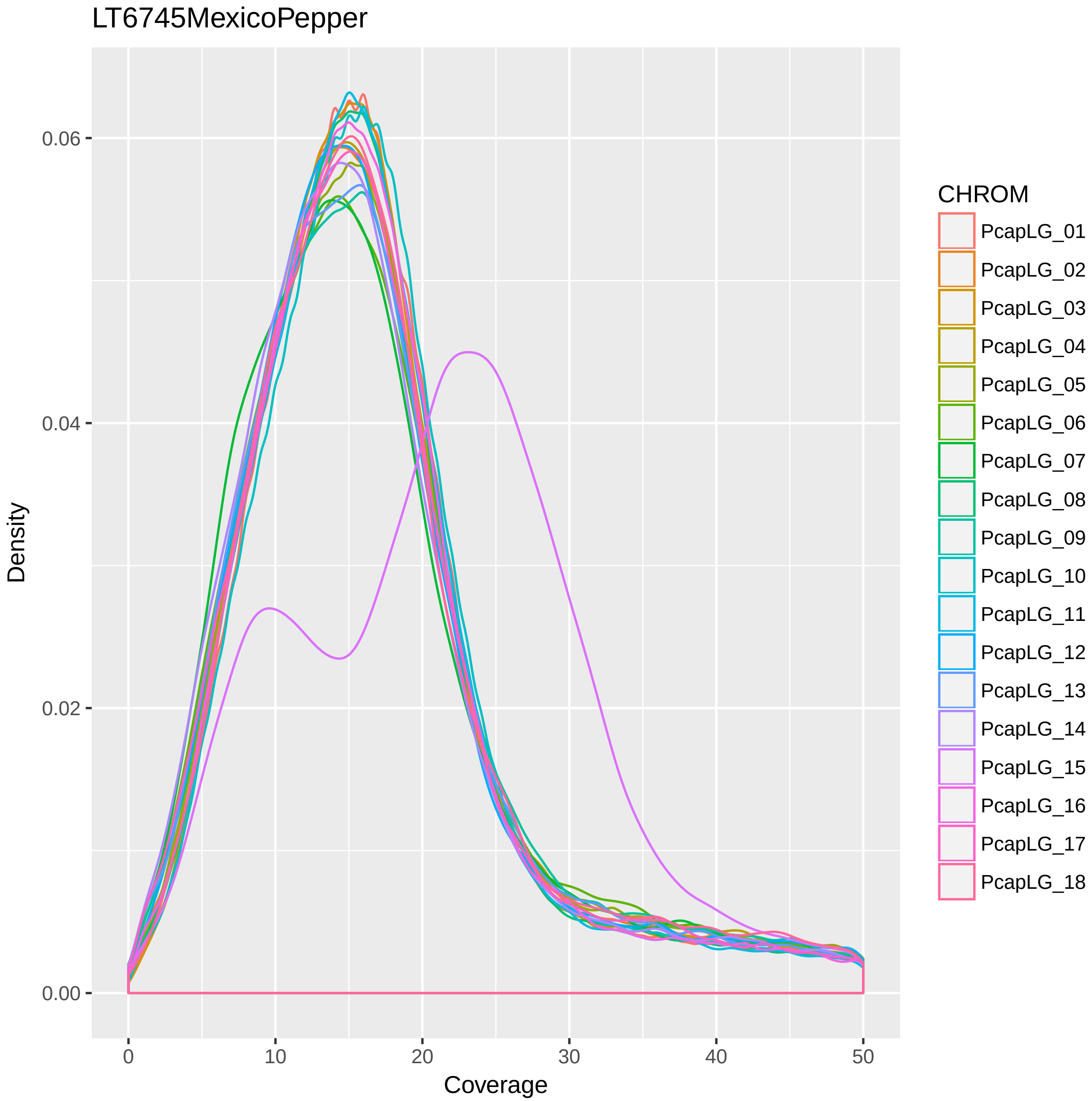

LT6745MexicoPepper
Density
0.00
0.02
0.04
0.06
Coverage
0
10
20
30
40
50
CHROM
PcapLG_01
PcapLG_02
PcapLG_03
PcapLG_04
PcapLG_05
PcapLG_06
PcapLG_07
PcapLG_08
PcapLG_09
PcapLG_10
PcapLG_11
PcapLG_12
PcapLG_13
PcapLG_14
PcapLG_15
PcapLG_16
PcapLG_17
PcapLG_18

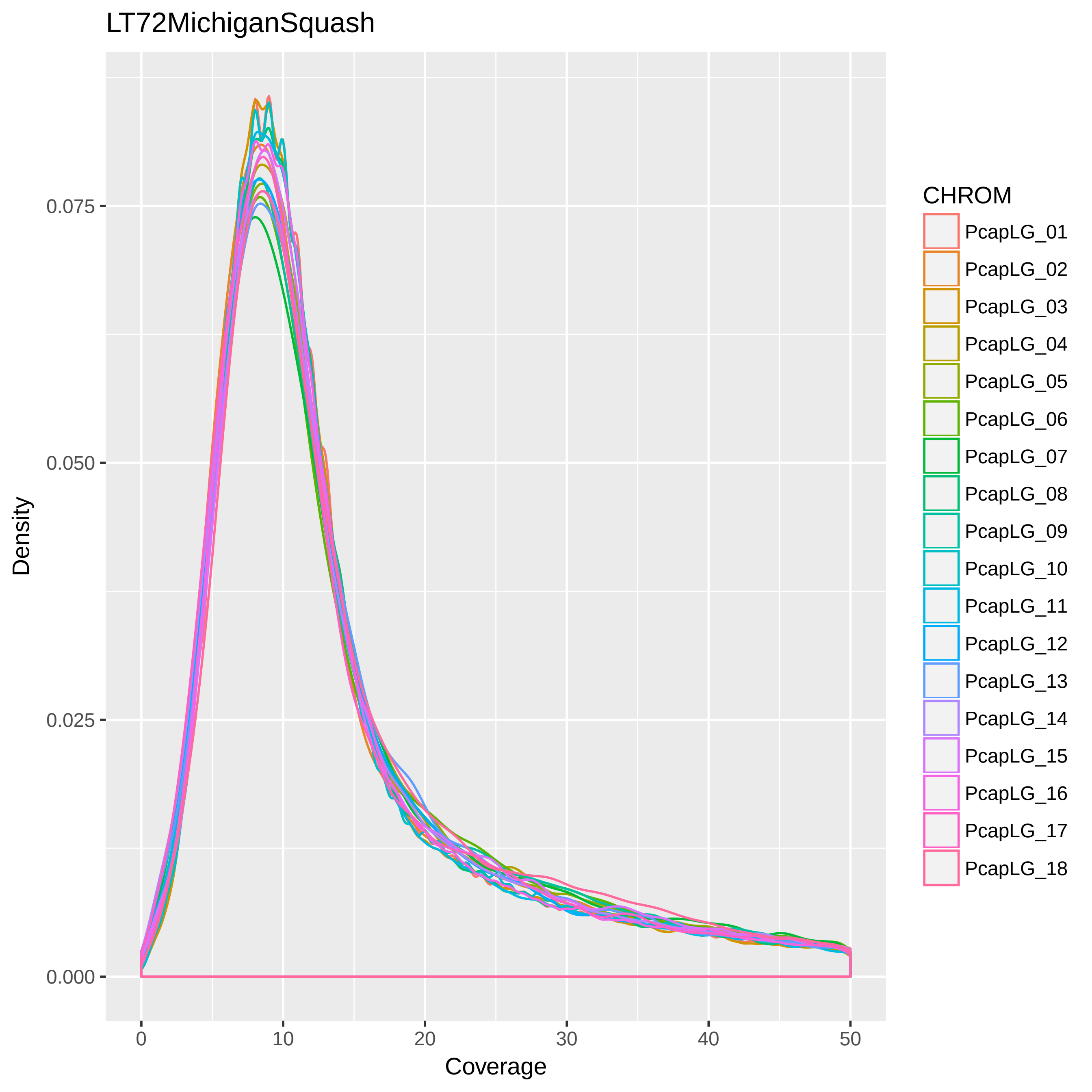

LT72MichiganSquash
Density
Coverage
0.000
0.025
0.050
0.075
0
10
20
30
40
50
CHROM
PcapLG_01
PcapLG_02
PcapLG_03
PcapLG_04
PcapLG_05
PcapLG_06
PcapLG_07
PcapLG_08
PcapLG_09
PcapLG_10
PcapLG_11
PcapLG_12
PcapLG_13
PcapLG_14
PcapLG_15
PcapLG_16
PcapLG_17
PcapLG_18

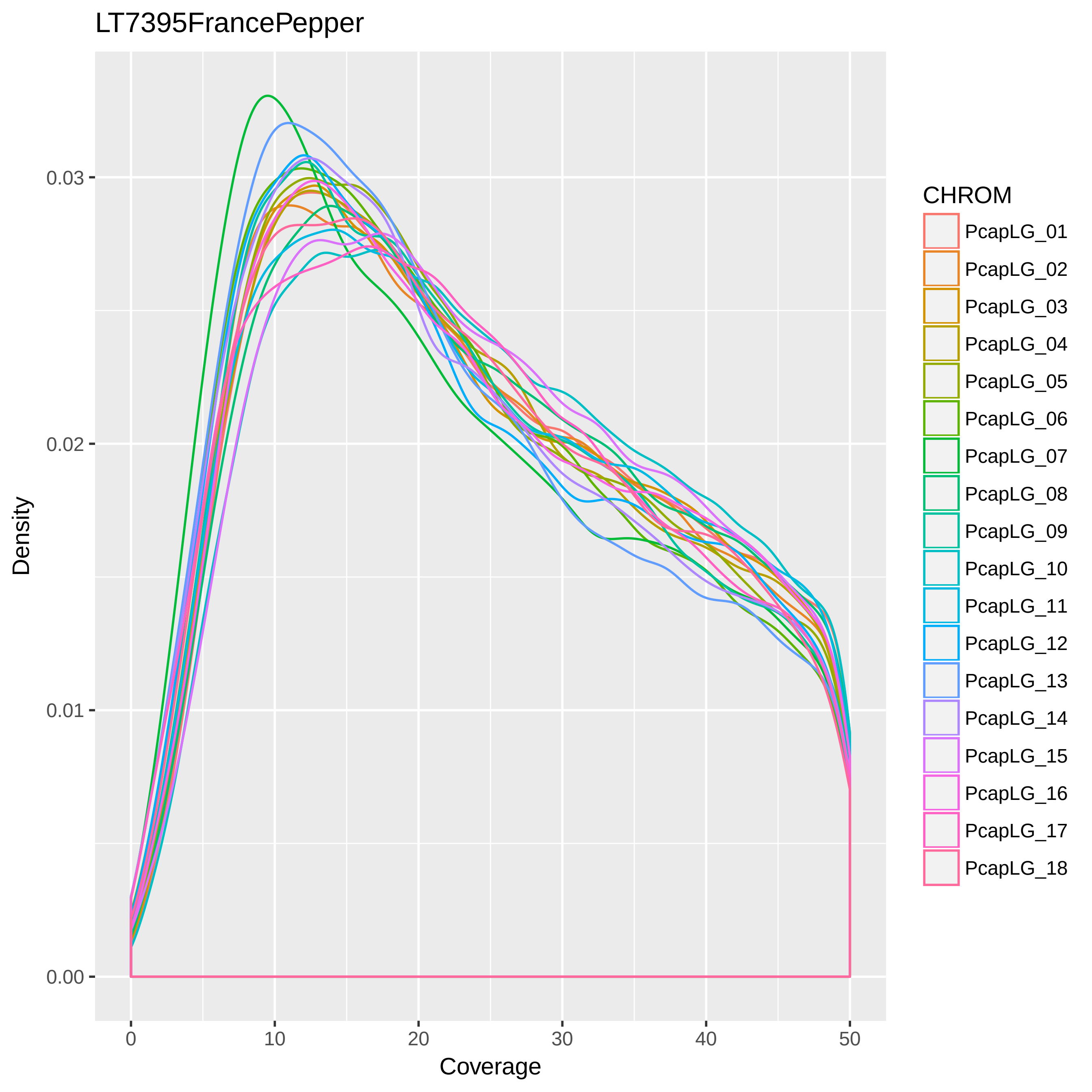
LT7395FrancePepper
Density
Coverage
0.00
0.01
0.02
0.03
0
10
20
30
40
50
CHROM
PcapLG_01
PcapLG_02
PcapLG_03
PcapLG_04
PcapLG_05
PcapLG_06
PcapLG_07
PcapLG_08
PcapLG_09
PcapLG_10
PcapLG_11
PcapLG_12
PcapLG_13
PcapLG_14
PcapLG_15
PcapLG_16
PcapLG_17
PcapLG_18

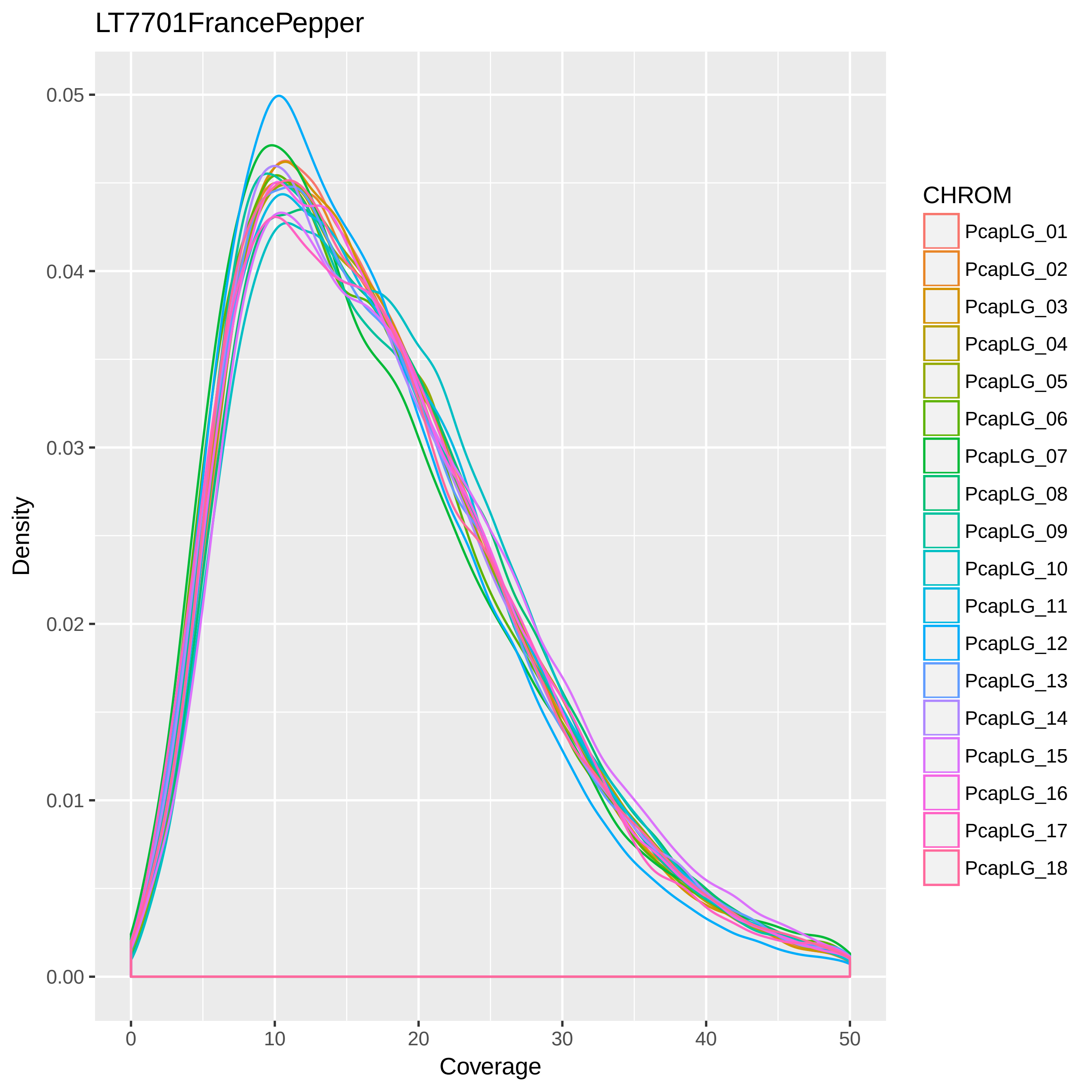

LT7701FrancePepper
Density
Coverage
0.00
0.01
0.02
0.03
0.04
0.05
0
10
20
30
40
50
CHROM
PcapLG_01
PcapLG_02
PcapLG_03
PcapLG_04
PcapLG_05
PcapLG_06
PcapLG_07
PcapLG_08
PcapLG_09
PcapLG_10
PcapLG_11
PcapLG_12
PcapLG_13
PcapLG_14
PcapLG_15
PcapLG_16
PcapLG_17
PcapLG_18

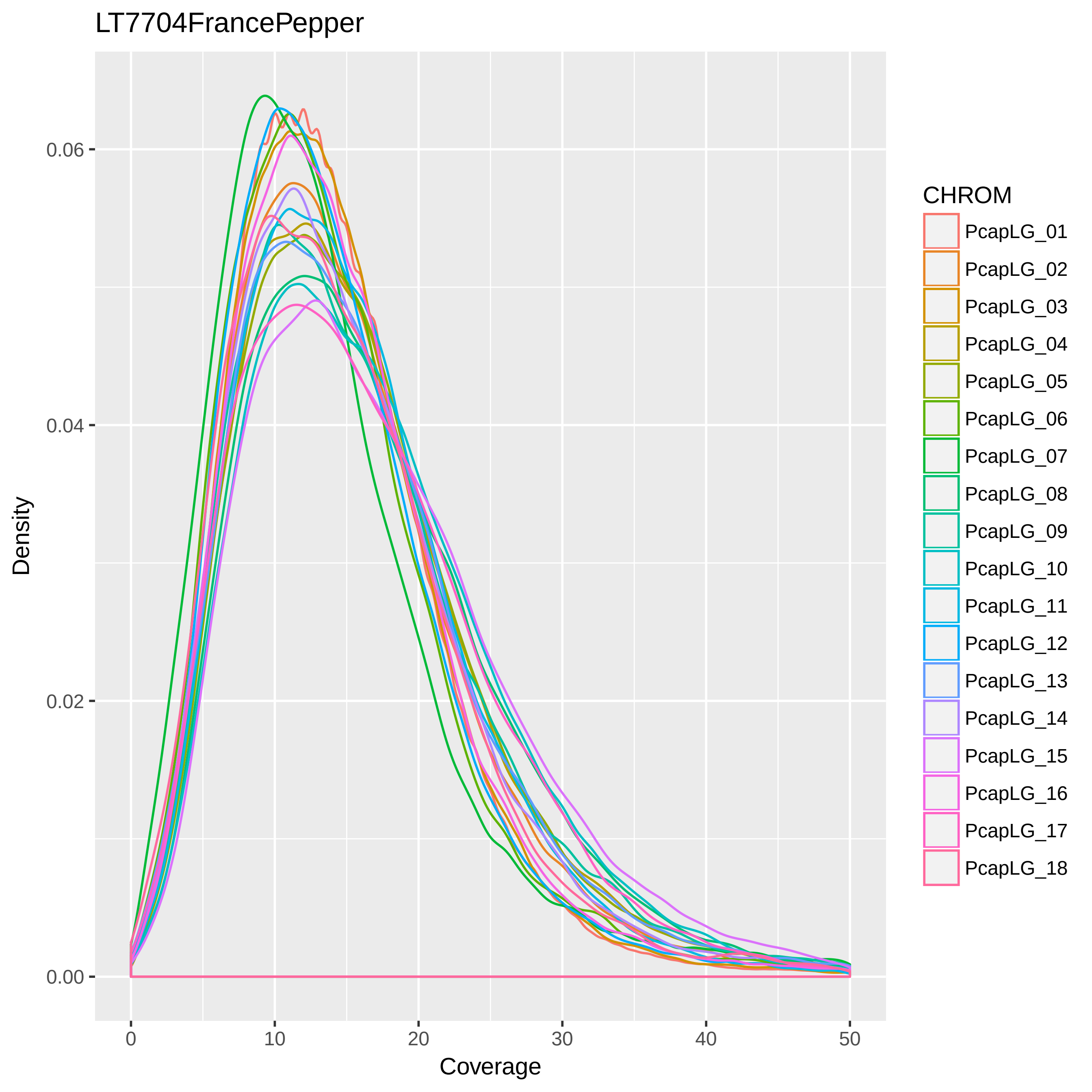

LT7704FrancePepper
Density
Coverage
0.00
0.02
0.04
0.06
0
10
20
30
40
50
CHROM
PcapLG_01
PcapLG_02
PcapLG_03
PcapLG_04
PcapLG_05
PcapLG_06
PcapLG_07
PcapLG_08
PcapLG_09
PcapLG_10
PcapLG_11
PcapLG_12
PcapLG_13
PcapLG_14
PcapLG_15
PcapLG_16
PcapLG_17
PcapLG_18

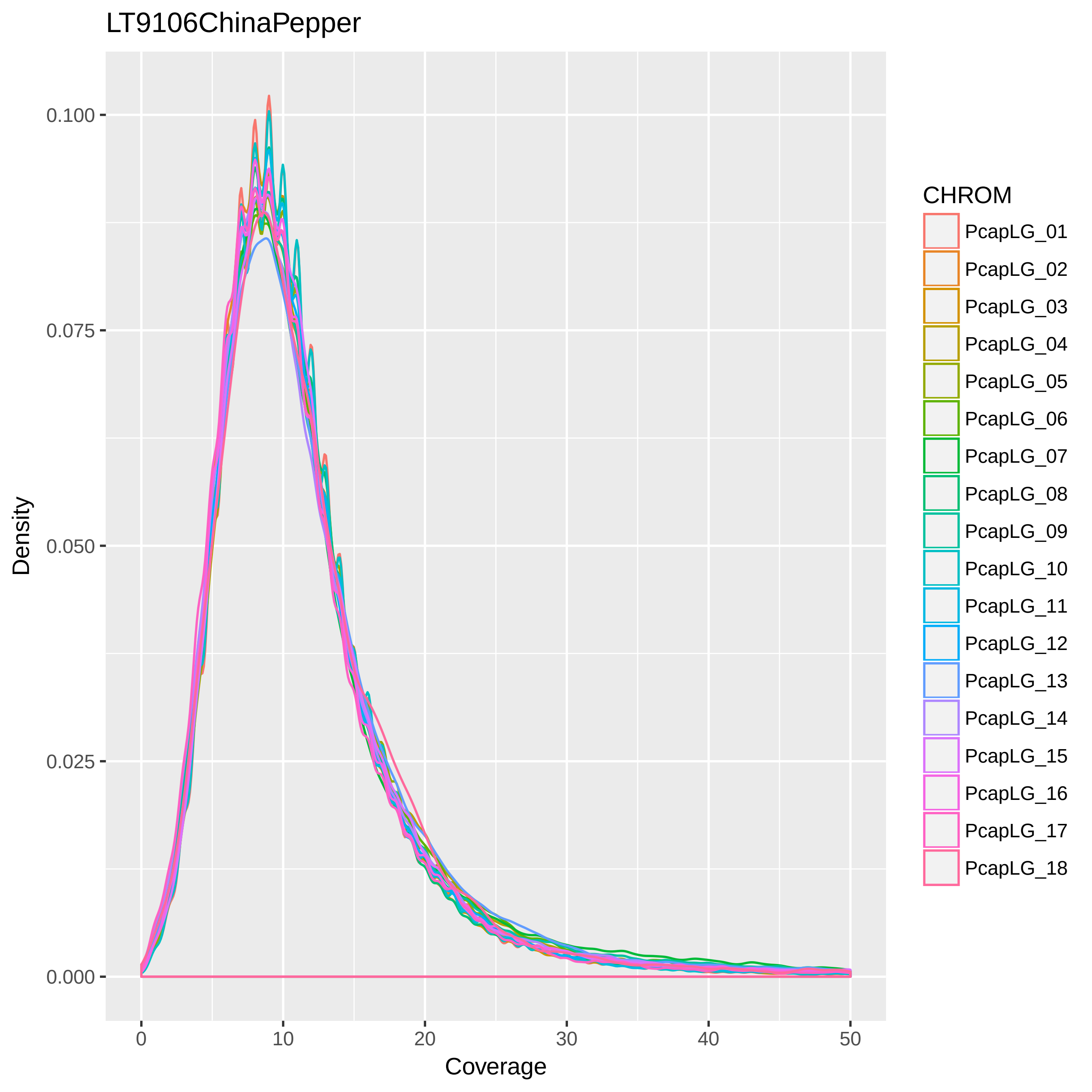
LT9106ChinaPepper
Density
0.100
0.075
0.050
0.025
0.000
0
10
20
30
40
50
Coverage
CHROM
PcapLG_01
PcapLG_02
PcapLG_03
PcapLG_04
PcapLG_05
PcapLG_06
PcapLG_07
PcapLG_08
PcapLG_09
PcapLG_10
PcapLG_11
PcapLG_12
PcapLG_13
PcapLG_14
PcapLG_15
PcapLG_16
PcapLG_17
PcapLG_18

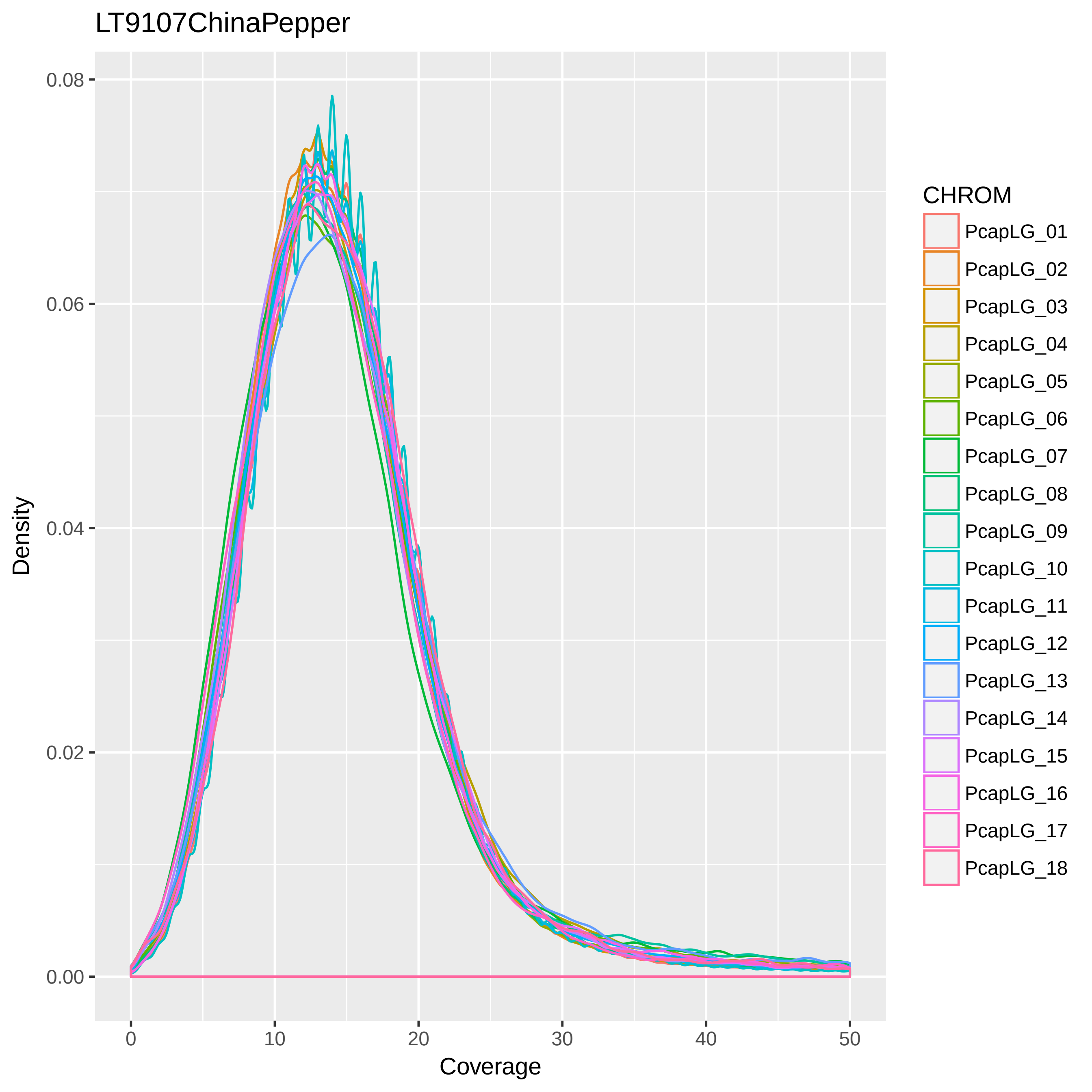
LT9107ChinaPepper
Density
0.00
0.02
0.04
0.06
0.08
0
10
20
30
40
50
Coverage
CHROM
PcapLG_01
PcapLG_02
PcapLG_03
PcapLG_04
PcapLG_05
PcapLG_06
PcapLG_07
PcapLG_08
PcapLG_09
PcapLG_10
PcapLG_11
PcapLG_12
PcapLG_13
PcapLG_14
PcapLG_15
PcapLG_16
PcapLG_17
PcapLG_18

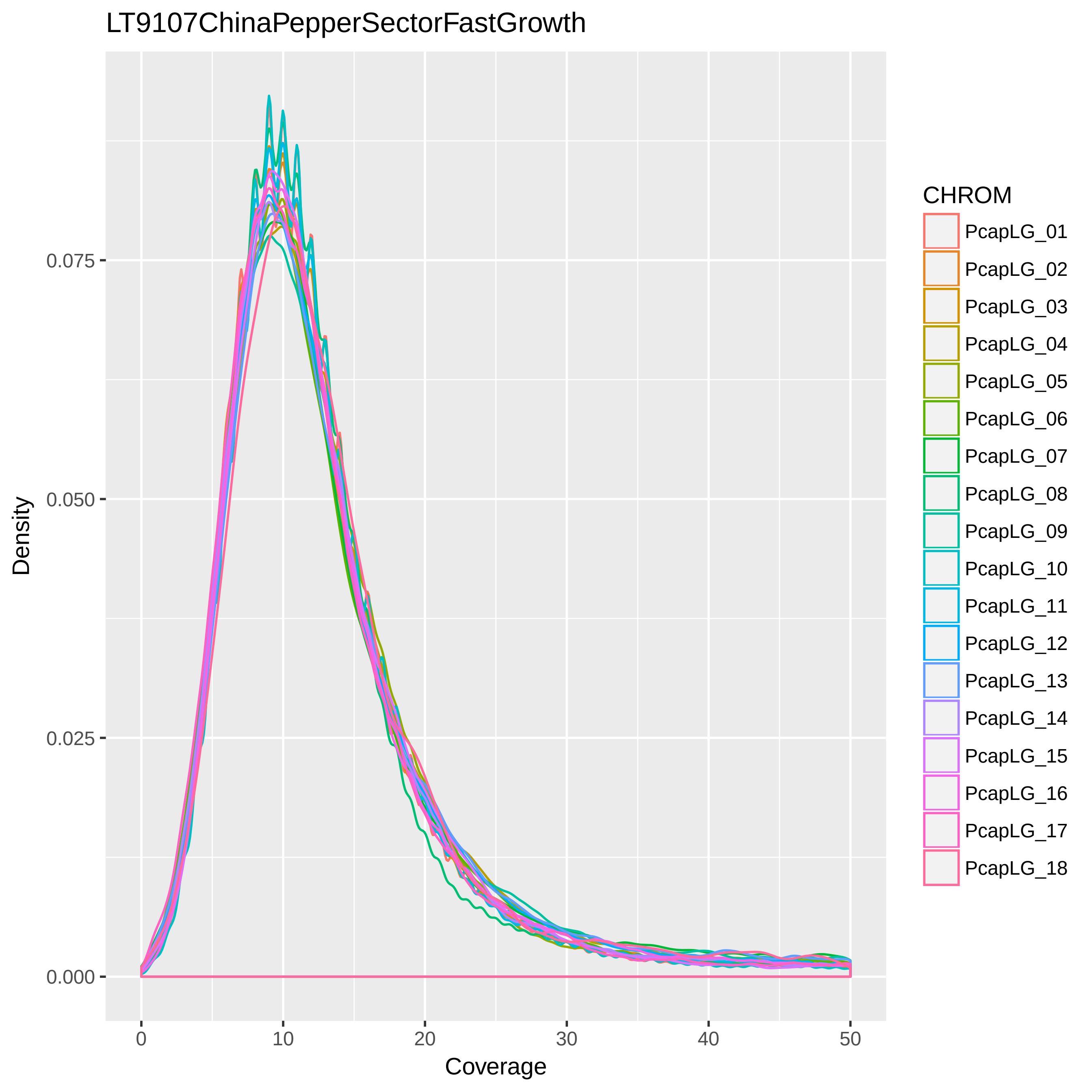
LT9107ChinaPepperSectorFastGrowth
Density
0.000
0.025
0.050
0.075
0
10
20
30
40
50
Coverage
CHROM
PcapLG_01
PcapLG_02
PcapLG_03
PcapLG_04
PcapLG_05
PcapLG_06
PcapLG_07
PcapLG_08
PcapLG_09
PcapLG_10
PcapLG_11
PcapLG_12
PcapLG_13
PcapLG_14
PcapLG_15
PcapLG_16
PcapLG_17
PcapLG_18

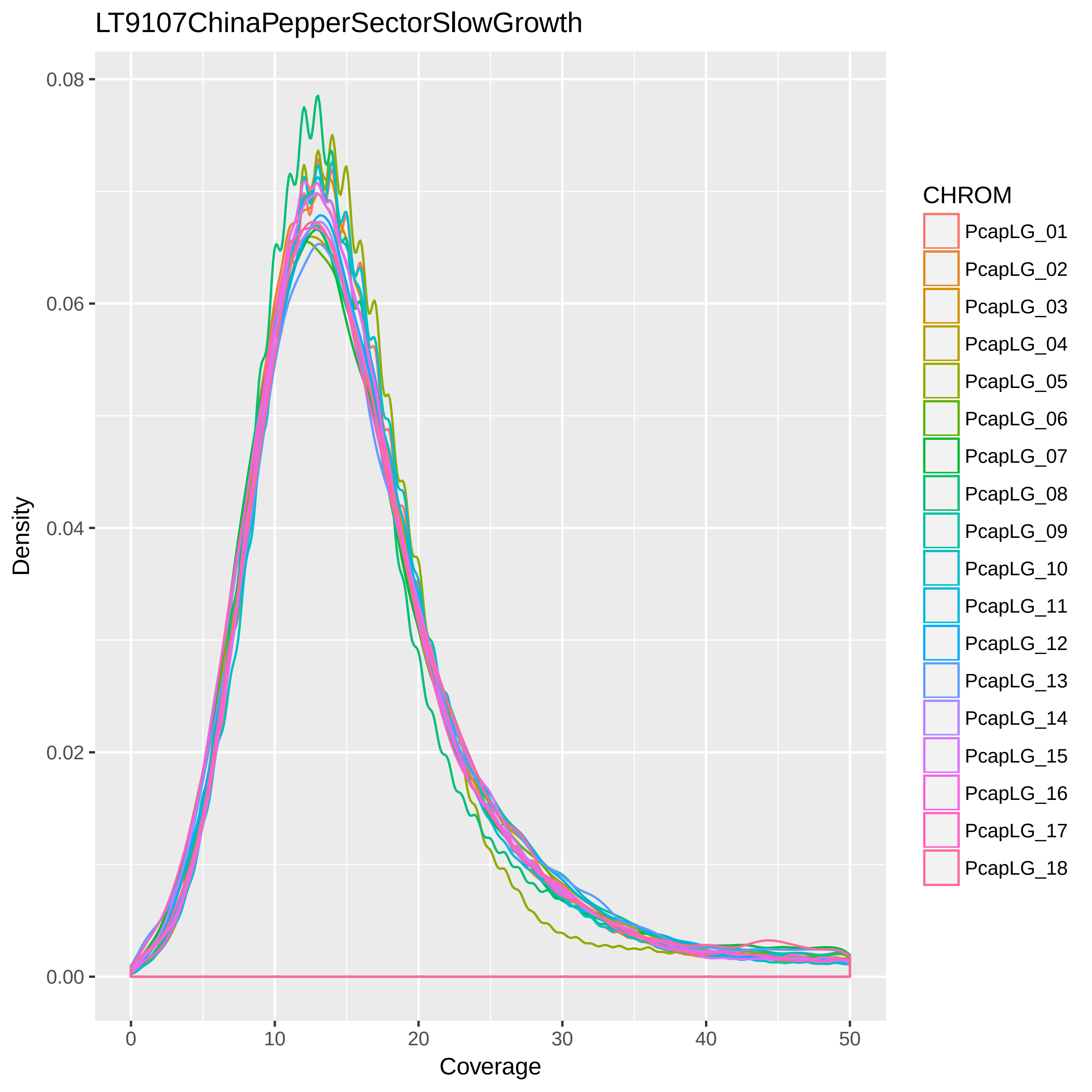

LT9107ChinaPepperSectorSlowGrowth
Density
0.00
0.02
0.04
0.06
0.08
0
10
20
30
40
50
Coverage
CHROM
PcapLG_01
PcapLG_02
PcapLG_03
PcapLG_04
PcapLG_05
PcapLG_06
PcapLG_07
PcapLG_08
PcapLG_09
PcapLG_10
PcapLG_11
PcapLG_12
PcapLG_13
PcapLG_14
PcapLG_15
PcapLG_16
PcapLG_17
PcapLG_18

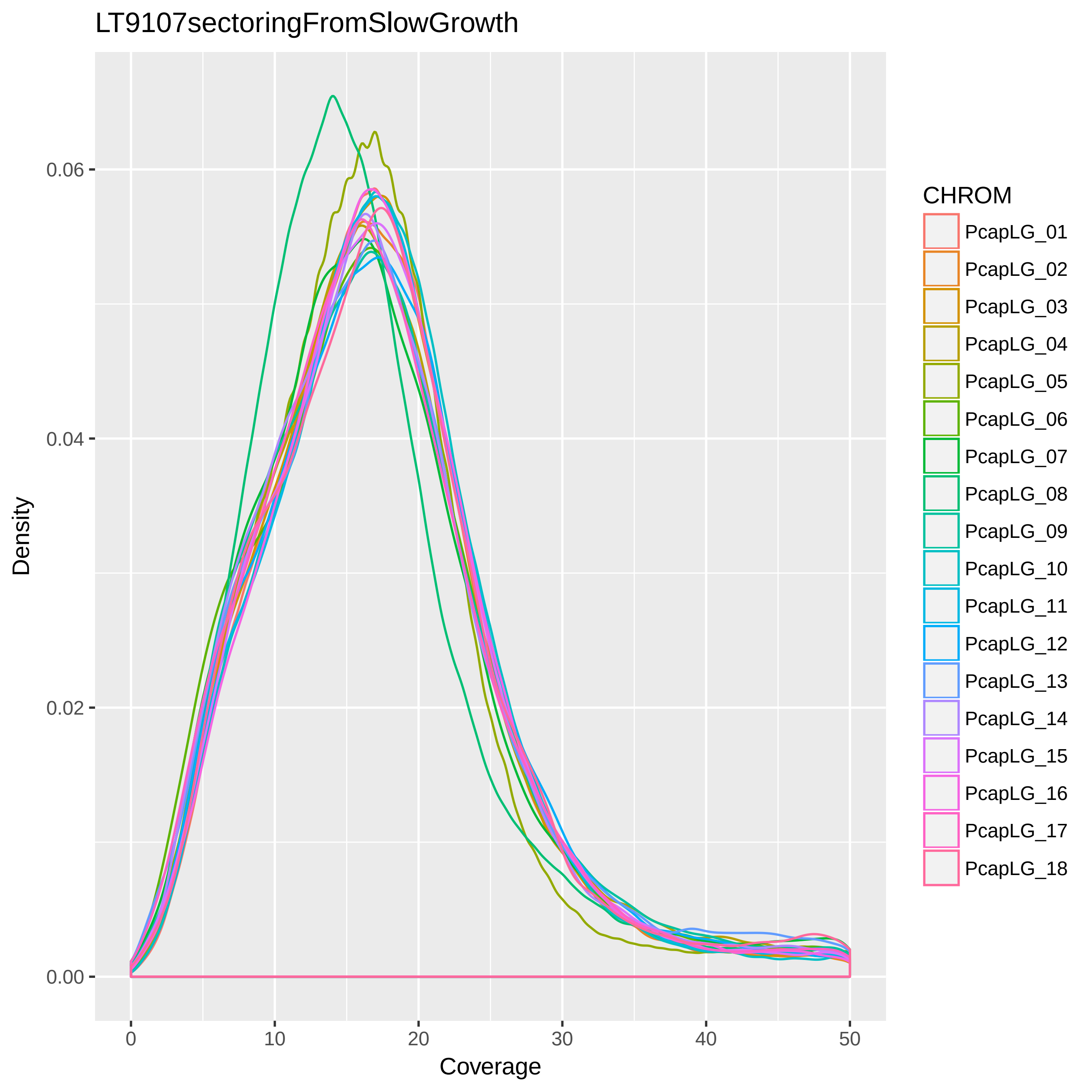

LT9107sectoringFromSlowGrowth
Density
0.06
0.04
0.02
0.00
0
10
20
30
40
50
Coverage
CHROM
PcapLG_01
PcapLG_02
PcapLG_03
PcapLG_04
PcapLG_05
PcapLG_06
PcapLG_07
PcapLG_08
PcapLG_09
PcapLG_10
PcapLG_11
PcapLG_12
PcapLG_13
PcapLG_14
PcapLG_15
PcapLG_16
PcapLG_17
PcapLG_18

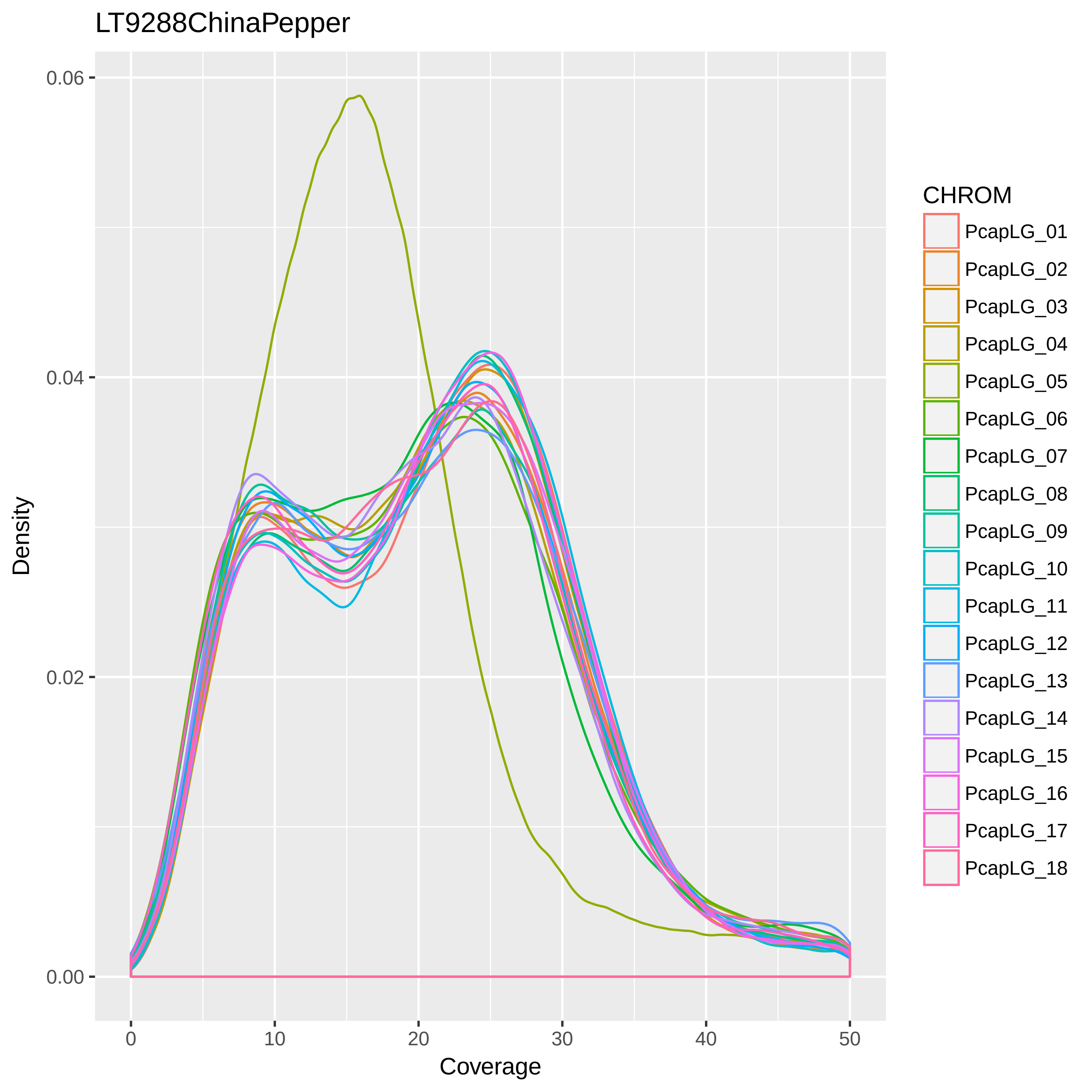

LT9288ChinaPepper
0.06
0.04
0.02
0.00
Density
0
10
20
30
40
50
Coverage
CHROM
PcapLG_01
PcapLG_02
PcapLG_03
PcapLG_04
PcapLG_05
PcapLG_06
PcapLG_07
PcapLG_08
PcapLG_09
PcapLG_10
PcapLG_11
PcapLG_12
PcapLG_13
PcapLG_14
PcapLG_15
PcapLG_16
PcapLG_17
PcapLG_18

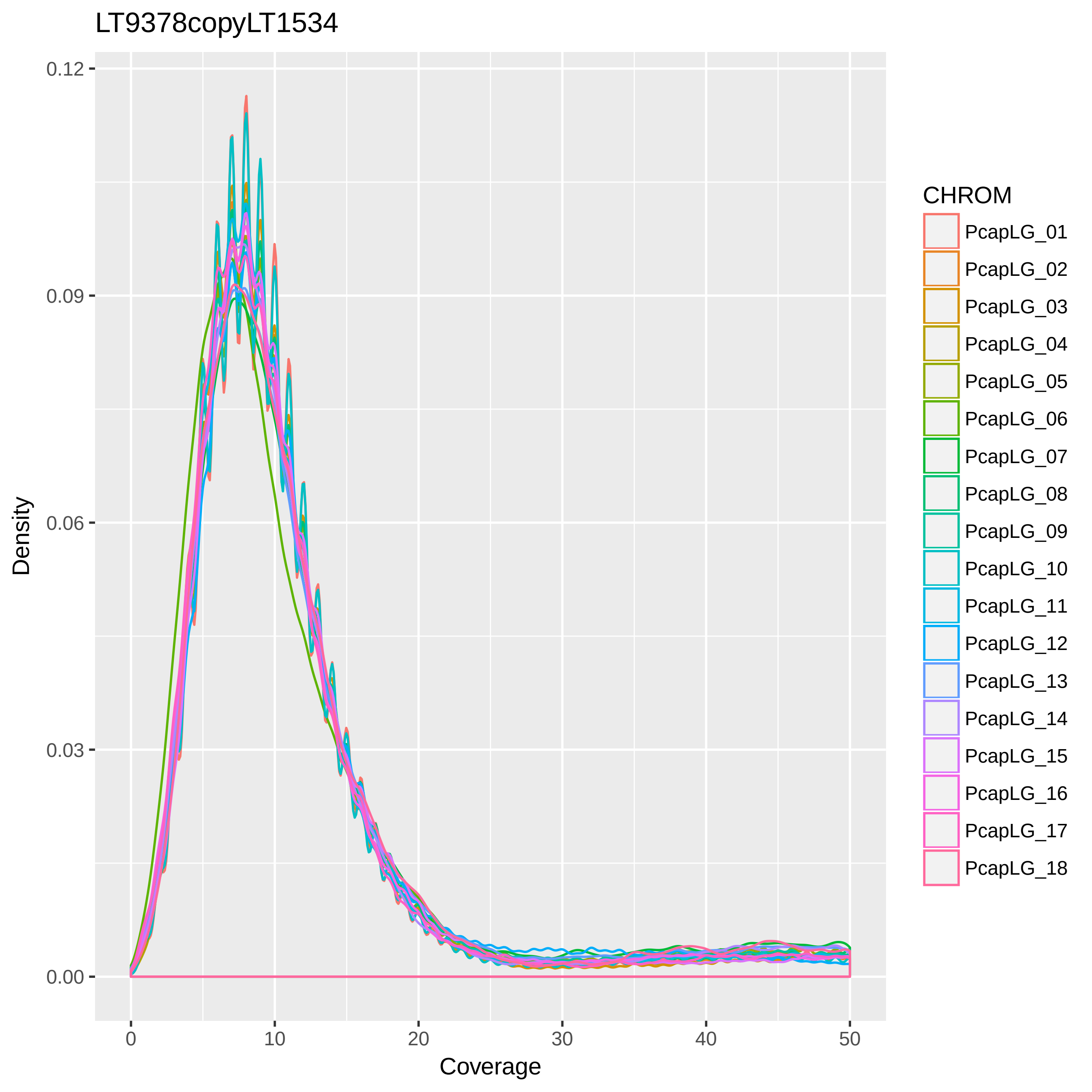

LT9378copyLT1534
Density
Coverage
0.00
0.03
0.06
0.09
0.12
0
10
20
30
40
50
CHROM
PcapLG_01
PcapLG_02
PcapLG_03
PcapLG_04
PcapLG_05
PcapLG_06
PcapLG_07
PcapLG_08
PcapLG_09
PcapLG_10
PcapLG_11
PcapLG_12
PcapLG_13
PcapLG_14
PcapLG_15
PcapLG_16
PcapLG_17
PcapLG_18

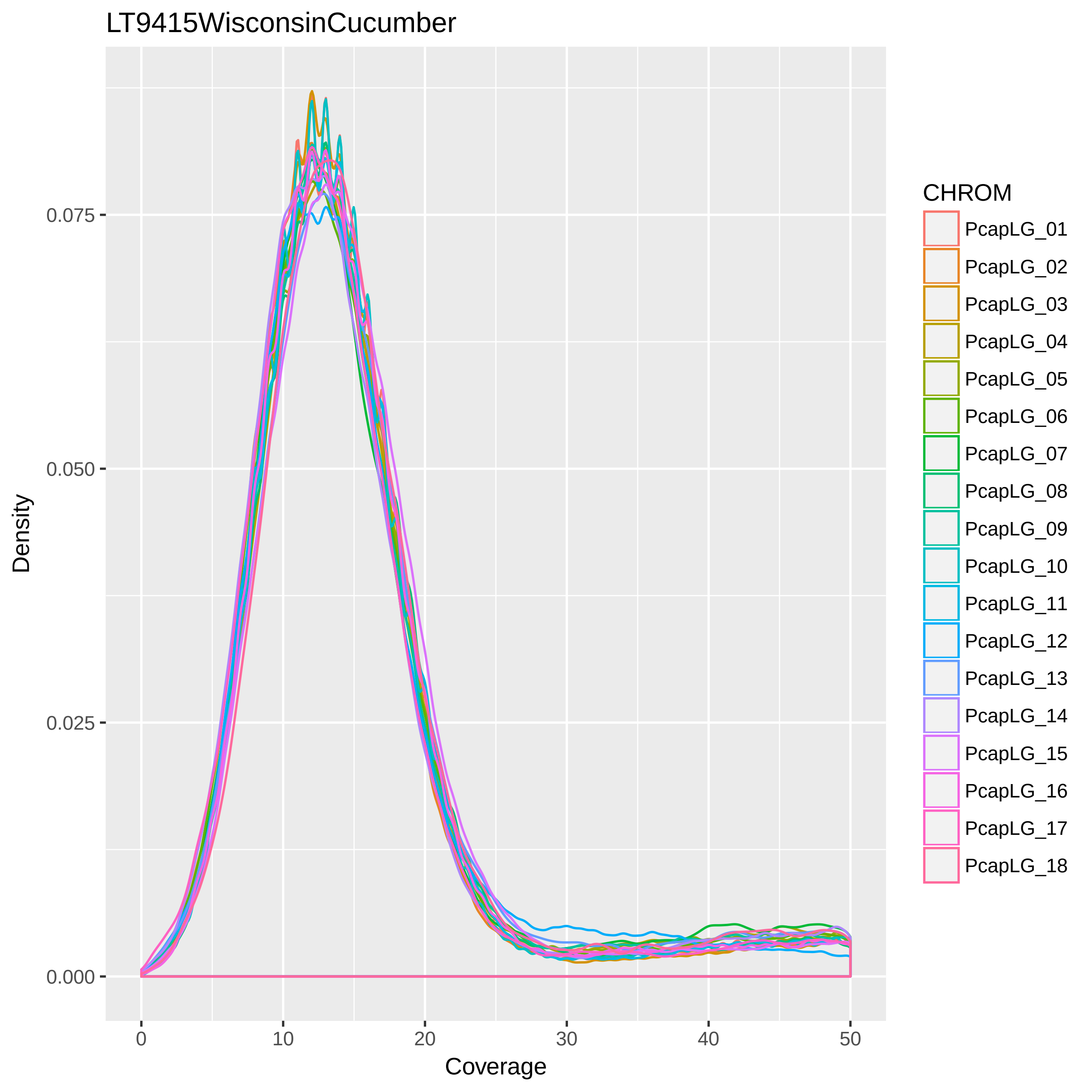
LT9415WisconsinCucumber
Density
Coverage
0.000
0.025
0.050
0.075
0
10
20
30
40
50
CHROM
PcapLG_01
PcapLG_02
PcapLG_03
PcapLG_04
PcapLG_05
PcapLG_06
PcapLG_07
PcapLG_08
PcapLG_09
PcapLG_10
PcapLG_11
PcapLG_12
PcapLG_13
PcapLG_14
PcapLG_15
PcapLG_16
PcapLG_17
PcapLG_18

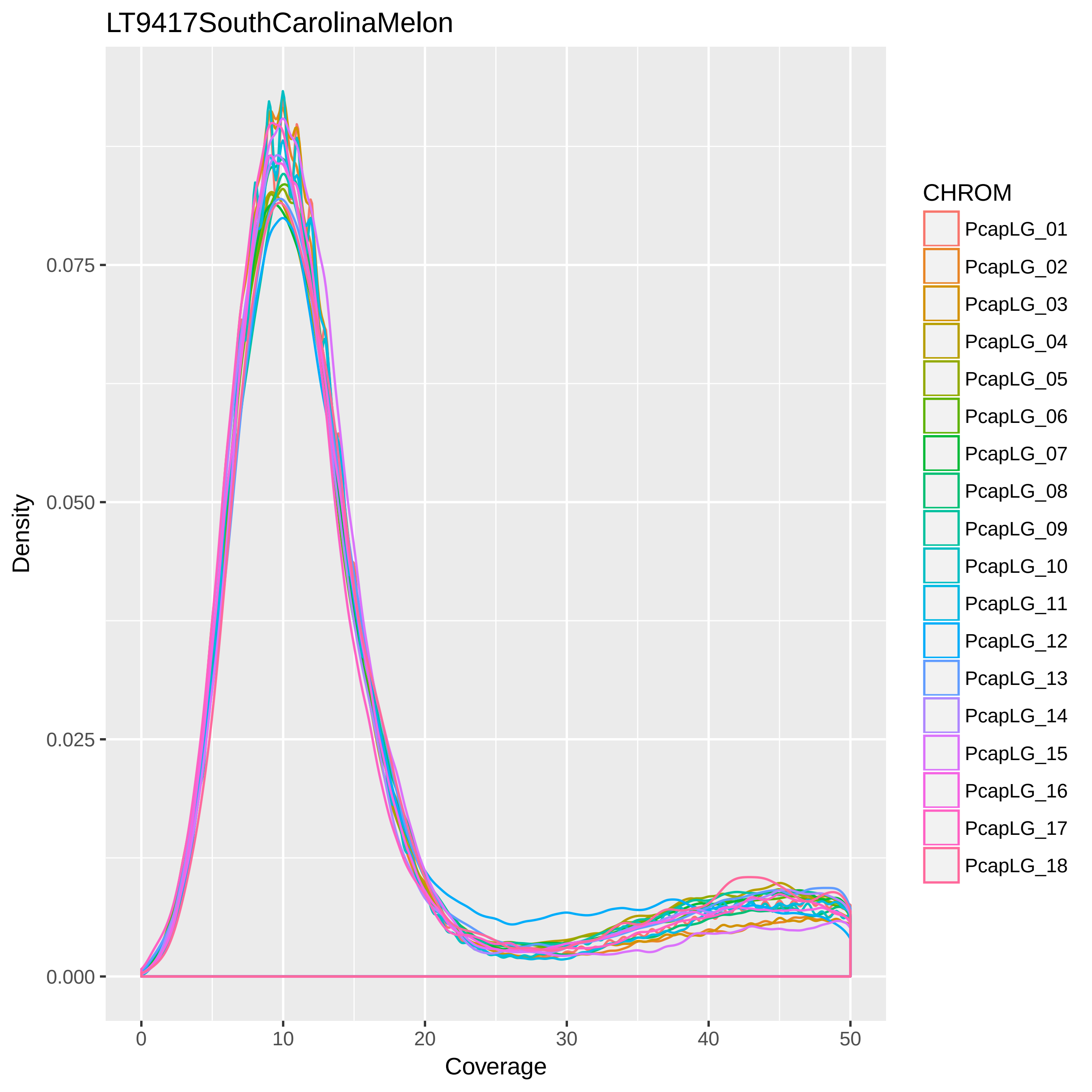

LT9417SouthCarolinaMelon
Density
0.000
0.025
0.050
0.075
0
10
20
30
40
50
Coverage
CHROM
PcapLG_01
PcapLG_02
PcapLG_03
PcapLG_04
PcapLG_05
PcapLG_06
PcapLG_07
PcapLG_08
PcapLG_09
PcapLG_10
PcapLG_11
PcapLG_12
PcapLG_13
PcapLG_14
PcapLG_15
PcapLG_16
PcapLG_17
PcapLG_18
