# Supplementary figures and images for: Dynamic Extreme Aneuploidy (DEA) in the vegetable pathogen Phytophthora capsici and the potential for rapid asexual evolution
Source: PLoS One. 2020 Jan 7;15(1):e0227250. doi: 10.1371/journal.pone.0227250 (PMC6946123; doi:10.1371/journal.pone.0227250)

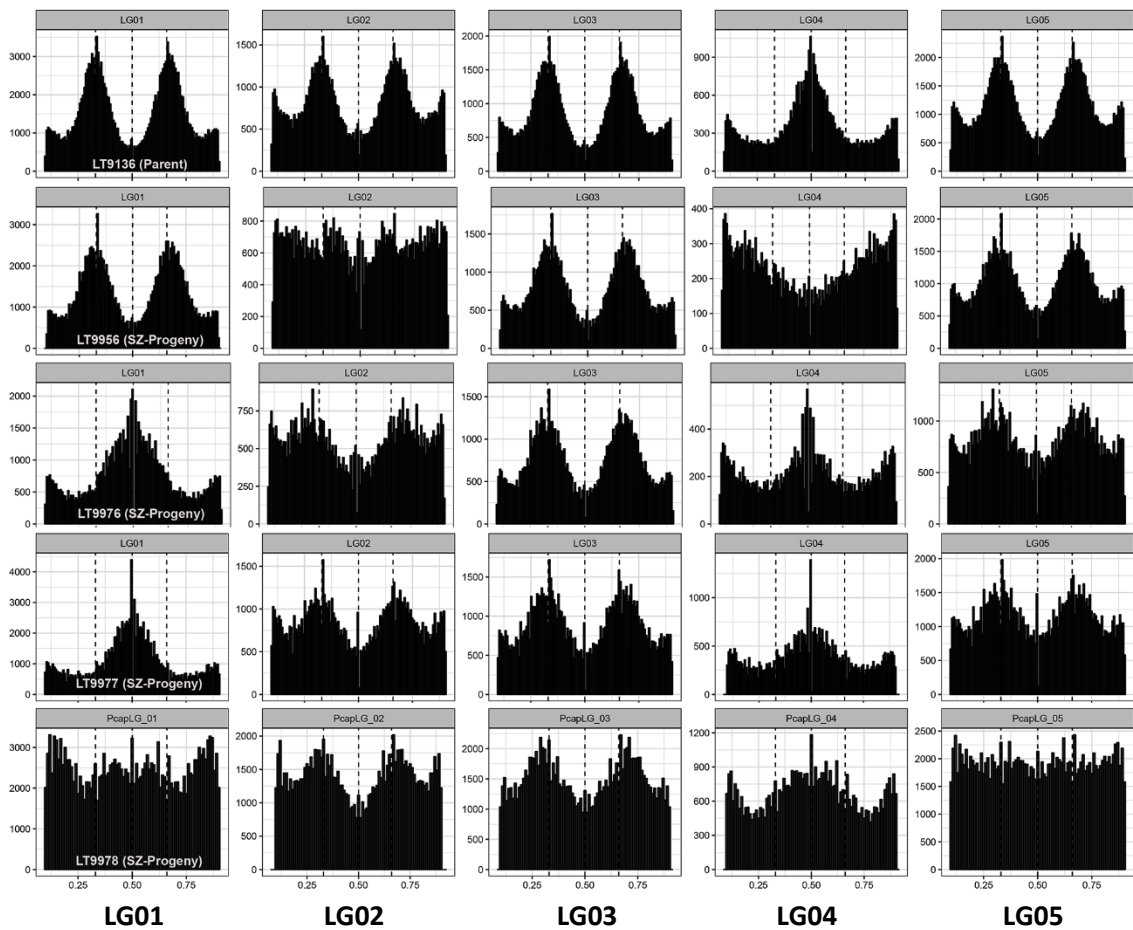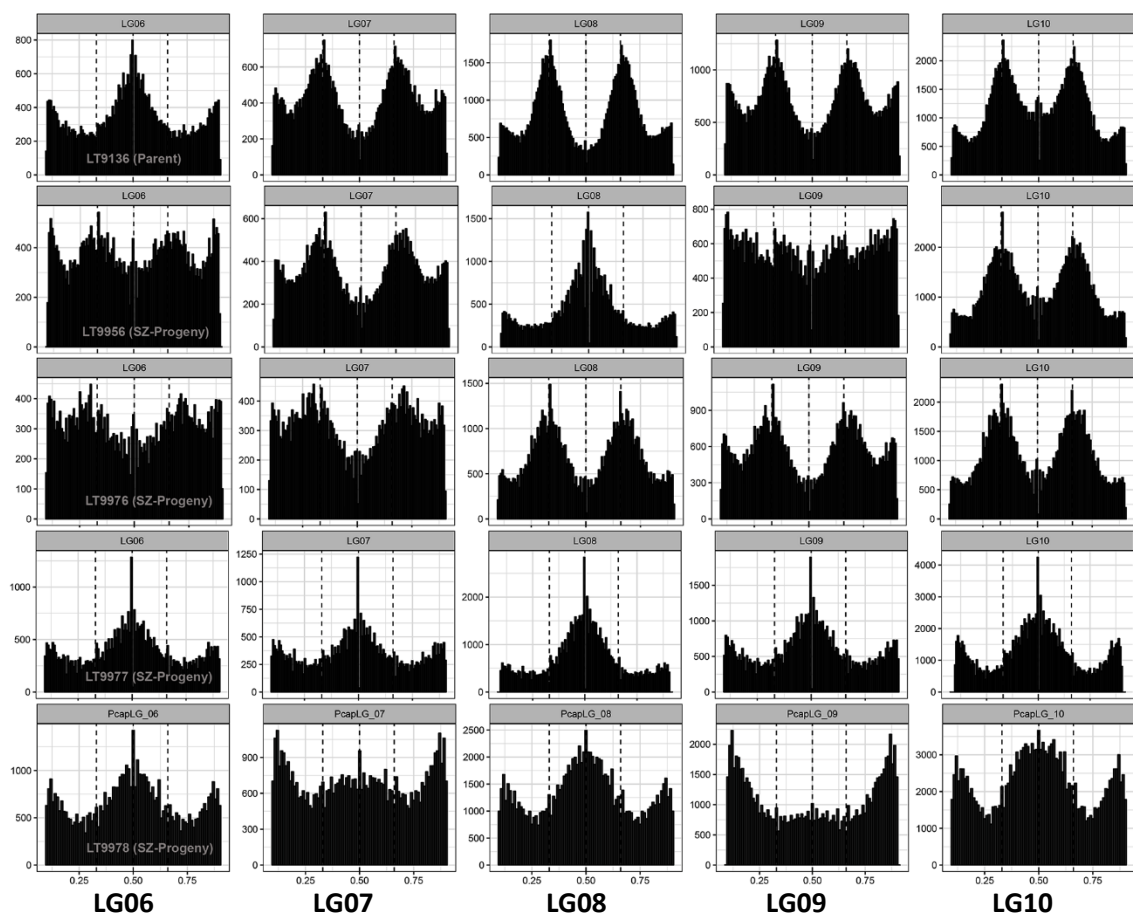

Supplement: S4 Fig — (PDF) [file pone.0227250.s004.pdf]
