# Supplementary material for: Dynamic Extreme Aneuploidy (DEA) in the vegetable pathogen Phytophthora capsici and the potential for rapid asexual evolution
Source: PLoS One. 2020 Jan 7;15(1):e0227250. doi: 10.1371/journal.pone.0227250 (PMC6946123; doi:10.1371/journal.pone.0227250)
Supplement: S3 Fig — (PDF) [file pone.0227250.s003.pdf]

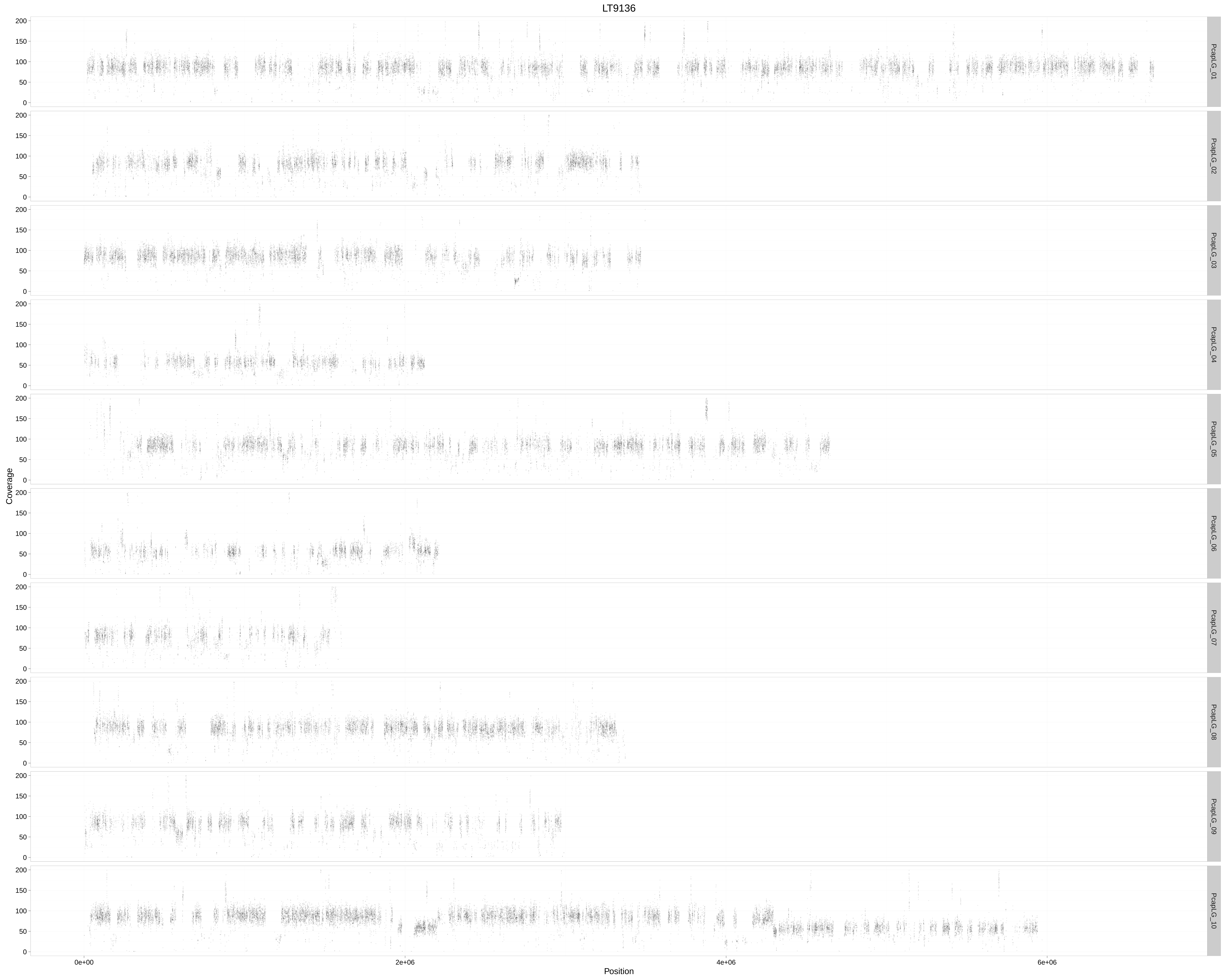

LT9136
Coverage
Position
0e+00
2e+06
4e+06
6e+06
0
50
100
150
200
PcapLG_01
PcapLG_02
PcapLG_03
PcapLG_04
PcapLG_05
PcapLG_06
PcapLG_07
PcapLG_08
PcapLG_09
PcapLG_10

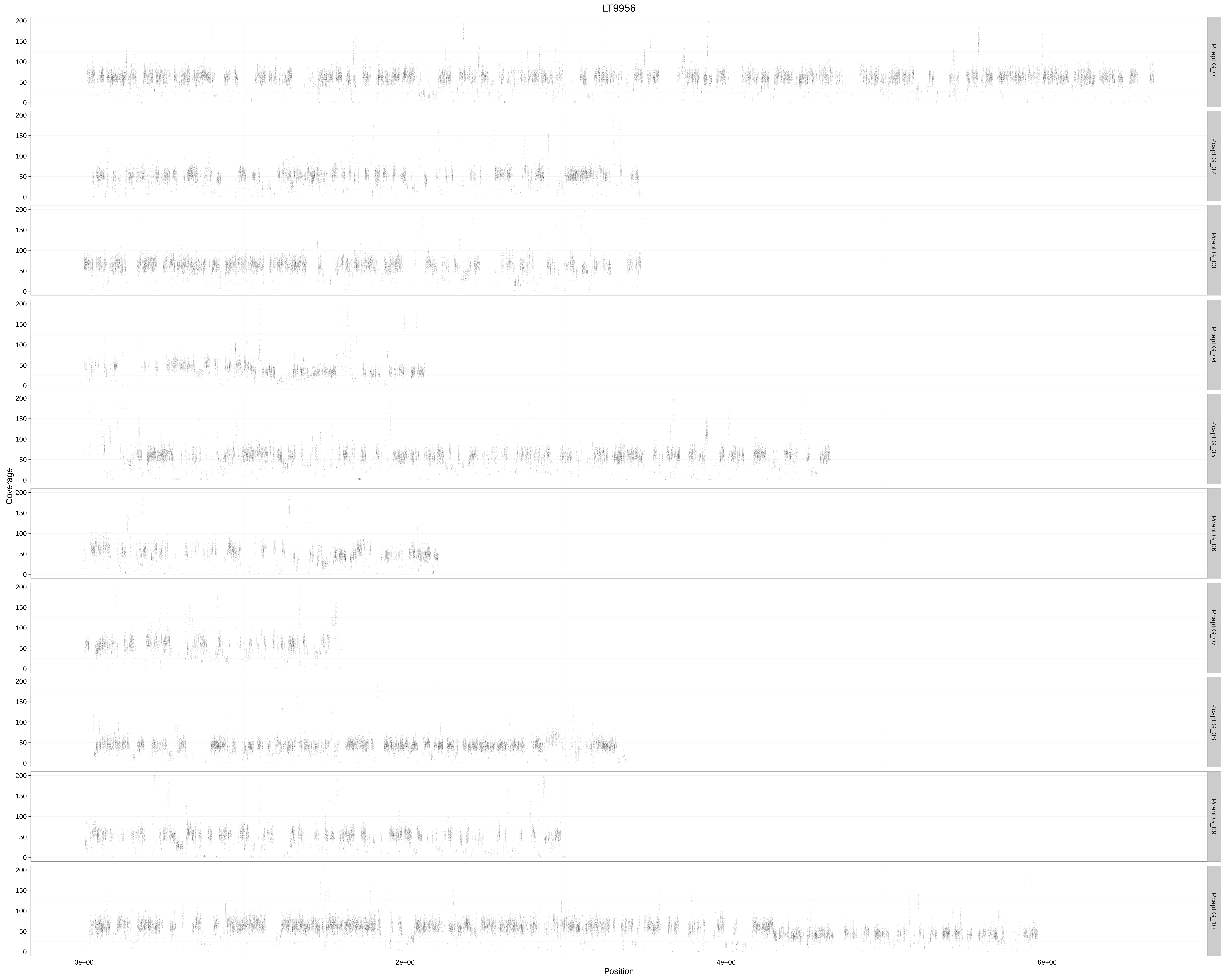

LT9956
Coverage
Position
0e+00
2e+06
4e+06
6e+06
0
50
100
150
200
PcapLG_01
PcapLG_02
PcapLG_03
PcapLG_04
PcapLG_05
PcapLG_06
PcapLG_07
PcapLG_08
PcapLG_09
PcapLG_10

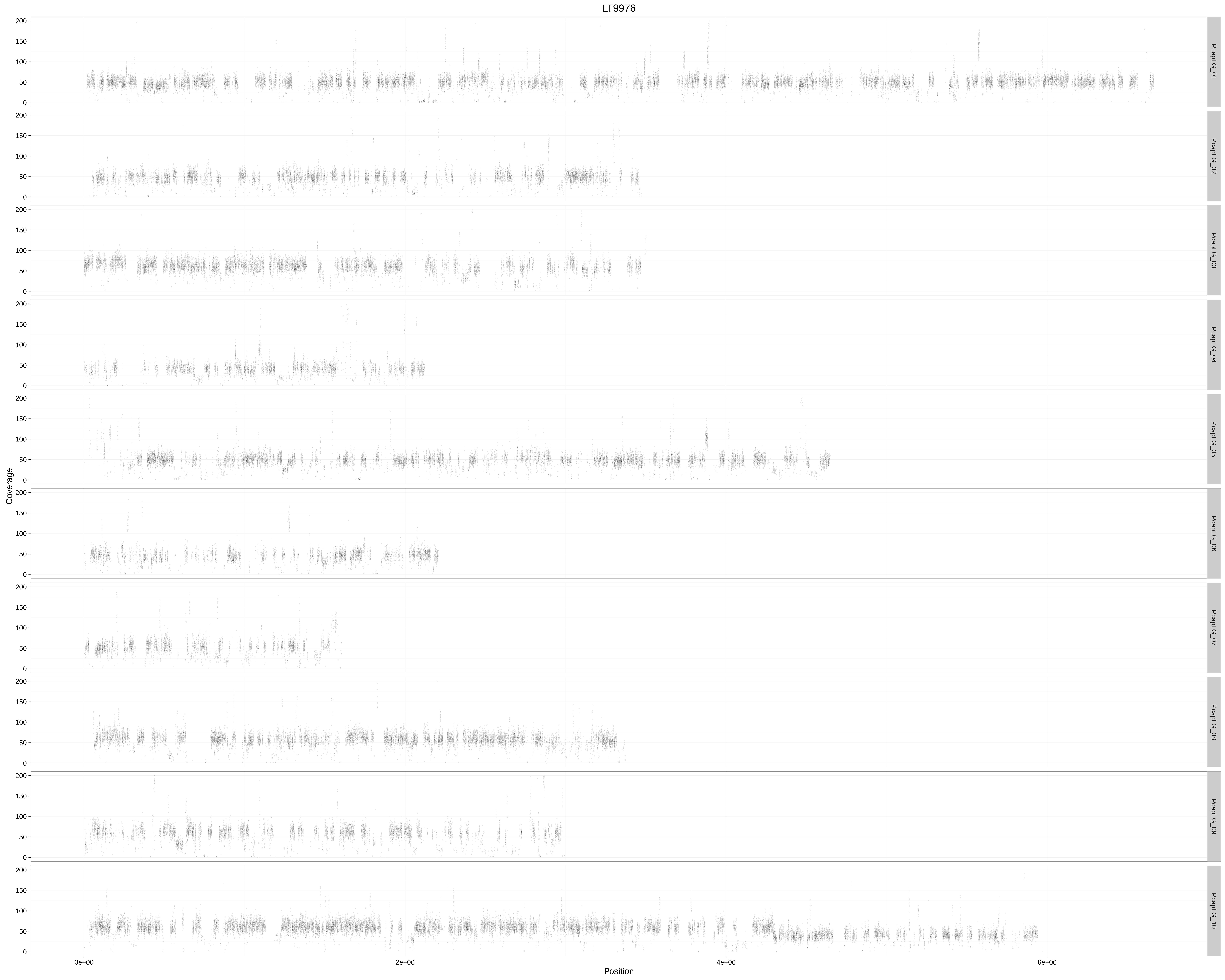

LT9976
Coverage
Position
PcapLG_01
PcapLG_02
PcapLG_03
PcapLG_04
PcapLG_05
PcapLG_06
PcapLG_07
PcapLG_08
PcapLG_09
PcapLG_10
0e+00
2e+06
4e+06
6e+06
0
50
100
150
200

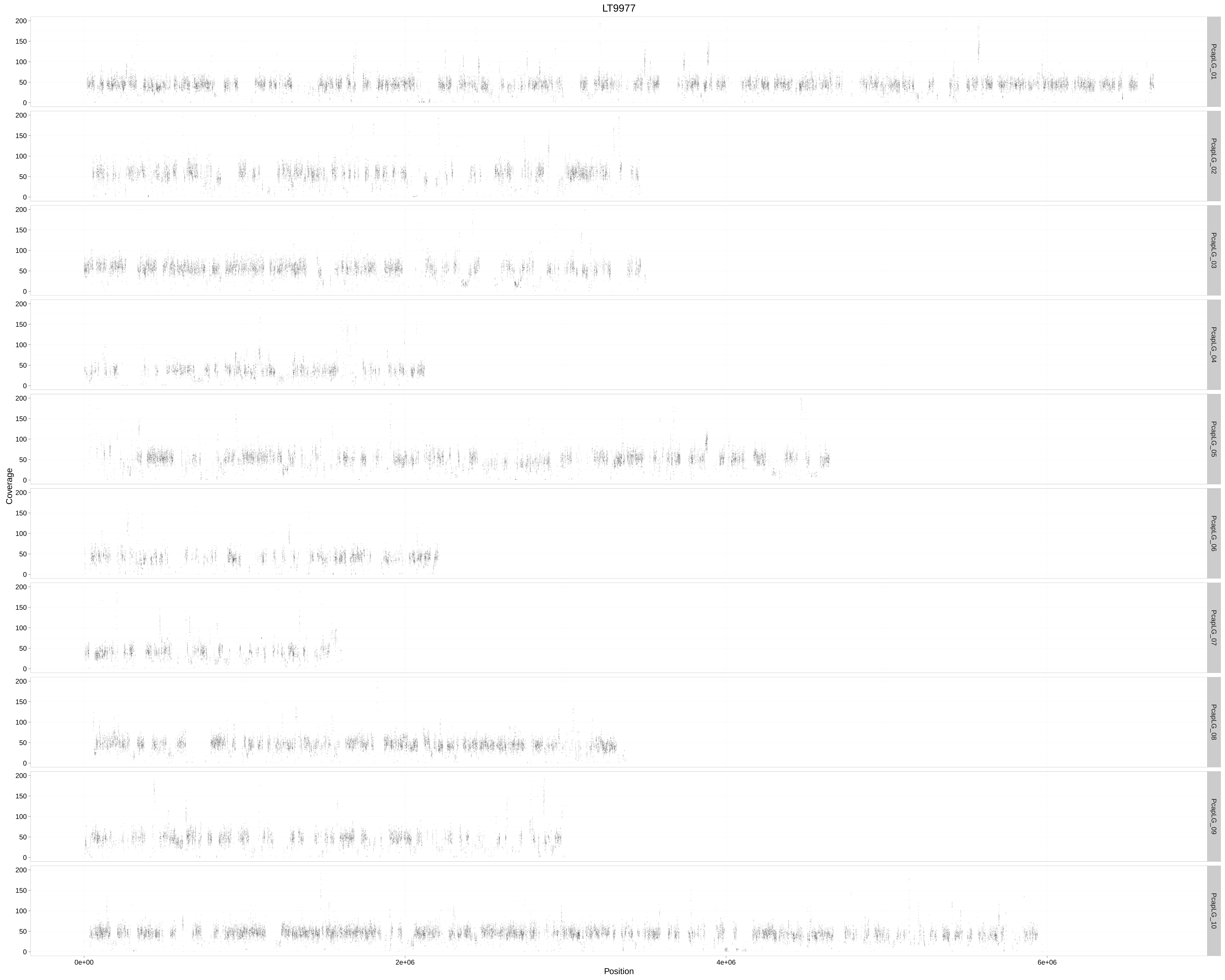

LT9977
Coverage
Position
0e+00
2e+06
4e+06
6e+06
0
50
100
150
200
PcapLG_01
PcapLG_02
PcapLG_03
PcapLG_04
PcapLG_05
PcapLG_06
PcapLG_07
PcapLG_08
PcapLG_09
PcapLG_10

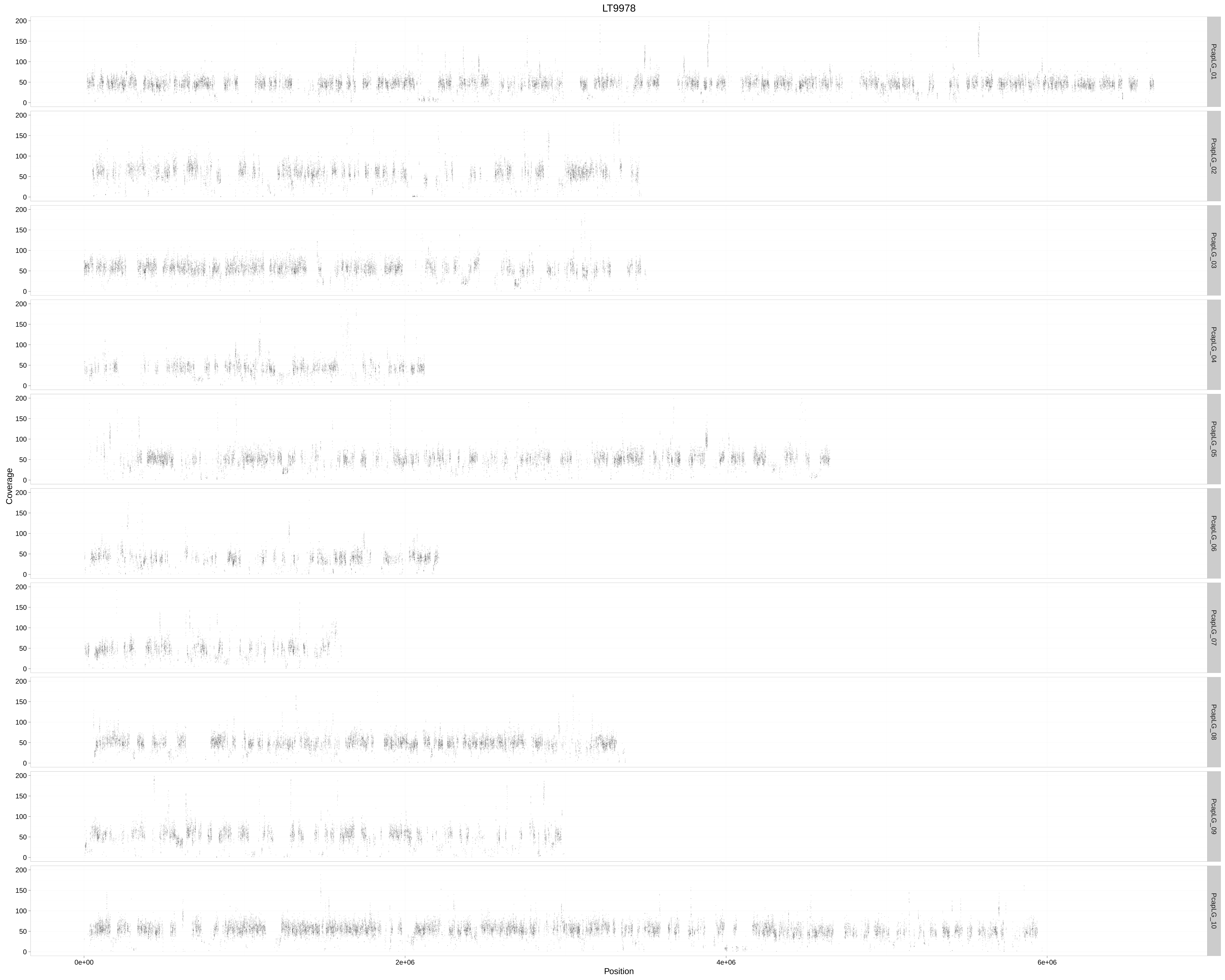
LT9978
Coverage
Position
0e+00
2e+06
4e+06
6e+06
0
50
100
150
200
PcapLG_01
PcapLG_02
PcapLG_03
PcapLG_04
PcapLG_05
PcapLG_06
PcapLG_07
PcapLG_08
PcapLG_09
PcapLG_10
